# Supplementary material for: Haplotype-resolved DiMeLo-seq maps centromeric chromatin in a complete diploid human genome
Source: Cell Genom. 2026 Aug 6;6(8):101324. doi: 10.1016/j.xgen.2026.101324 (PMC13477032; doi:10.1016/j.xgen.2026.101324)
Supplement: Supplementary file 1 [file mmc1.pdf]

Cell Genomics, Volume *6*

Supplemental information

Haplotype-resolved DiMeLo-seq

maps centromeric chromatin

in a complete diploid human genome

Yuan Xu, Hailey Loucks, Julian Menendez, Fedor Ryabov, Julian K. Lucas, Monika Cechova, Luke Morina, Emily Xu, Danilo Dubocanin, Cy Chittenden, Mobin Asri, Ivo Violich, Christian Ortiz, Joshua M.V. Gardner, Todd Hillaker, Sara O'Rourke, Brandy McNulty, Tamara A. Potapova, Matthew W. Mitchell, Jacob P. Schwartz, Aaron F. Straight, Jennifer L. Gerton, Winston Timp, Ivan A. Alexandrov, Nicolas Altemose, and Karen H. Miga

Supplemental Information accompanying the paper

“Haplotype-resolved centromeric chromatin organization from a complete diploid human genome”

Figure S1: Diploid centromeric satellite annotations for HG002, related to Figure 1 2
Figure S2: Chromosome 9 HSat3 variation, related to Figure 1 3
Figure S3: Chromosome 1 HSat SF variation, related to Figure 1 4
Figure S4: Chromosome 3 and 4 Alpha and HSat1A variation, related to Figure 1 5
Figure S5: Moddotplot with paired 1D moddotplot track, related to Figure 1 6
Figure S6: HOR Structural Variant (StV) profiles, related to Figure 1 7
Figure S7: Chromosome 13 Structural Variant (StV) and HORhap analysis, related to Figure 1 8
Figure S8: CDR p-arm proximal regions, related to Figure 1 9
Figure S9: CDR association with homogenized regions, related to Figure 1 10
Figure S10: Total CDR span per haplotype in early passage LCL (HG002), related to Figure 1 11
Figure S11: CDR (subCDR and span) vs active array size, related to Figure 1 12
Figure S12: Coverage estimates of adaptive sampling, related to Figure 2 13
Figure S13: Clustering methylation data into groups, related to Figure 2 14
Figure S14: Fiber-seq nucleosome organization relative to CENP-B motifs, related to Figure 2 15
Figure S15: CENP-C DiMeLo-seq 6mA density (early passage), related to Figure 2 16
Figure S16: Methylation frequency across active arrays in early vs late passage, related to Figure 3 17
Figure S17: Difference in sub-CDR structure in early vs late passage, related to Figure 3 18
Figure S18: Enrichment within sub-CDRs across early- and late-passage, related to Figure 3 19
Figure S19: Comparison between HG002 early passage and hiPSC CDRs, related to Figure 3 20
Figure S20: CENP-A DiMeLo-seq Density in hiPSCs, related to Figure 3 21
Figure S21: CENP-A DiMeLo-seq density in late passage lines, related to Figure 3 22
Figure S22: hiPSC array CpG density and CDR prediction, related to Figure 3 23

References 24

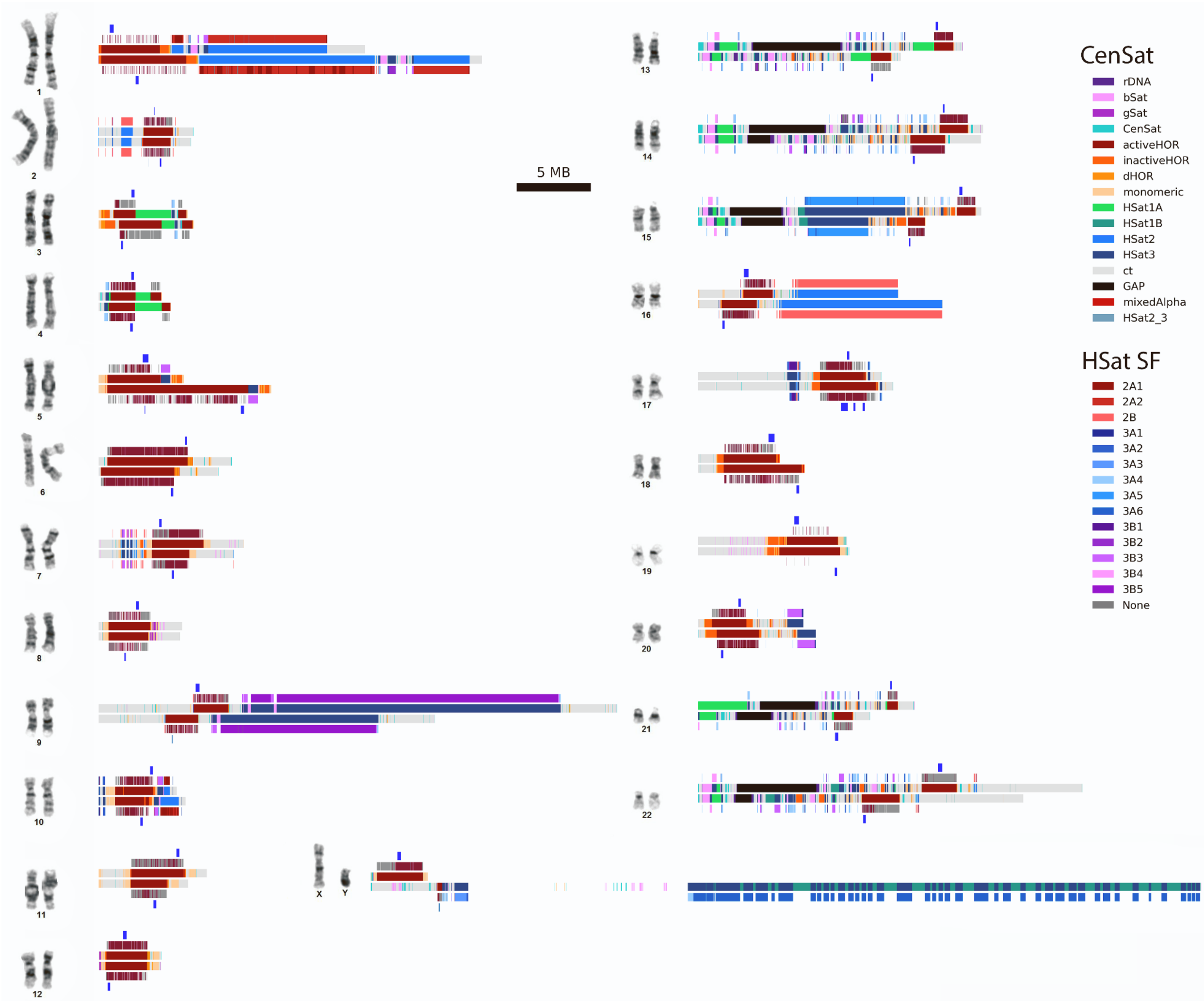

Figure S1: Diploid centromeric satellite annotations for HG002, related to Figure 1

HG002 g-banding karyotype images displaying centromeric satellite (censat) annotation tracks for all chromosomes in the diploid HG002 genome, generated using an automated annotation method. For each chromosome, the maternal haplotype is shown on top and the paternal haplotype on the bottom. Annotation tracks are arranged symmetrically from the center outward and include: (1) cenSat family annotations, (2) alpha-satellite higher-order repeat (HOR) haplotypes and satellite superfamilies (HSat SF), and (3) centromeric dip regions (highlighted in blue). Alpha-satellite HOR haplotypes are colored on a gradient from younger HORs (red) to older HORs (grey)

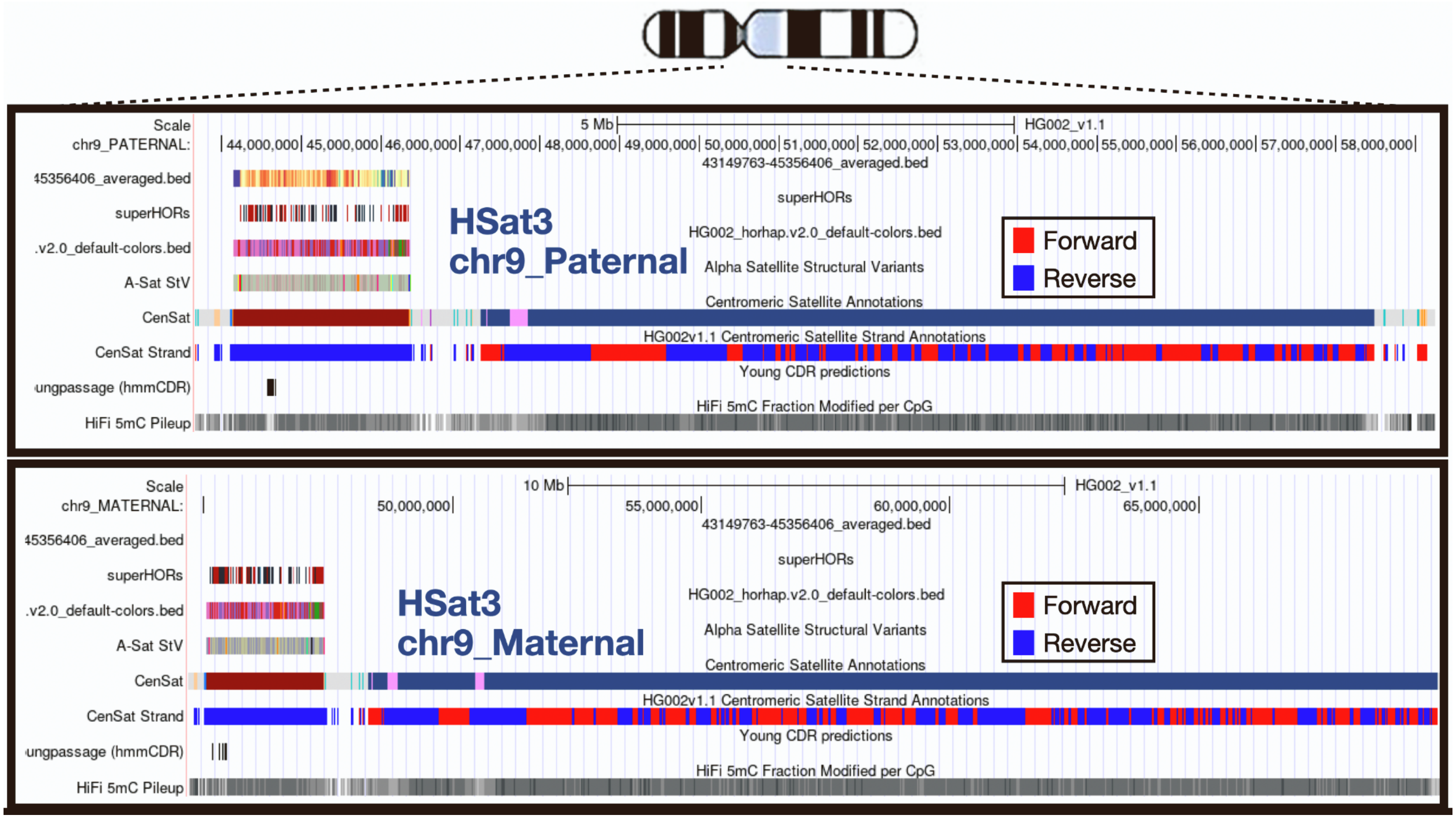

Figure S2: Chromosome 9 HSat3 variation, related to Figure 1

UCSC Genome Browser track hub [1] visualizations of the centromeric region of chromosome 9 for the maternal (chr9_maternal: 45–70 Mb) and paternal (chr9_paternal: 44–58 Mb) haplotypes, displaying cenSat satellite annotations. Unlike most satellite families, which are arranged head-to-tail in a single orientation (e.g., alpha satellite, dark red, cenSat track), Human Satellite 3 (HSat3, dark blue) on chromosome 9 exhibits both length variation and major shifts in repeat orientation across the array. Forward and reverse HSat3 orientations are shown in red and blue, respectively, revealing multiple megabase-scale strand domains arranged in large blocks. The order and polarity of these orientation segments differ between maternal and paternal chromosomes, consistent with large internal inversions and strand-polarized repeat substructure across the HSat3 array.

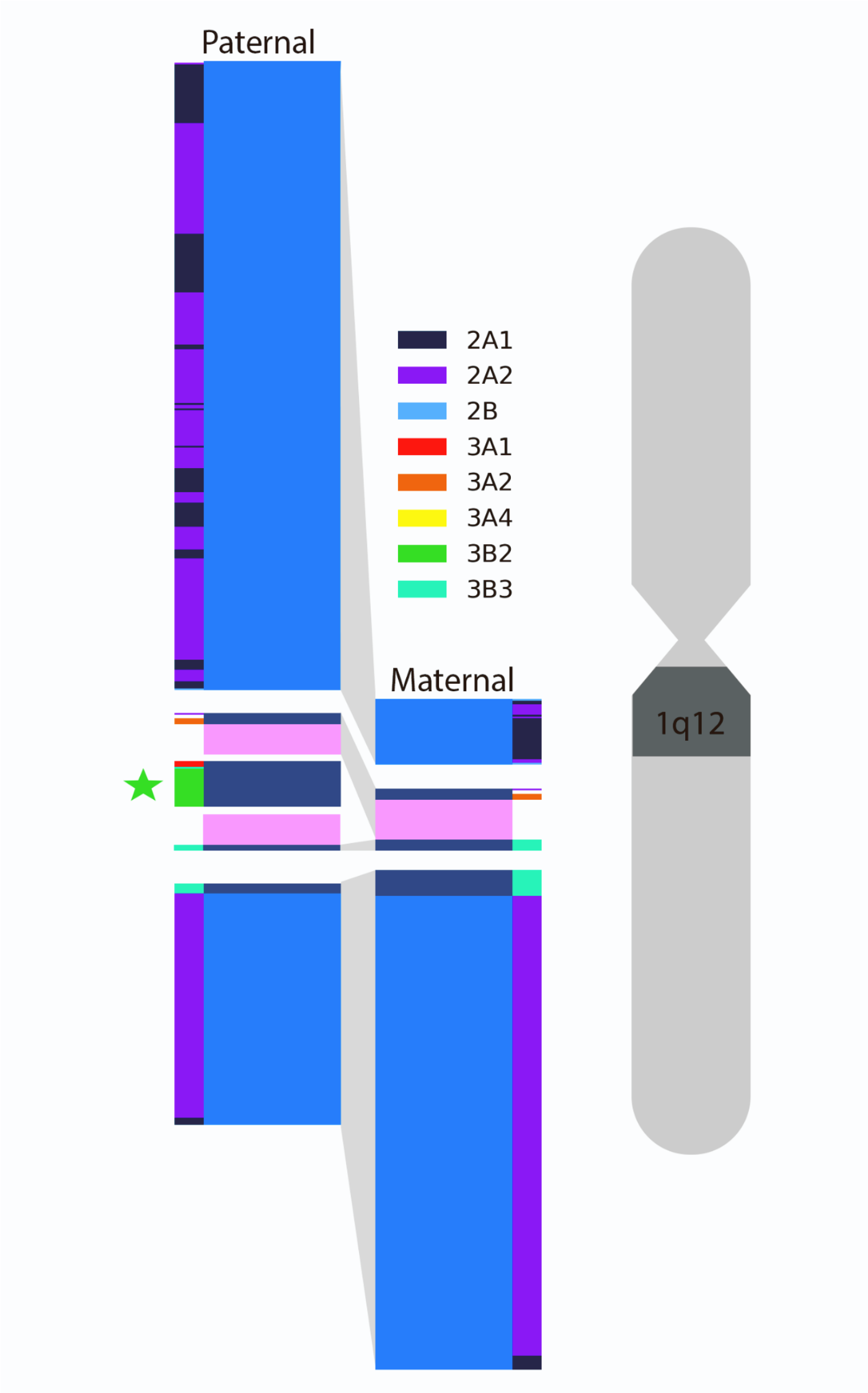

Figure S3: Chromosome 1 HSat SF variation, related to Figure 1

Comparison of Chromosome 1q12 region between paternal and maternal haplotypes, large Hsat2 arrays have been truncated and are not to scale. Outer columns represent HSat superfamilies, and thicker inner columns represent CenSat annotation. Homologous arrays are connected. HSat2 on chromosome 1 differs by 6.74 Mb between homologs and contains distinct sequence compositions. The paternal haplotype contains a unique HSat3B2 array marked with a star and an adjacent beta-satellite sequence, while the paternal haplotype contains an additional ~500 kb polymorphic block of HSat3 with an adjacent beta-satellite sequence.

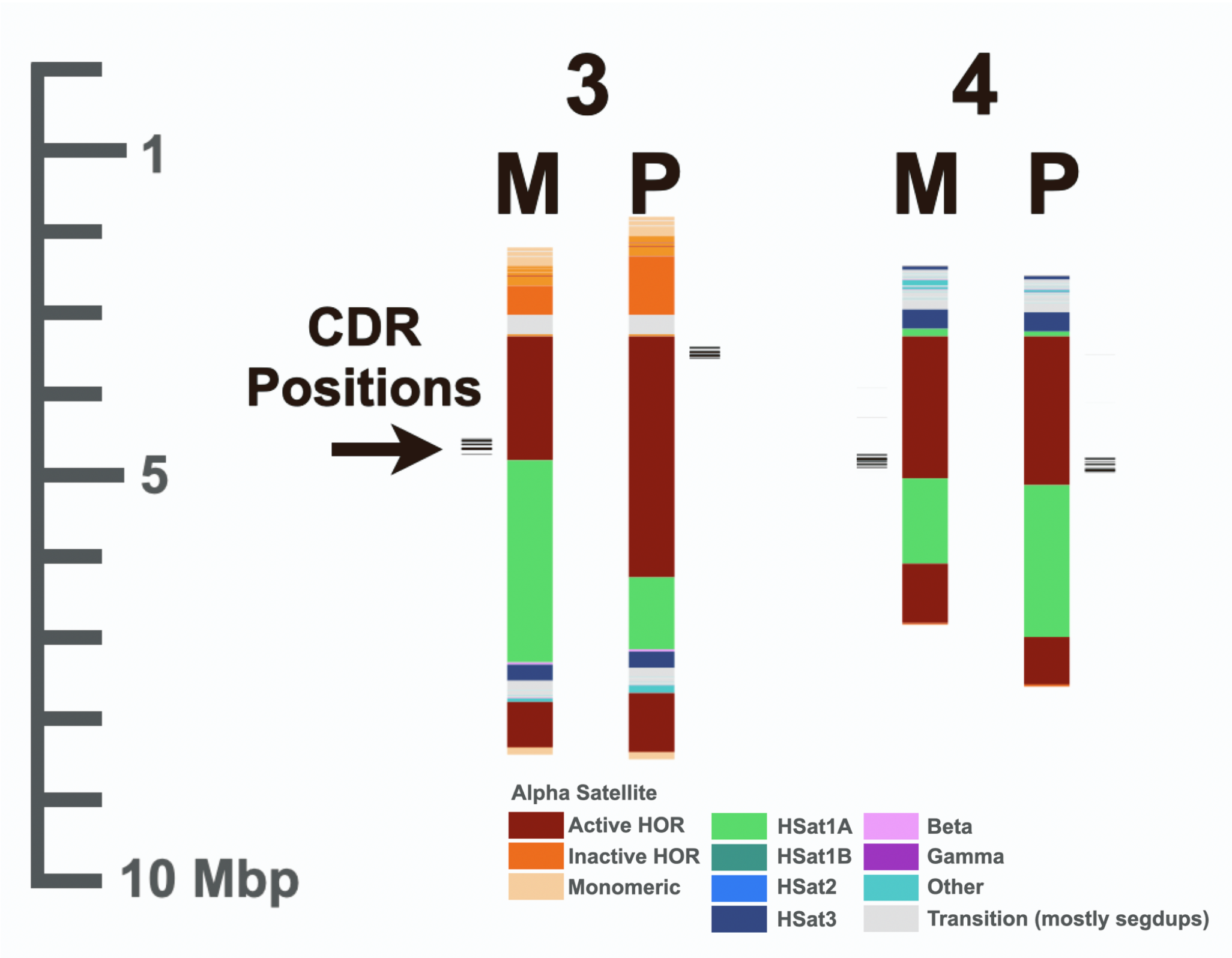

Figure S4: Chromosome 3 and 4 Alpha and HSat1A variation, related to Figure 1

CenSat annotations are shown for the maternal (M) and paternal (P) haplotypes of chromosomes 3 and 4, with satellite subfamily identity indicated by color (see key). Scale bars are provided for each haplotype. Centromere dip regions (CDRs), which mark the position of the kinetochore-associated active alpha-satellite (αSat) array, are indicated by black tick marks; the CDR on chromosome 3M is highlighted by an arrow. These chromosomes illustrate two distinct forms of centromeric structural variation. First, interspersed HSat1A arrays (green) are embedded within the active αSat arrays on both chromosomes, splitting the array into discontinuous blocks and differing in size between haplotypes (~0.61 Mb on chr3; ~1.1 Mb on chr4). Second, the active αSat arrays differ in total length between haplotypes, reflecting haplotype-specific restructuring of kinetochore-associated centromeric domains. On chromosome 3, additional complexity is observed in the form of interspersed centromere transition (CT) regions containing lncRNA candidate loci (grey), as well as HSat3 and other smaller centromeric satellite classes, indicating a more structurally heterogeneous array organization relative to chromosome 4.

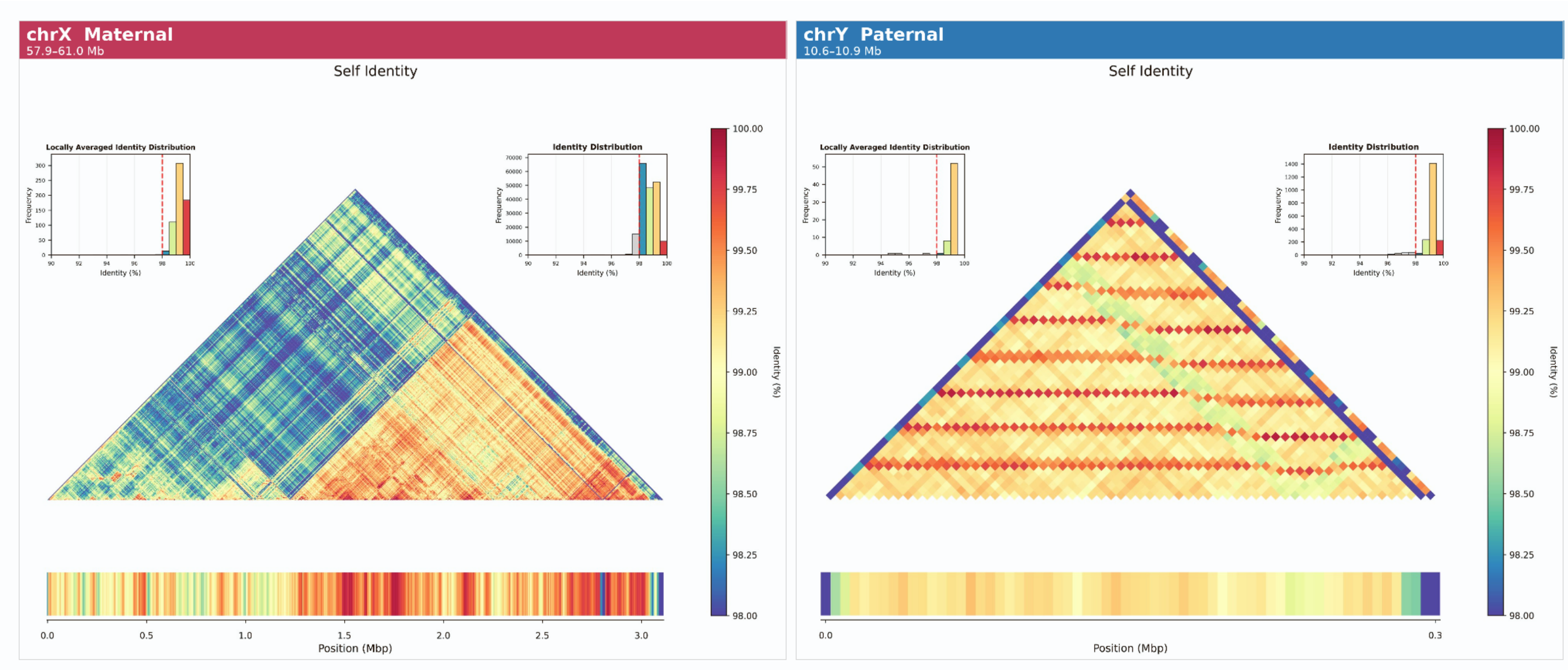

Figure S5: Moddotplot with paired 1D moddotplot track, related to Figure 1
Triangular self-identity heatmaps are shown for the active alpha-satellite HOR (αSat) arrays of X and Y chromosomes. Active arrays were identified from the HG002v1.1 assembly using the cenSat v2.0 annotation by selecting entries with the suffix "H1L." Adjacent intervals within 200 kb were merged, and FASTA sequences for each array were extracted and input to ModDotPlot[2] (window size = 5 kb, δ = 0) to generate all-vs-all pairwise identity values. Identity values were locally averaged across the two nearest neighboring windows on each side using a custom Python script. The resulting values are displayed on a color scale ranging from a fixed lower bound of 90% identity (or the 10th percentile of observed values if higher) to 100%, with warmer colors (red/orange) indicating higher identity and cooler colors (blue/purple) indicating lower identity. The bar below each triangle represents the locally averaged identity at each position along the array. Inset histograms show the distributions of locally averaged identity (left) and raw all-vs-all pairwise identity (right); the red dashed line indicates the mean. Genomic coordinates (GRCh38) are indicated in the panel header.

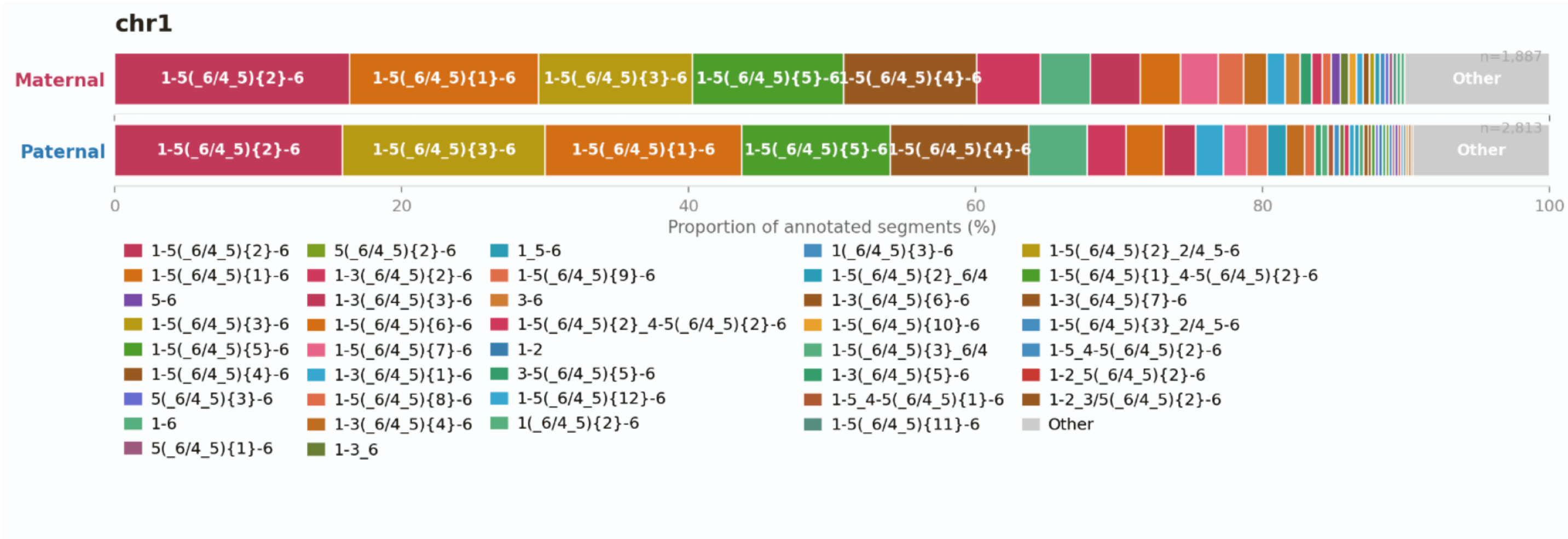

Figure S6: HOR Structural Variant (StV) profiles, related to Figure 1
Stacked horizontal bar charts showing the proportional distribution of alpha-satellite HOR structural variants for chromosome 1 (as an example, with all chromosomes referenced in Table S4), with maternal (top) and paternal (bottom) haplotypes shown separately. Each colored segment represents a unique HOR variant, labeled by its structural suffix (common chromosome-specific HOR prefix removed for clarity). Segment width reflects the proportion of total annotated array segments occupied by each variant. Variants with fewer than 5 are grouped into "Other" (grey). The total number of annotated segments per haplotype (n) is indicated at the right of each bar.

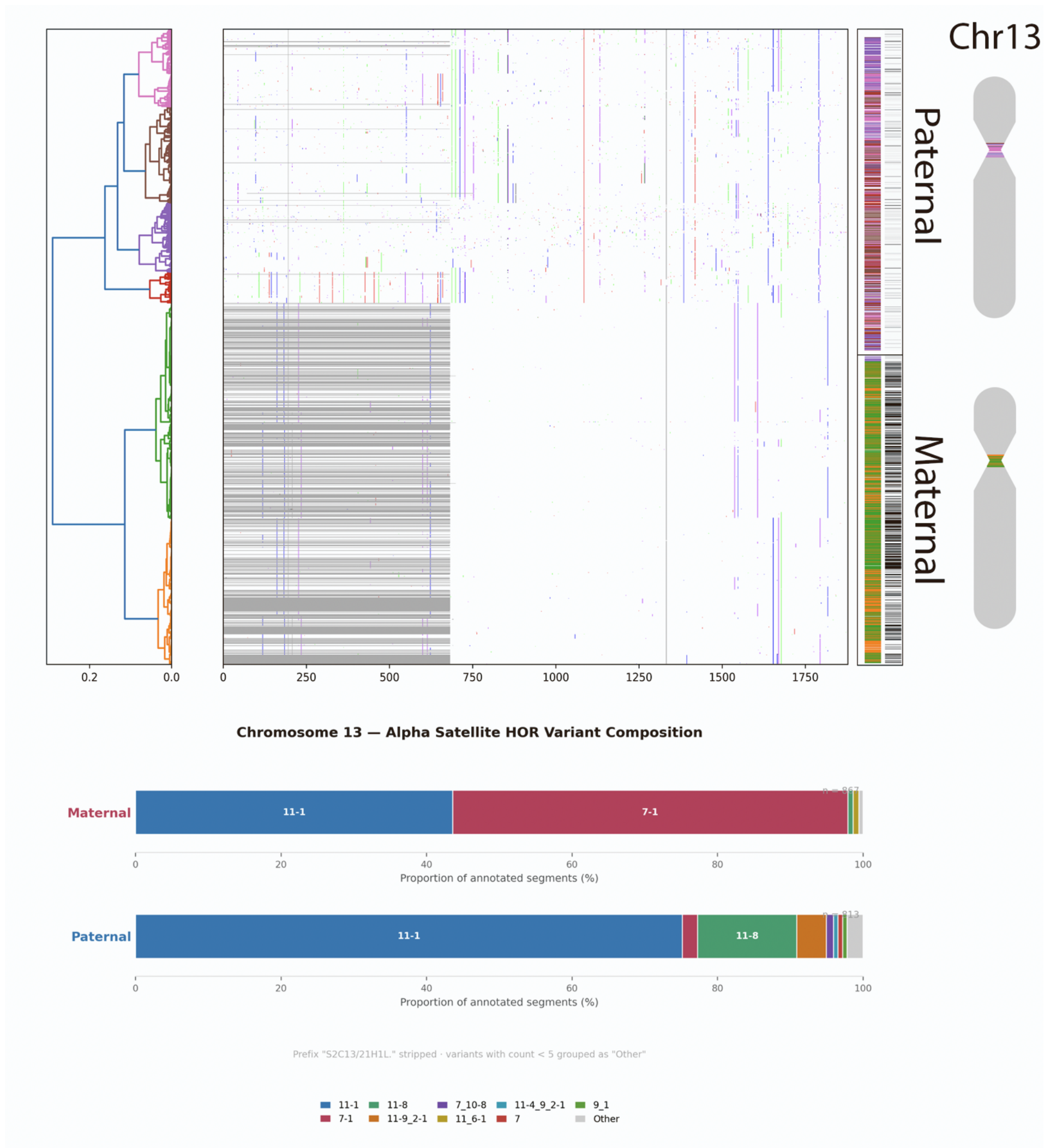

Figure S7: Chromosome 13 Structural Variant (StV) and HORhap analysis, related to Figure 1
Ward HORhap clustering for chromosome 13 in HG002. Sequences of the two most frequent HOR structural variants (StVs) (the full-length S2C13/21H1L.11-1 and S2C13/21H1L.7-1 with a deletion of four monomers) from maternal and paternal arrays were aligned together. Only positions that differ from the consensus are colored (center). HORs in the alignment are ordered according to the Ward dendrogram (left), which is split into six colored clades (HORhaps). On the right, paternal and maternal centromeres (shown one above the other) are displayed as HORhap tracks with the corresponding colors (left) and tracks showing the positions of 7-mer StVs (right). On the far right, the positions of the centromeres within the entire chromosomes are indicated. The specificity of HORhaps to homologs is evident: the paternal homolog consists only of pink, brown, purple, and red HORhaps, corresponding to the upper major branch of the dendrogram, whereas the maternal homolog consists almost entirely of green and orange HORhaps, corresponding to the lower major branch, with only the very tip of the p-arm (upper end) represented by HORhaps from the upper major branch, and deletions are visible in grey.

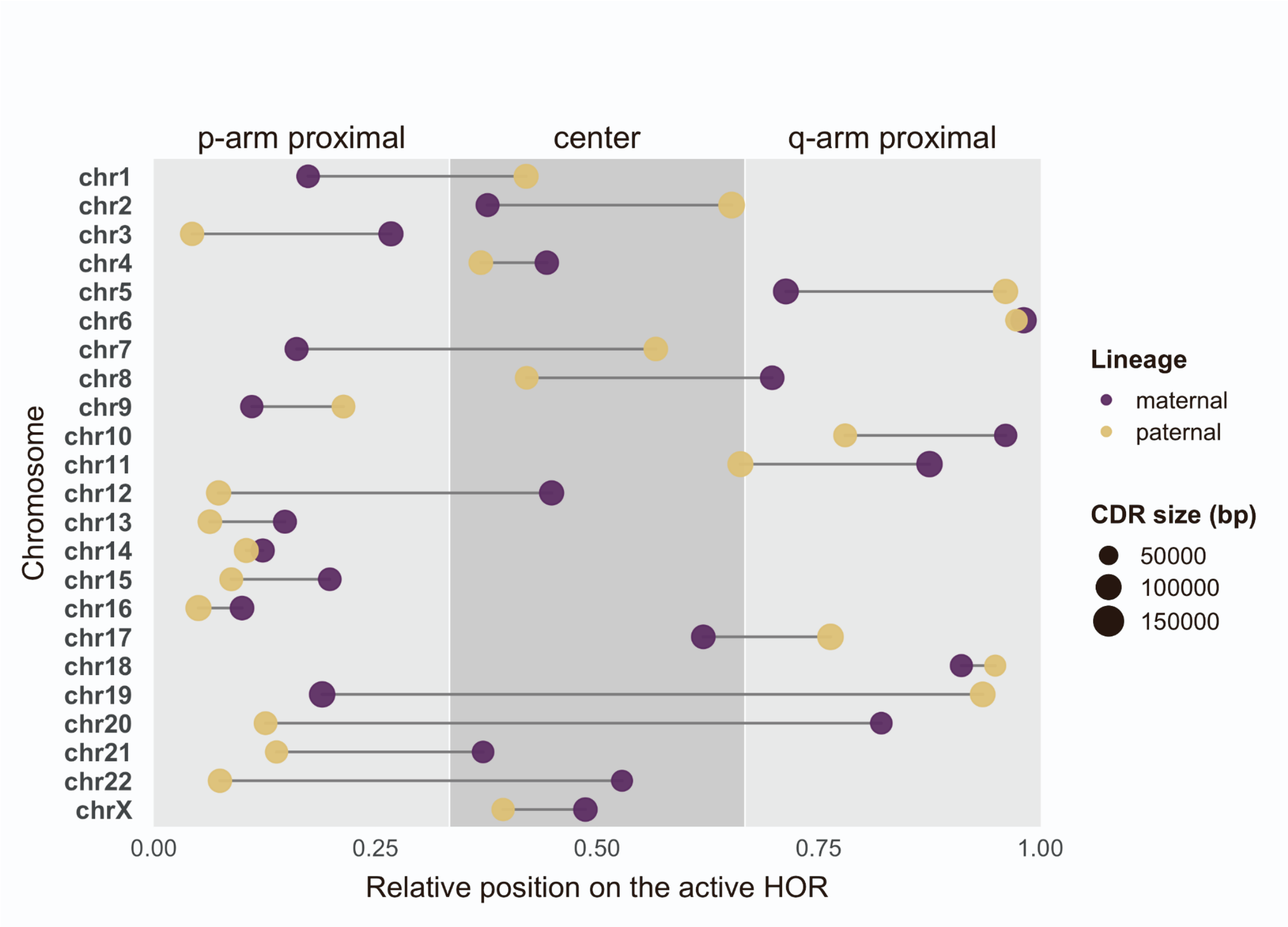

Figure S8: CDR p-arm proximal regions, related to Figure 1

The relative position and size of CDRs within the active higher-order repeat array for each chromosome. The relative location of the CDR midpoint within the active array (x-axis; split equally between p-arm proximal, center, and q-arm proximal location) is noted for each chromosome (y-axis). The size of the dot is relative to the size of the CDR, here defined as the sum of all the subCDRs. Finally, the maternal and paternal CDRs for each homologous chromosome (including X and Y) are connected by a horizontal line. The p-arm proximal location is the most frequent out of the three possibilities.

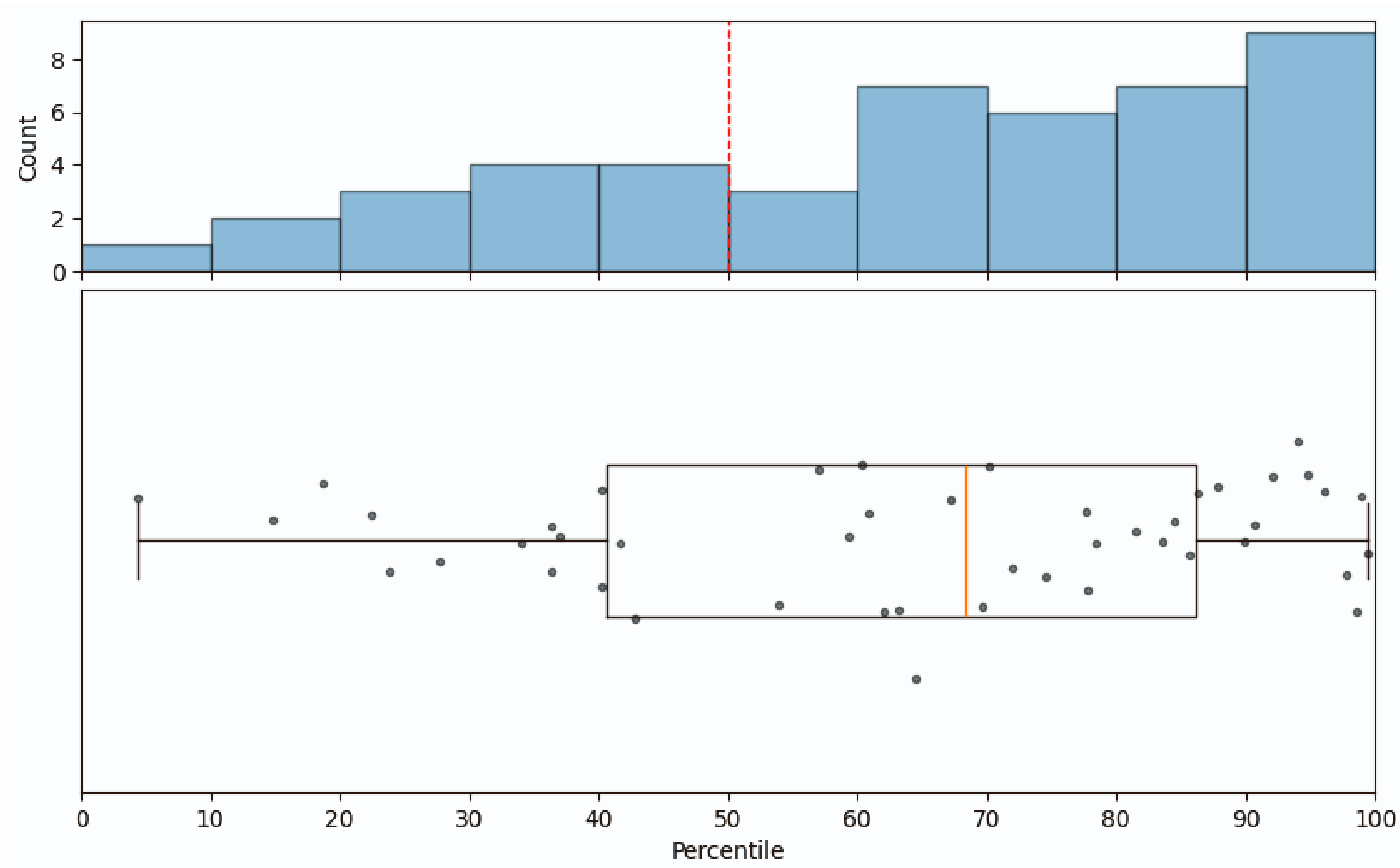

Figure S9: CDR association with homogenized regions, related to Figure 1
CDR local identity as a percentile of the active alpha array distribution. For each chromosomal CDR, defined as the span between the two outermost subCDRs on a given haplotype, mean local identity was calculated across overlapping bins. To assess relative self-similarity, each CDR's mean local identity was compared to the background distribution of local identities across the whole active array. Each chromosome was assigned a percentile score reflecting where its CDR mean local identity falls within the active array distribution. The bottom panel shows per-chromosome CDR percentiles as a scatter-boxplot, with the top panel displaying the corresponding distribution as a histogram.

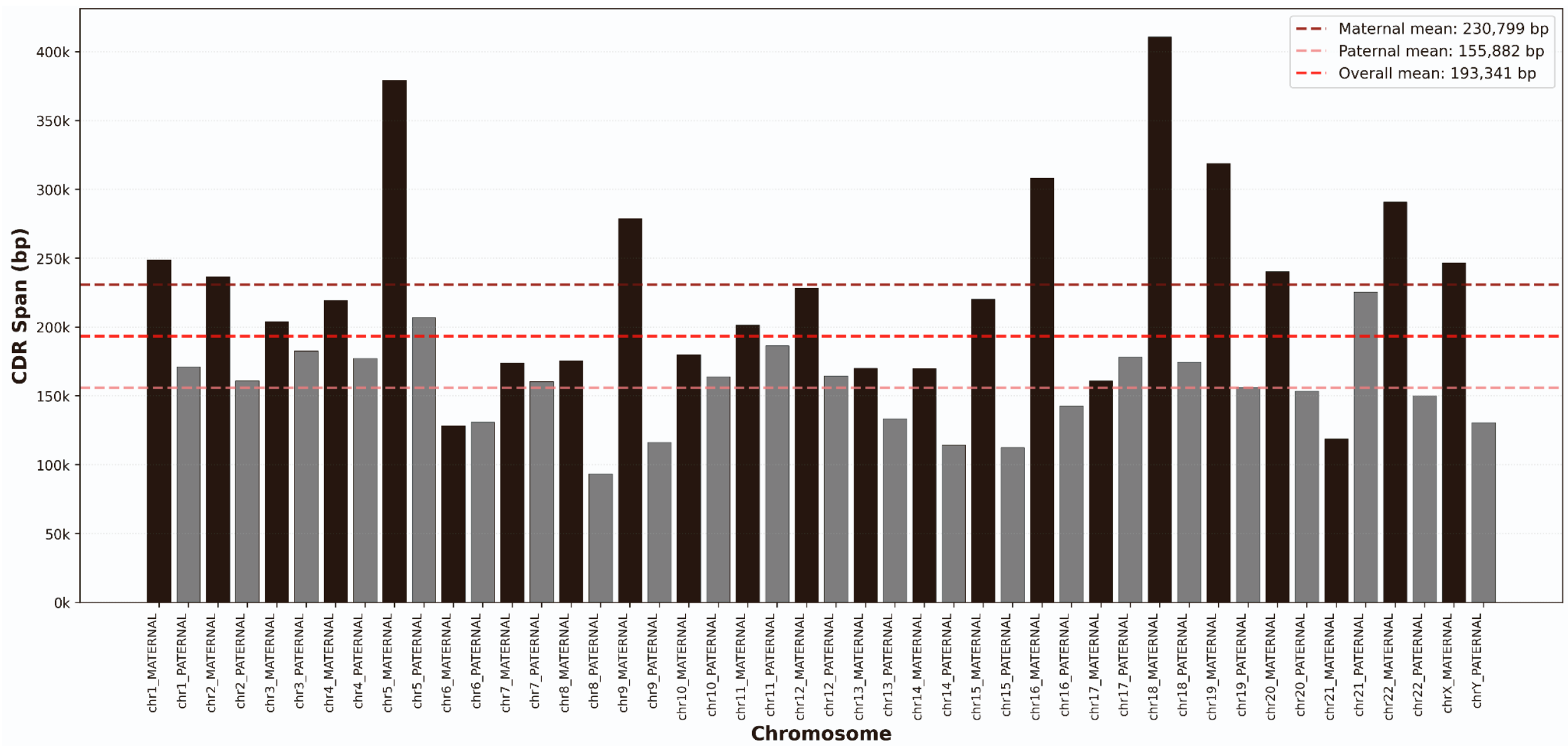

Figure S10: Total CDR span per haplotype in early passage LCL (HG002), related to Figure 1
Stacked bar plots showing the aggregate CDR span (kb) per haplotype across all 46 phased assemblies (chr1–chrY), as called by hmmCDR from DiMeLo-seq CENP-A data in the young passage LCL. Each bar represents a single haplotype (M, maternal; P, paternal), with individual stacked segments corresponding to discrete CDR subdomains; segment shading cycles from light to dark grey to distinguish adjacent subdomains. Horizontal reference lines indicate the mean aggregate CDR span across all maternal haplotypes (dark red dotted), all paternal haplotypes (light red dotted), and all haplotypes combined (red dashed). Mean aggregate CDR span was 87.4 kb (maternal), 89.4 kb (paternal), and 88.4 kb genome-wide across 254 total CDR domains.

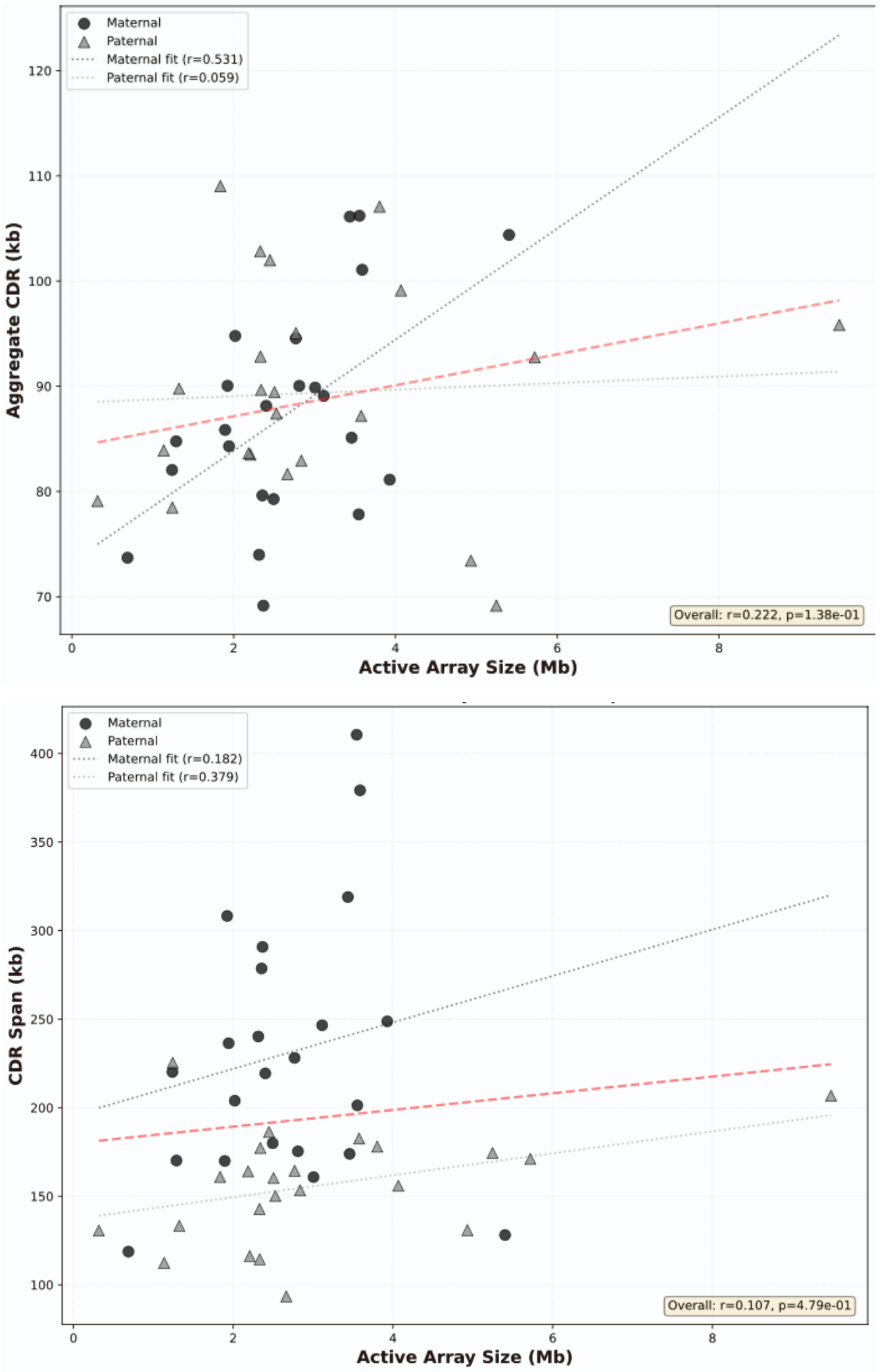

Figure S11: CDR (subCDR and span) vs active array size, related to Figure 1

Scatter plots showing the relationship between CDR length (x-axis) and total active αSat HOR array length (y-axis, Mb) for all chromosomes. Maternal haplotypes are shown as dark grey circles and paternal haplotypes as light grey squares; dashed lines indicate linear fits with associated Pearson r-values. (Top) Aggregated sub-CDR length, calculated as the sum of individual hypomethylated sub-CDR segments, excluding intervening regions between domains. The aggregated sub-CDR length is highly consistent genome-wide (mean ~90 kb; range: 69–109 kb) and shows no significant correlation with array size (maternal r = 0.059; paternal r = 0.059). (Bottom) Total CDR span, measured from the start of the first sub-CDR to the end of the last sub-CDR, including intervening non-hypomethylated sequence. Total span is more variable and shows a modest positive correlation with array size (maternal r = 0.182; paternal r = 0.379). Together, these plots demonstrate that while CDR span varies across haplotypes and chromosomes, the aggregate length of the hypomethylated sub-CDR core is remarkably stable and decoupled from the size of the underlying active HOR array.

A.

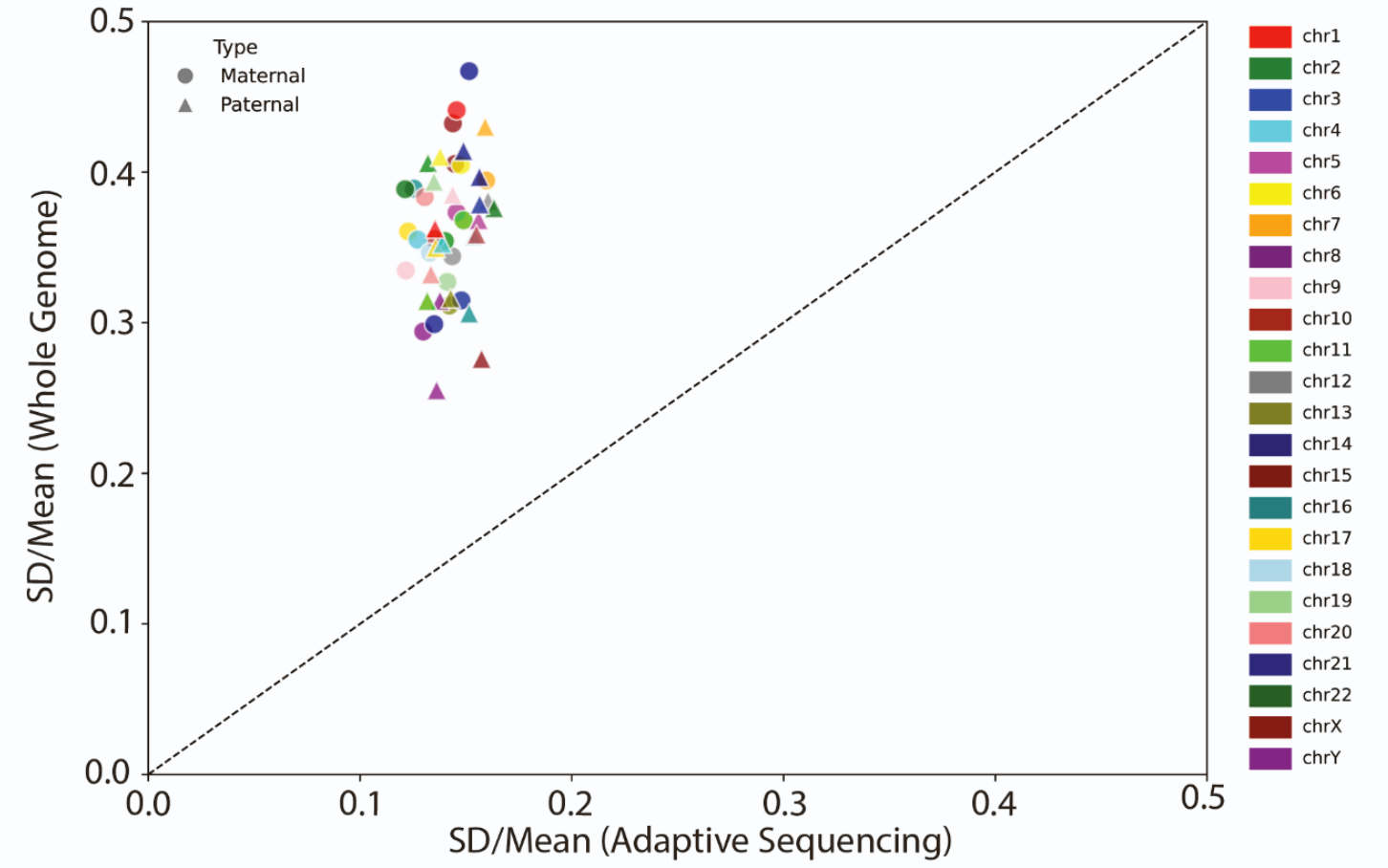

B.

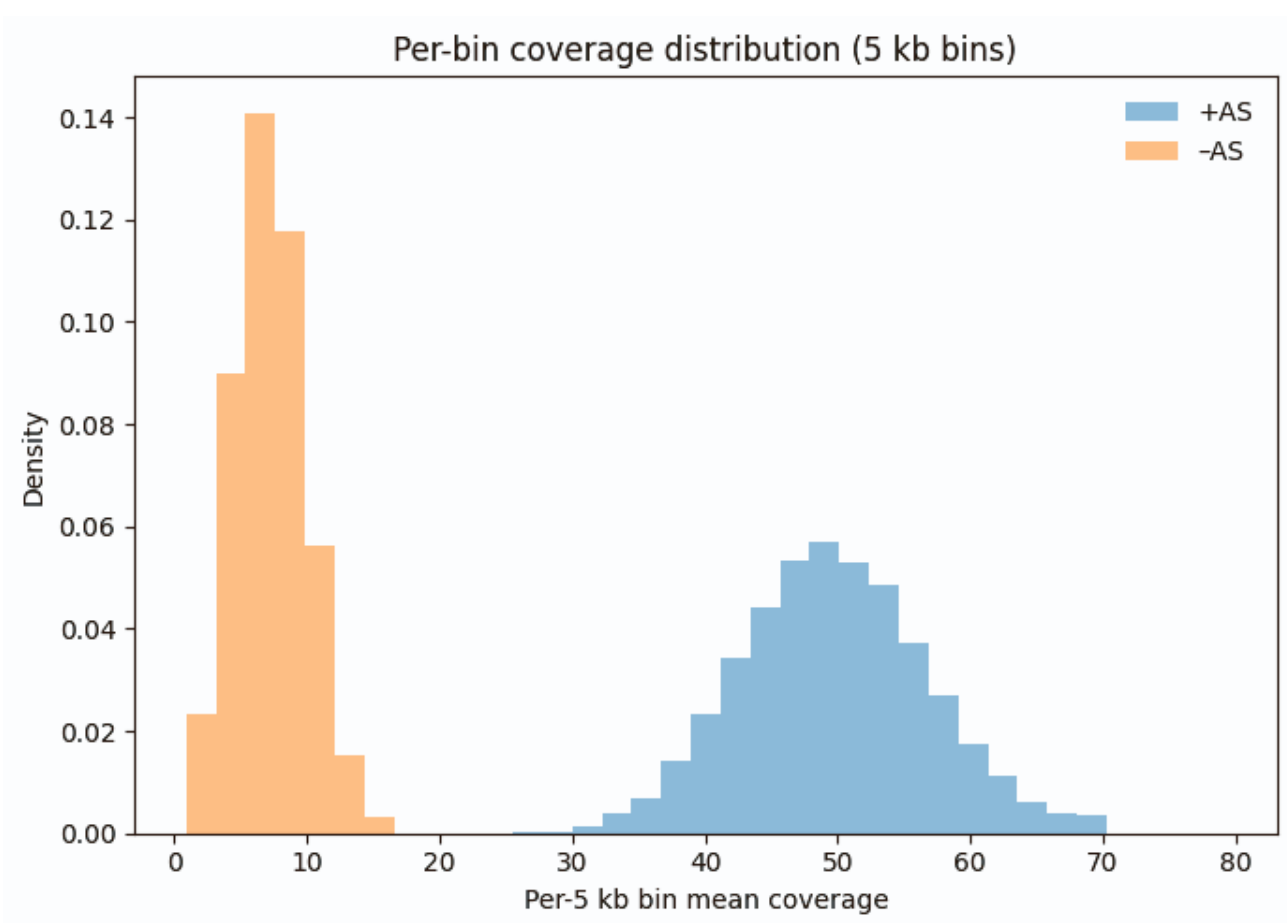

Figure S12: Coverage estimates of adaptive sampling, related to Figure 2

Adaptive sampling achieves uniform coverage across centromeric arrays. (A**)** Scatter plot comparing coverage uniformity between whole genome sequencing and adaptive sampling across centromeric regions. The coefficient of variation (SD/mean) for whole genome sequencing (y-axis) is broadly distributed, whereas the coefficient of variation for adaptive sampling (x-axis) is consistently low, demonstrating that adaptive sampling yields substantially more uniform coverage across centromeric arrays relative to whole genome sequencing. (B**)** Overlaid density histograms of mean sequencing coverage across 5 kb genomic bins for adaptive sampling (+AS) and whole genome sequencing (−AS). Coverage values were calculated by aggregating per-base depth into non-overlapping 5 kb windows. The +AS distribution is narrow and concentrated around the mean, reflecting highly uniform coverage across centromeric regions, whereas the −AS distribution is broadly dispersed, indicating greater bin-to-bin coverage variability in whole genome sequencing.

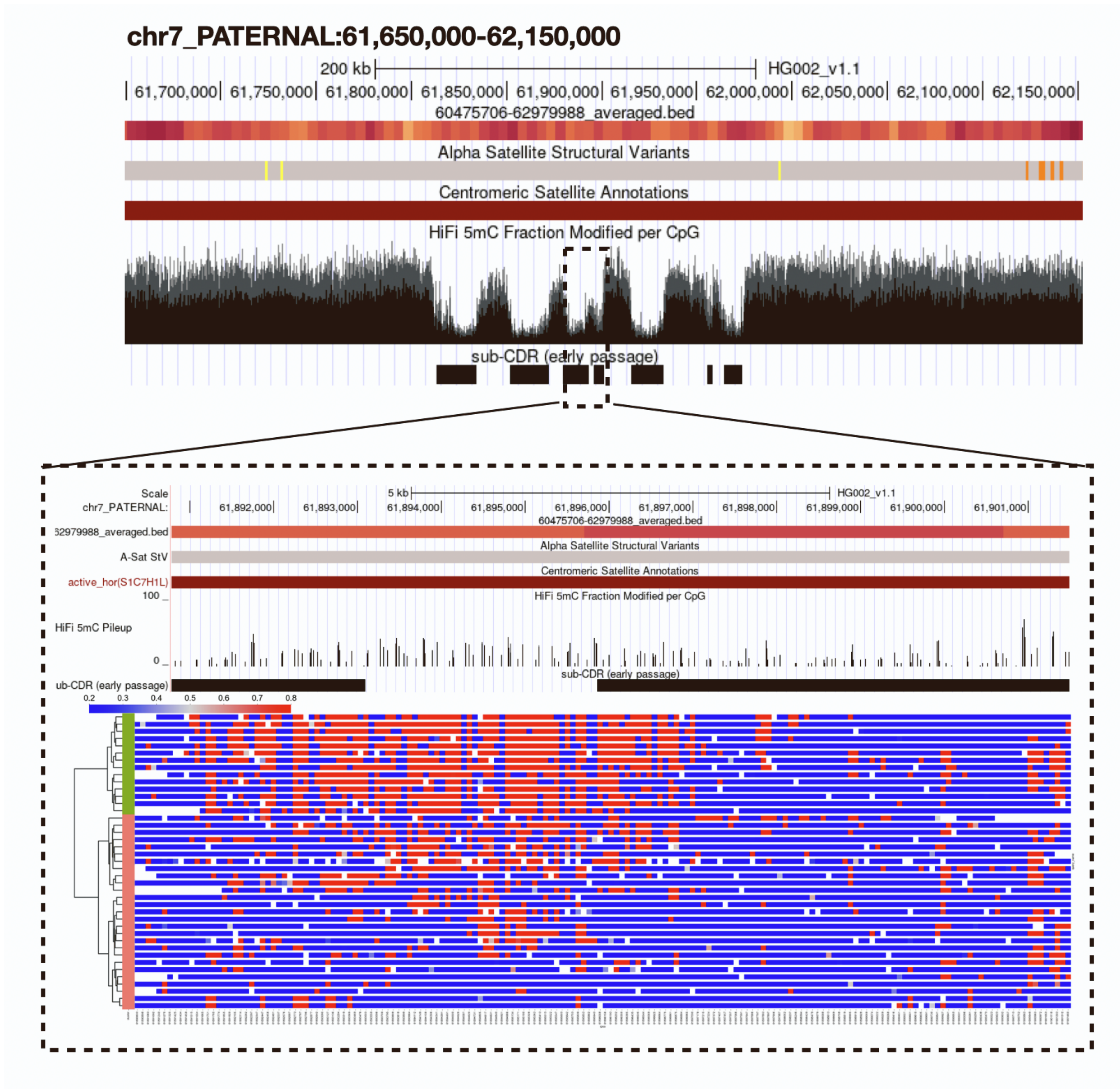

Figure S13: Clustering methylation data into groups, related to Figure 2

Clustered read matrices showing CpG methylation (5mC) signal across individual reads at the CDR edge in chromosome 7 paternal. Each row represents a single sequencing read, and each column a CpG position within the region. Reads were clustered using HDBSCAN based on their per-CpG 5mC probability profiles, with only reads covering at least 95% of the locus retained (windowFrac = 0.95). Distinct clusters reflect discrete molecule-level methylation states, supporting the interpretation that epigenetic heterogeneity at these regions is a genuine biological signal.

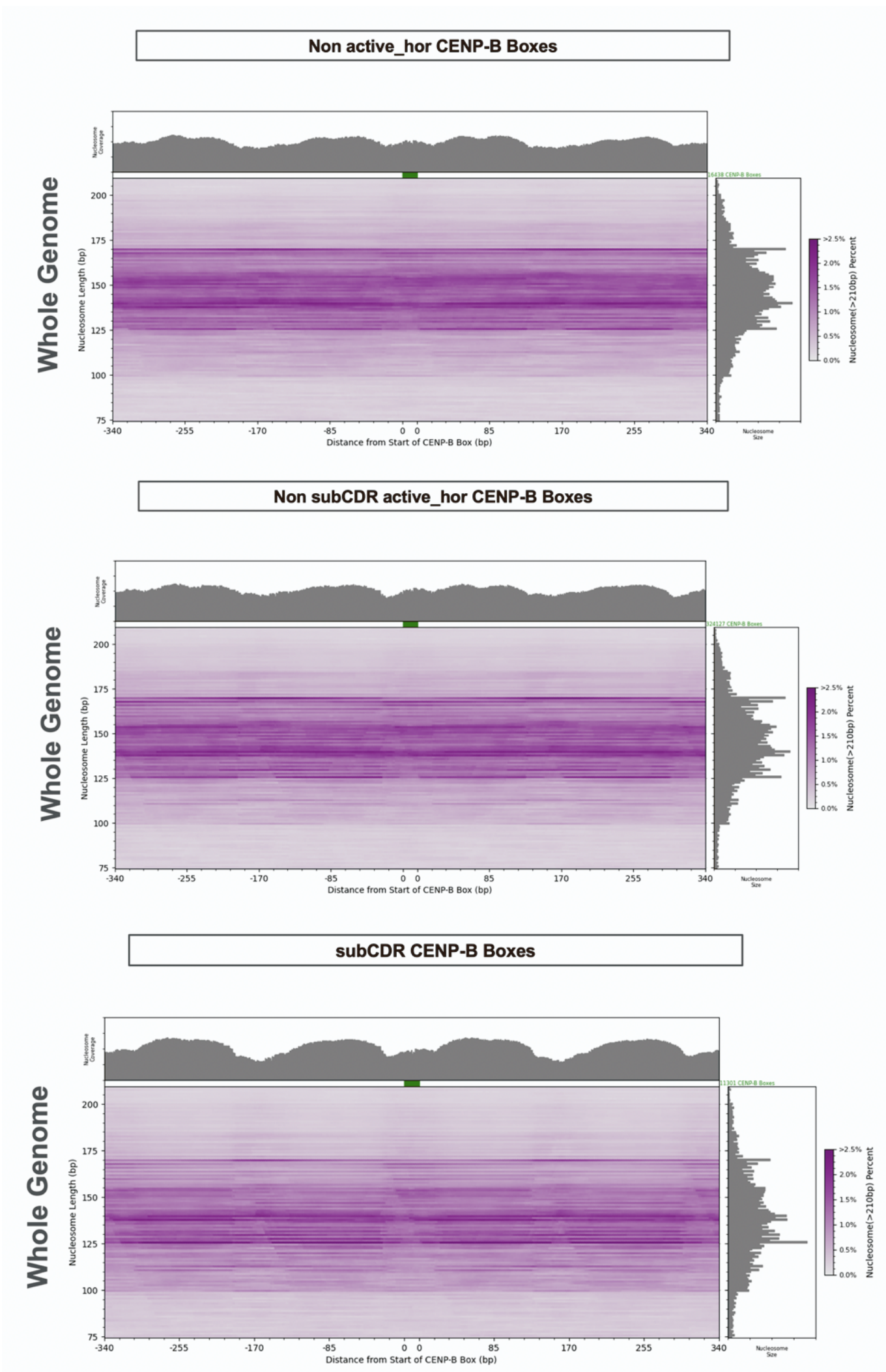

Figure S14: Fiber-seq nucleosome organization relative to CENP-B motifs, related to Figure 2
Fiber-seq[3] comparative nucleosome organization relative to centromere protein B (CENP-B) 17-bp motifs[4] in CDR, Active, and Inactive Alpha Arrays. Nucleosome positions with respect to reference coordinates were extracted using fibertools [5] extract. For each of the three regions (within subCDRs, non-CDR active alpha array, and inactive alpha array), a matrix was filled with the position of the nearest CENP-B motif and nucleosome-size. The matrix was then column-normalized to estimate the percentage of nucleosomes at specific sizes per distance from a CENP-B motif. Darker heatmap regions indicate a higher density of nucleosomes at a given size and position. Count totals are projected onto each axis as marginal histograms, summarizing the row and column distributions of the matrix. The top histogram summarizes nucleosome positional distribution relative to the CENP-B box motif, while the right histogram shows the overall nucleosome size distribution within each of the three regions observed.

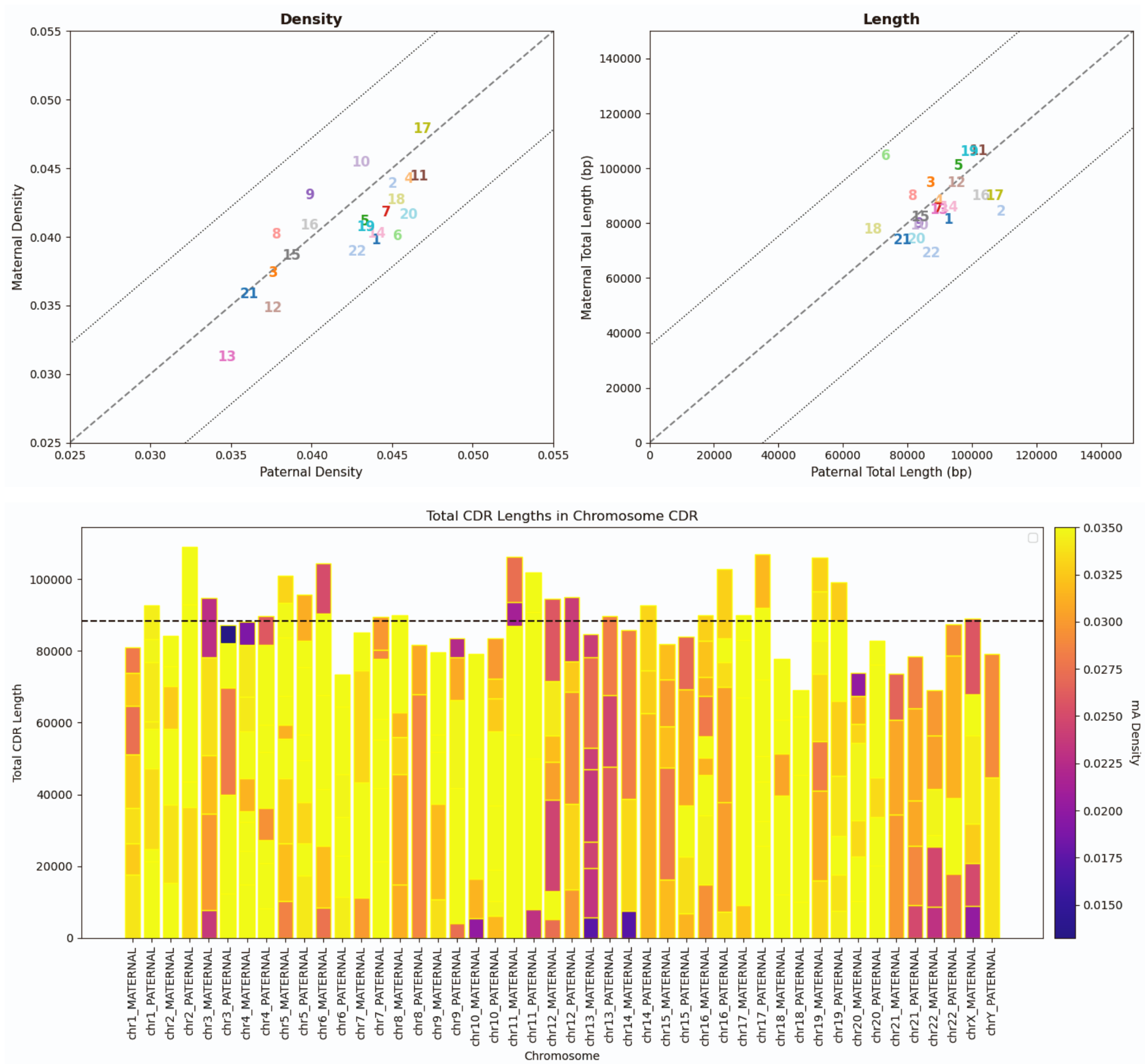

Figure S15: CENP-C DiMeLo-seq 6mA density (early passage), related to Figure 2
Centromere protein C (CENP-C) chromatin dosage is balanced between homologous centromeres in early-passage LCLs. (A) Scatter plots comparing paternal (x-axis) and maternal (y-axis) CENP-C DiMeLo-seq[6] 6mA signal density (left) and total sub-CDR length (right) across all chromosomes in early-passage HG002 LCLs. Each chromosome is labeled and color-coded. The dashed diagonal line represents the identity line; dotted lines indicate ±3 SD outlier thresholds. Chromosomes deviating beyond these thresholds represent haplotype-specific departures from dosage balance. (B) Stacked bar plots showing total CDR length per chromosome for maternal and paternal haplotypes for CENP-C DiMeLo-seq. Each bar is subdivided into individual sub-CDR segments colored by 6mA signal density using a plasma colormap. The dashed horizontal line indicates the mean total CDR length across all homologs. A color bar indicates the range of 6mA density values. Maternal and paternal haplotypes are displayed as adjacent pairs for each chromosome, ordered from chr1 to chrY.

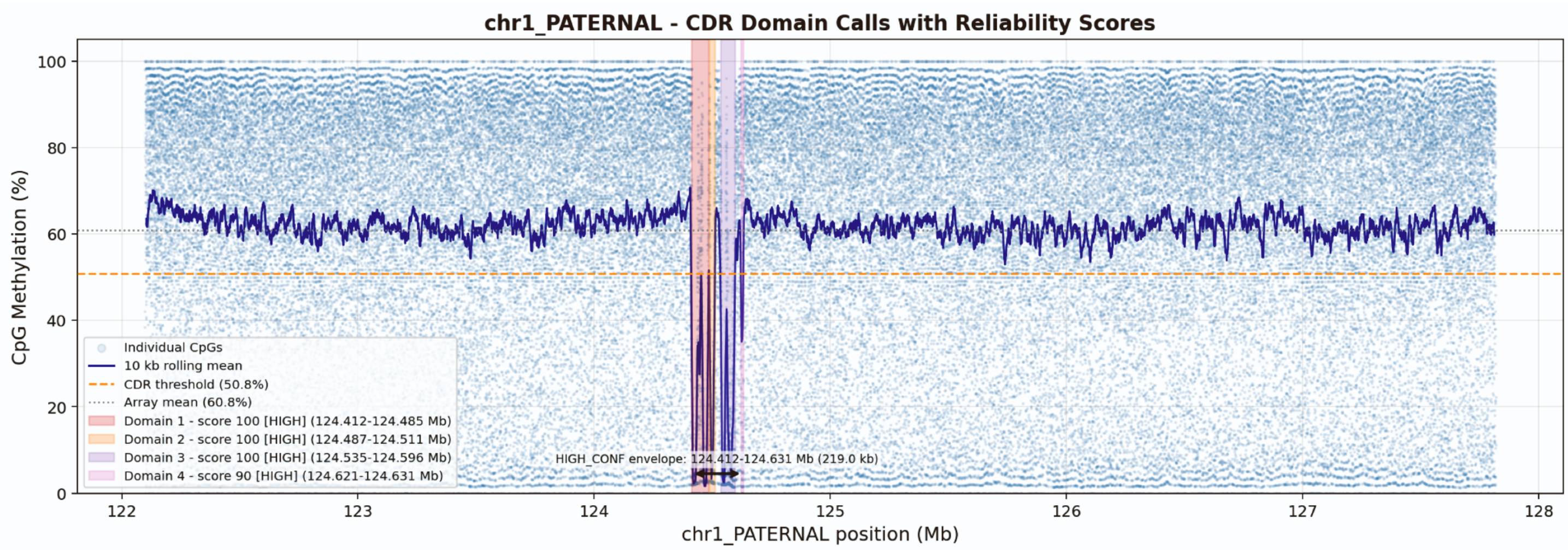

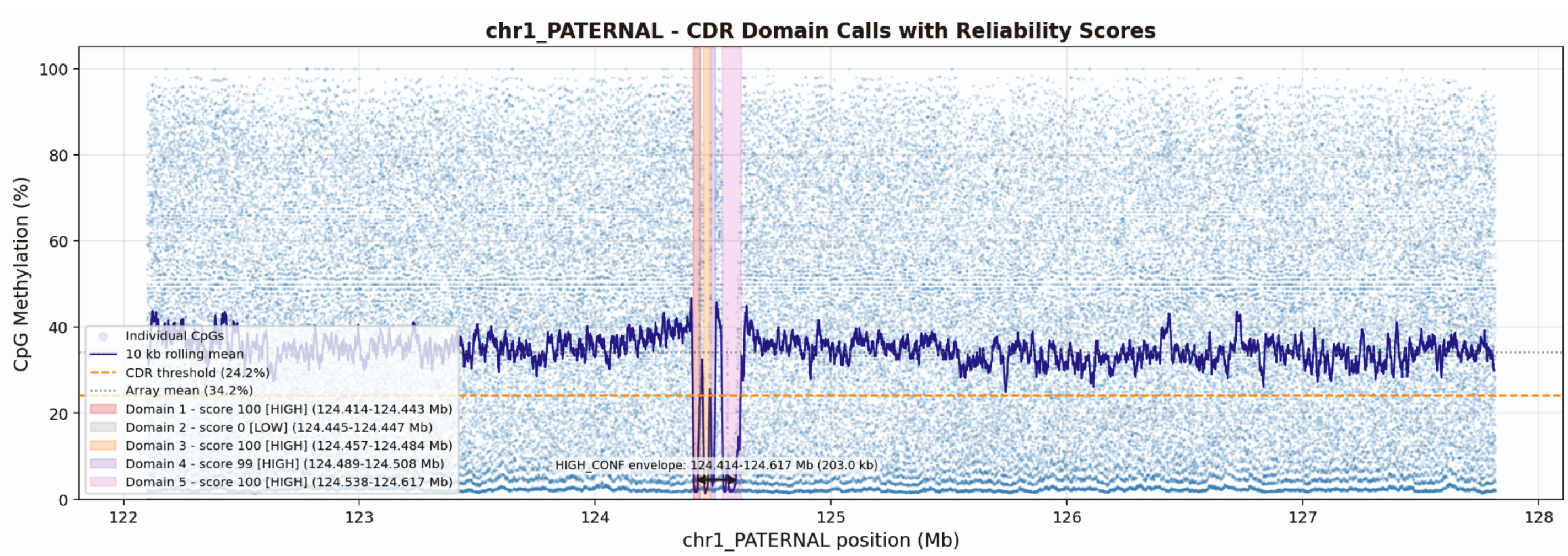

Figure S16: Methylation frequency across active arrays in early vs late passage, related to Figure 3

Methylation plot pileup (specifically BEDmethyl/bedGraph formats) summarizes per-base DNA methylation frequencies (e.g., 5mC) from CENP-A DiMeLo-seq data across the active array of chromosome 1_PATERNAL early passage (EP) (top) and late passage (LP) (bottom). Individual windows of CpG are provided in light blue, with a 10kb rolling mean in dark blue. The dotted line indicates the array mean (as shown for chromosome 1_PATERNAL, EP=60.8%, and LP=34.2%). CDRs are highlighted as individual domains that pass the threshold (10% difference from the array mean), and are shown to highlight that the CDRs remain in the same span, but have altered sub-CDR boundaries.

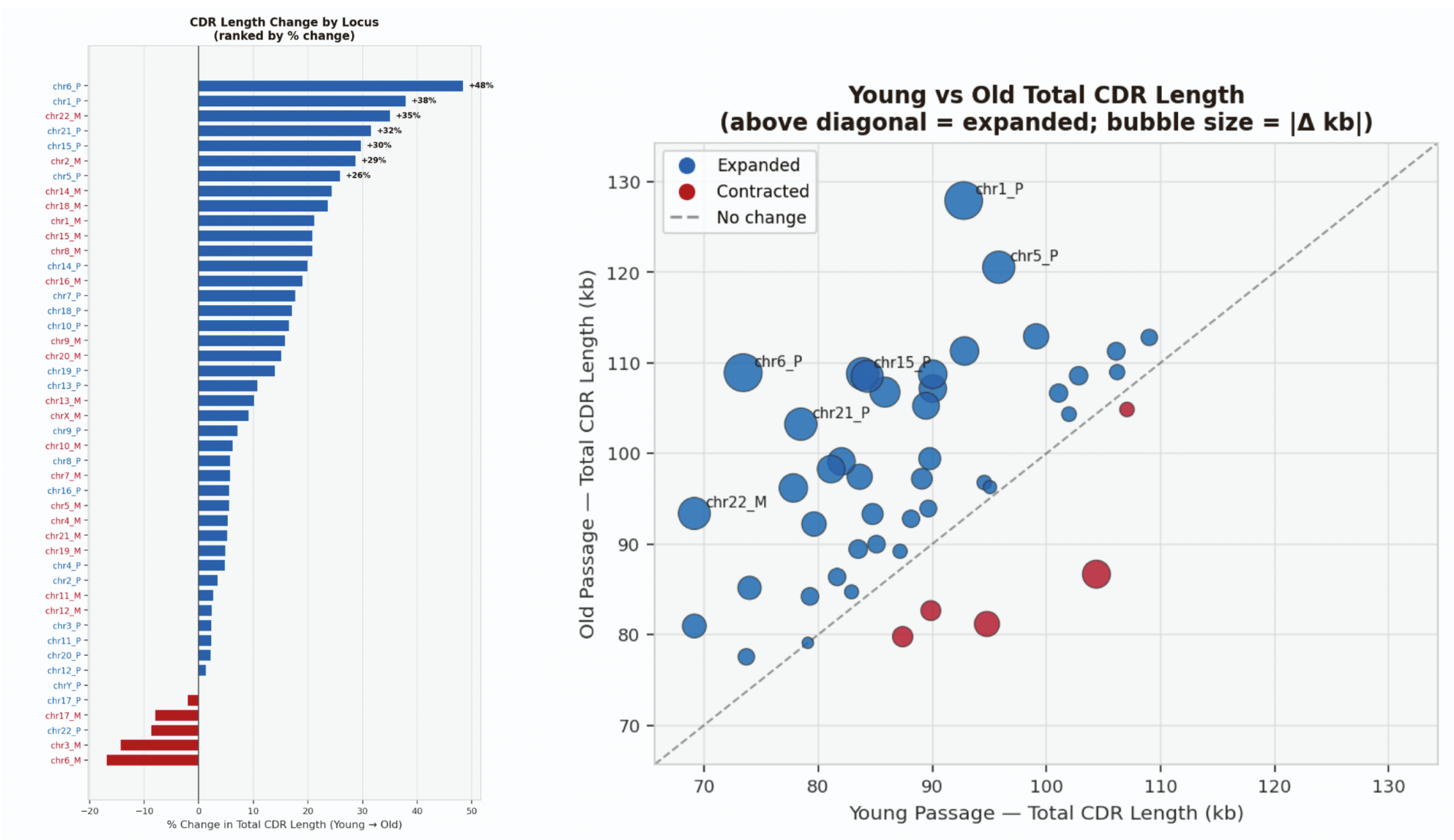

Figure S17: Difference in sub-CDR structure in early vs late passage, related to Figure 3

(Left) Ranked percent change in total sub-CDR aggregate length between early and late passage, shown for all active αSat arrays. Arrays are rank-ordered by magnitude of change, with increases shown in blue and decreases in red. Maternal and paternal haplotypes are labeled in red and blue, respectively. (Right) Scatter plot comparing total sub-CDR aggregate length (kb) between early (x-axis) and late (y-axis) passage. Each point represents a single array, with circle size proportional to the absolute magnitude of change between passages. The dashed line indicates the expectation under no change.

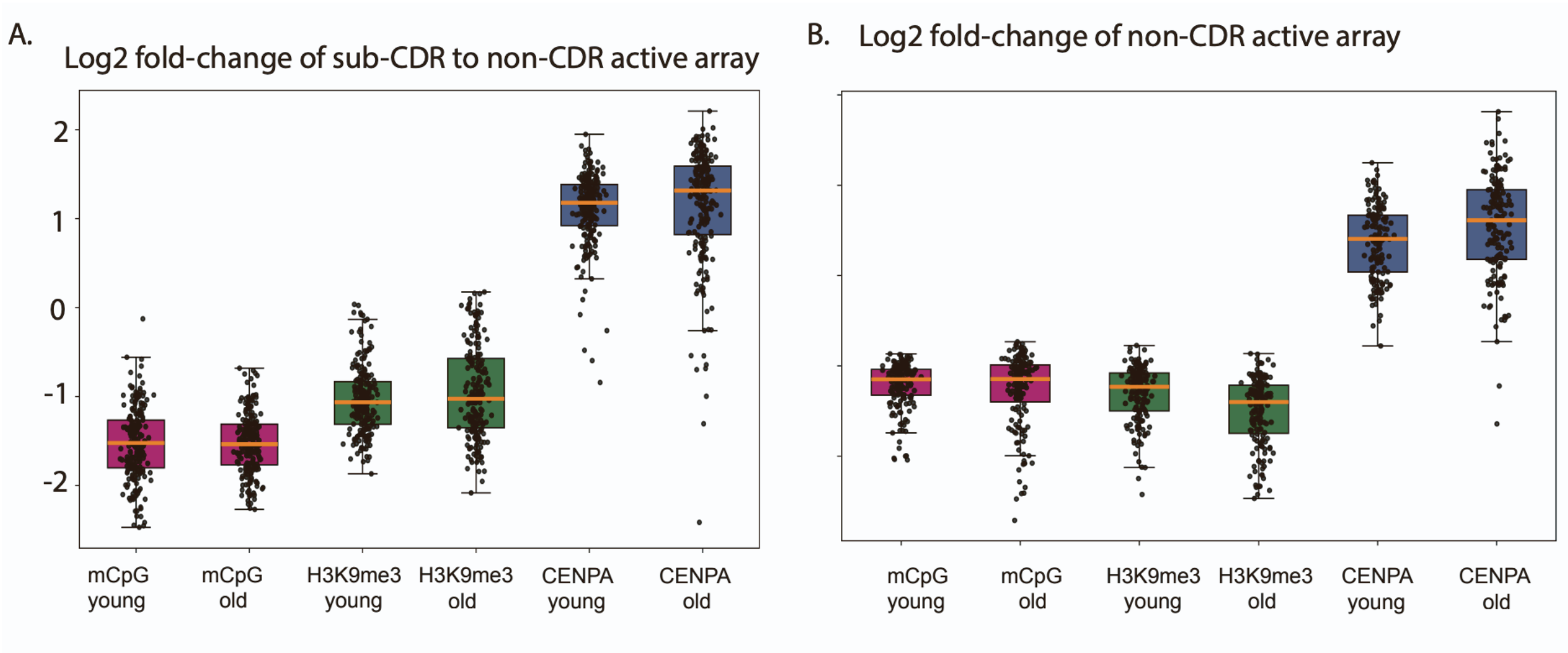

Figure S18: Enrichment within sub-CDRs across early- and late-passage, related to Figure 3 Epigenetic marks within general CDRs are stable between early- and late-passage LCLs. Box plots showing log2 fold-change of mCpG, H3K9me3, and CENP-A signal densities within sub-CDRs in early- and late-passage HG002 LCLs, calculated relative to chromosome-specific non-CDR active array baselines. Across all three marks, early- and late-passage cells show highly similar enrichment profiles within sub-CDRs: mCpG is comparably depleted in both conditions (median log2 fold-change ~−1.5), H3K9me3 is similarly reduced, and CENP-A is equivalently enriched, indicating that the relative epigenetic identity of the overall CDR domains is robustly maintained despite extended culture.

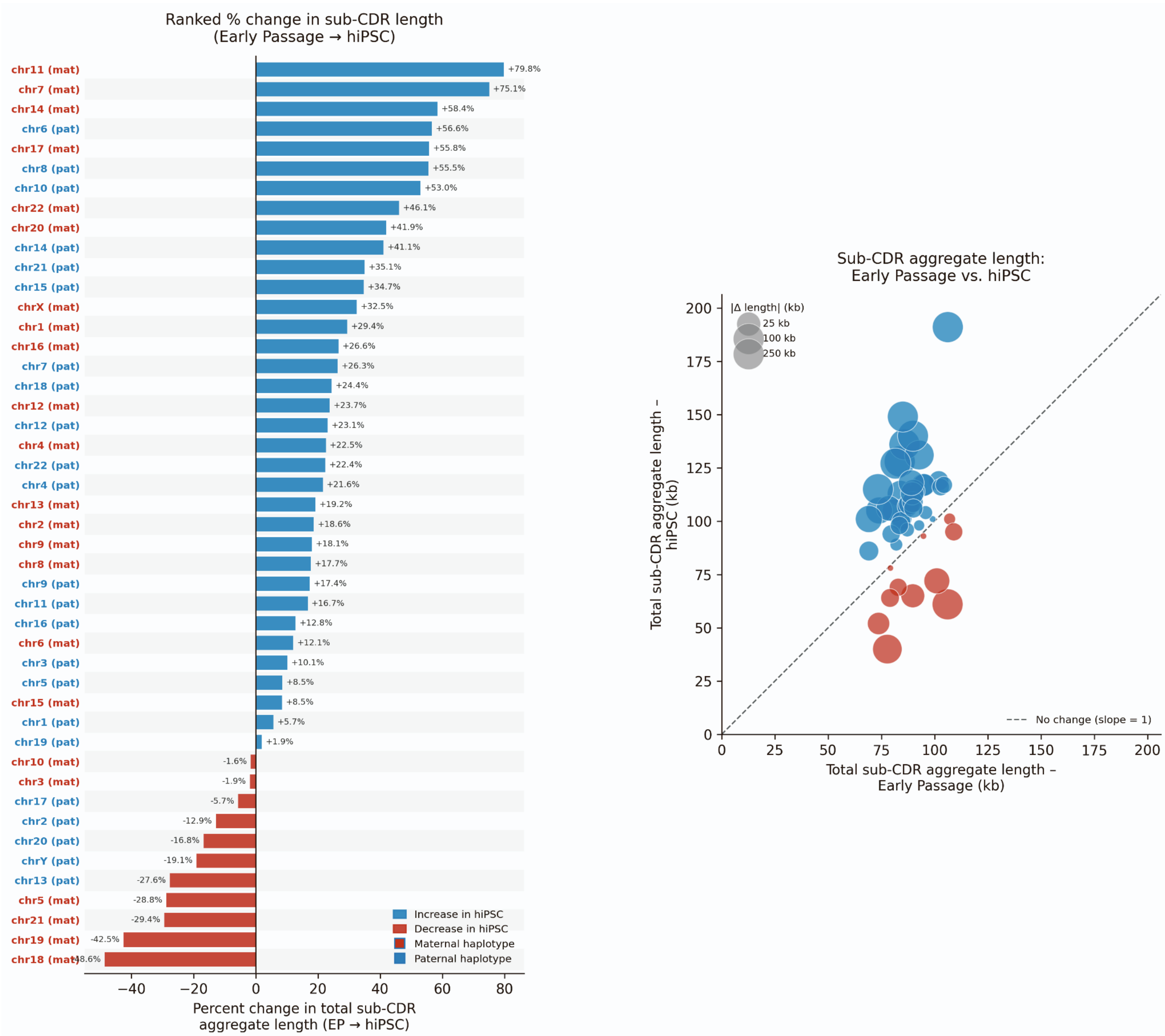

Figure S19: Comparison between HG002 early passage and hiPSC CDRs, related to Figure 3

(Left) Ranked percent change in total sub-CDR aggregate length between early passage (EP) and hiPSC, shown for all active αSat arrays across 46 haplotypes. Arrays are rank-ordered by magnitude of change; increases in hiPSC relative to EP are shown in blue and decreases in red. Maternal and paternal haplotypes are indicated by red and blue labels, respectively. (Right) Scatter plot comparing total sub-CDR aggregate length (kb) between EP (x-axis) and hiPSC (y-axis). Each point represents a single array; circle size is proportional to the absolute magnitude of change between conditions. The dashed line indicates the expectation under no change.

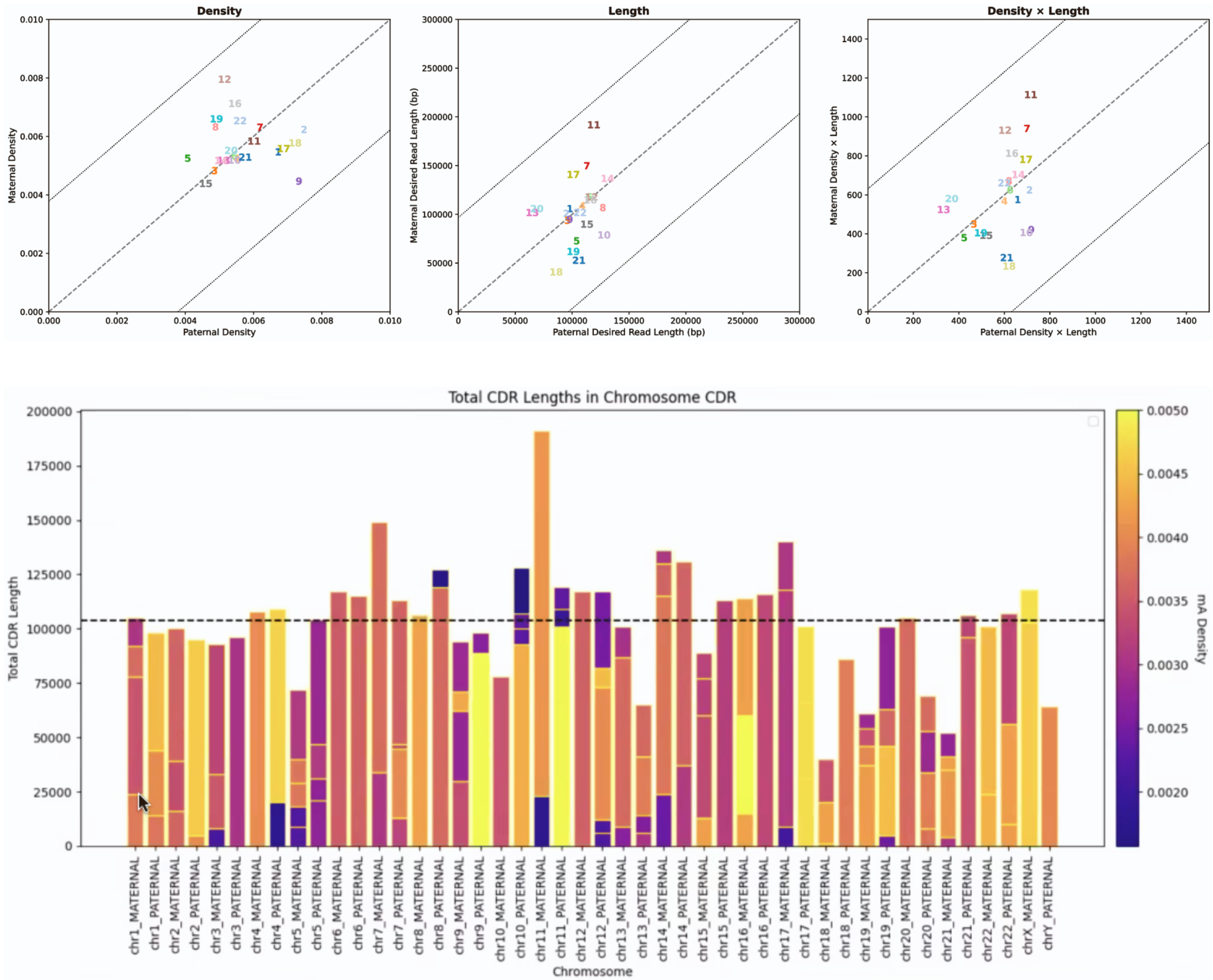

Figure S20: CENP-A DiMeLo-seq Density in hiPSCs, related to Figure 3

CENP-A chromatin dosage is balanced between homologous centromeres in PBMC-derived iPSCs. (A) Scatter plots comparing paternal (x-axis) and maternal (y-axis) CENP-A DiMeLo-seq 6mA signal density (left) and total sub-CDR length (right) across all chromosomes in PBMC-derived iPSCs. Each chromosome is labeled and color-coded. The dashed diagonal line represents the identity line; dotted lines indicate ±3 SD outlier thresholds. (B) Stacked bar plots showing total CDR length per chromosome for maternal and paternal haplotypes for late-passaged CENP-A DiMeLo-seq. Each bar is subdivided into individual sub-CDR segments colored by 6mA signal density using a plasma colormap. The dashed horizontal line indicates the mean total CDR length across all homologs. A color bar indicates the range of 6mA density values. Maternal and paternal haplotypes are displayed as adjacent pairs for each chromosome, ordered from chr1 to chrY.

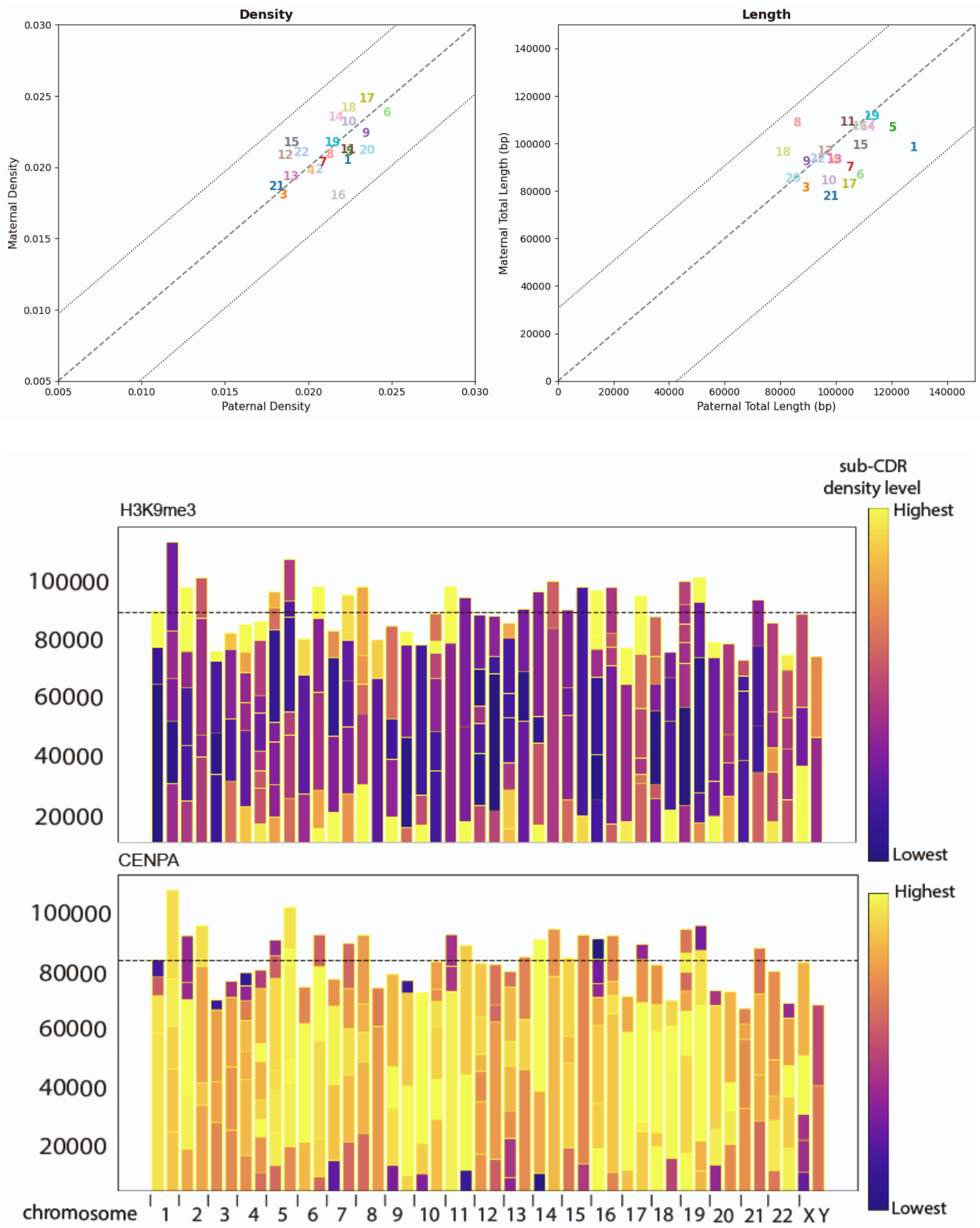

Figure S21: CENP-A DiMeLo-seq density in late passage lines, related to Figure 3

CENP-A chromatin dosage is balanced between homologous centromeres in late-passage LCLs. (A) Scatter plots comparing paternal (x-axis) and maternal (y-axis) CENP-A DiMeLo-seq 6mA signal density (left) and total sub-CDR length (right) across all chromosomes in late-passage HG002 LCLs. Each chromosome is labeled and color-coded. The dashed diagonal line represents the identity line; dotted lines indicate ±3 SD outlier thresholds. Chromosomes deviating beyond these thresholds represent haplotype-specific departures from dosage balance. (B) Stacked bar plots showing total CDR length per chromosome for maternal and paternal haplotypes for late-passaged CENP-A DiMeLo-seq. Each bar is subdivided into individual sub-CDR segments colored by 6mA signal density using a plasma colormap. The dashed horizontal line indicates the mean total CDR length across all homologs. A color bar indicates the range of 6mA density values. Maternal and paternal haplotypes are displayed as adjacent pairs for each chromosome, ordered from chr1 to chrY.

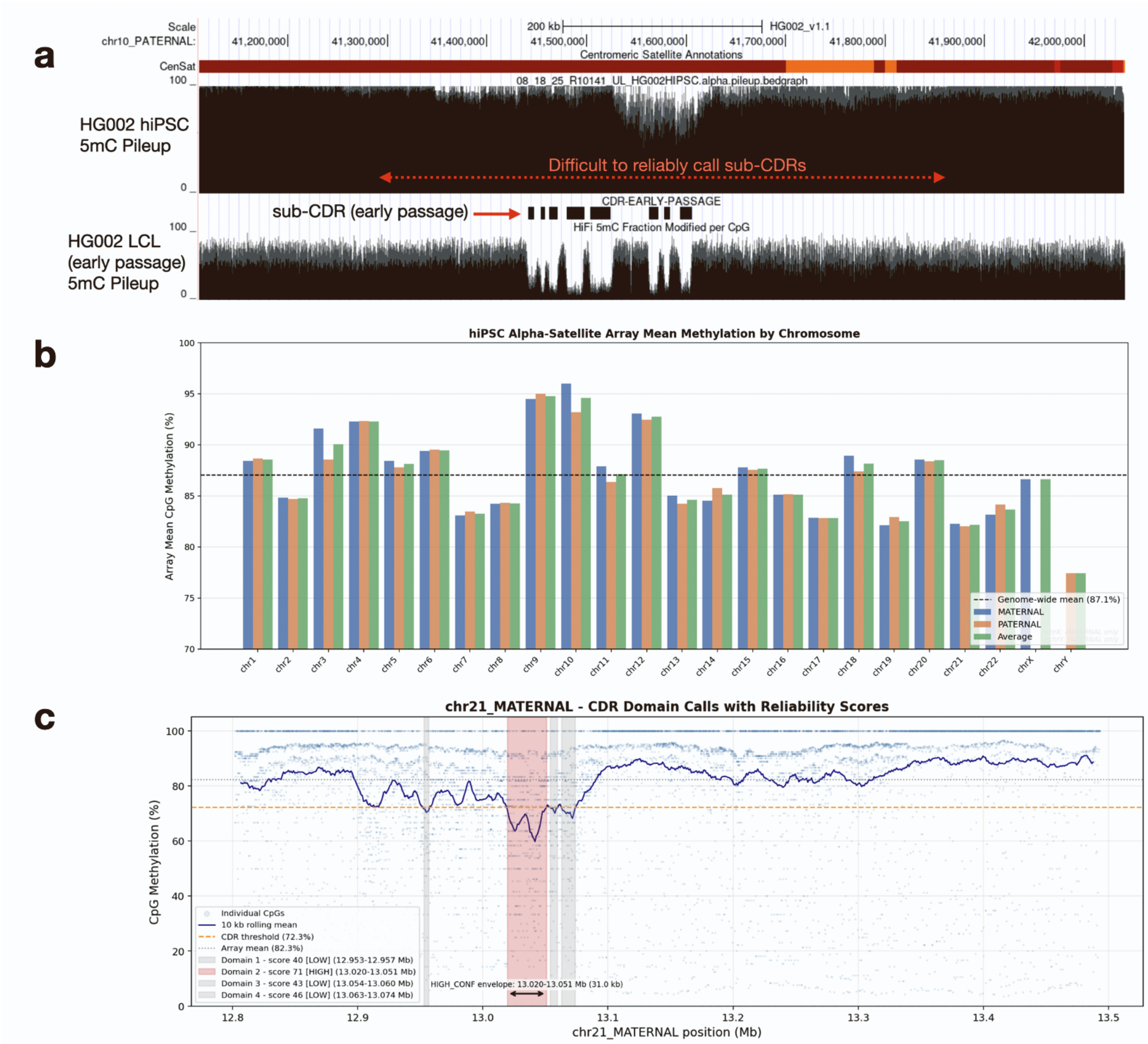

Figure S22: hiPSC array CpG density and CDR prediction, related to Figure 3.
Centromere dip regions (CDRs) are characterized by reduced CpG methylation relative to the surrounding active α-satellite higher-order repeat (HOR) array. HG002 hiPSC active arrays exhibit higher array-wide methylation than matched lymphoblastoid cell line (LCL) arrays (mean, 87% versus ~60%), complicating identification of sub-CDR boundaries. **(a)** UCSC Genome Browser visualization of the chromosome 10 paternal array illustrates the shallow methylation dip in hiPSCs (top) relative to early-passage cells (bottom), for which sub-CDR boundaries are difficult to define confidently. **(b)** For each haplotype-resolved α-satellite array, mean CpG methylation was calculated across all CpG sites and used to establish an array-specific baseline. The CDR detection threshold was defined as 10 percentage points below the array-wide mean methylation (CDR threshold = array mean methylation − 10%). To identify sub-CDRs in the hiPSC 5mC dataset, we developed a sliding-window CDR caller, illustrated for the chromosome 21 maternal array **(c)**. The caller identifies contiguous regions below the array-specific methylation threshold as distinct candidate domains and assigns each domain a composite reliability score to distinguish well-supported calls from marginal detections near the detection boundary. CDR calling was performed using phased, per-CpG methylation bedGraph files derived from Oxford Nanopore long-read sequencing and spanning the full assembled α-satellite array for each haplotype.

References

1. Raney, B.J., Dreszer, T.R., Barber, G.P., Clawson, H., Fujita, P.A., Wang, T., Nguyen, N., Paten, B., Zweig, A.S., Karolchik, D., et al. (2014). Track data hubs enable visualization of user-defined genome-wide annotations on the UCSC Genome Browser. Bioinformatics 30, 1003–1005. https://doi.org/10.1093/bioinformatics/btt637.

2. Sweeten, A.P., Schatz, M.C., and Phillippy, A.M. (2024). ModDotPlot—rapid and interactive visualization of tandem repeats. Bioinformatics 40, btae493. https://doi.org/10.1093/bioinformatics/btae493.

3. Stergachis, A.B., Debo, B.M., Haugen, E., Churchman, L.S., and Stamatoyannopoulos, J.A. (2020). Single-molecule regulatory architectures captured by chromatin fiber sequencing. Science 368, 1449–1454. https://doi.org/10.1126/science.aaz1646.

4. Muro, Y., Masumoto, H., Yoda, K., Nozaki, N., Ohashi, M., and Okazaki, T. (1992). Centromere protein B assembles human centromeric alpha-satellite DNA at the 17-bp sequence, CENP-B box. J. Cell Biol. 116, 585–596. https://doi.org/10.1083/jcb.116.3.585.

5. Jha, A., Bohaczuk, S.C., Mao, Y., Ranchalis, J., Mallory, B.J., Min, A.T., Hamm, M.O., Swanson, E., Dubocanin, D., Finkbeiner, C., et al. (2024). DNA-m6A calling and integrated long-read epigenetic and genetic analysis with fibertools. Genome Res. 34, 1976–1986. https://doi.org/10.1101/gr.279095.124.

6. Altemose, N., Maslan, A., Smith, O.K., Sundararajan, K., Brown, R.R., Mishra, R., Detweiler, A.M., Neff, N., Miga, K.H., Straight, A.F., et al. (2022). DiMeLo-seq: a long-read, single-molecule method for mapping protein-DNA interactions genome wide. Nat. Methods 19, 711–723. https://doi.org/10.1038/s41592-022-01475-6.
