# Supplementary material for: Haplotype-resolved DiMeLo-seq maps centromeric chromatin in a complete diploid human genome
Source: Cell Genom. 2026 Aug 6;6(8):101324. doi: 10.1016/j.xgen.2026.101324 (PMC13477032; doi:10.1016/j.xgen.2026.101324)
Supplement: Supplementary file 2 [file mmc12.pdf]

Cell Genomics Transparent Peer Review Record

Haplotype-resolved DiMeLo-seq maps centromeric chromatin in a complete diploid human genome

Author list: Yuan Xu, Hailey Loucks, Julian Menendez, Fedor Ryabov, Julian K. Lucas, Monika Cechova, Luke Morina, Emily Xu, Danilo Dubocanin, Cy Chittenden, Mobin Asri, Ivo Violich, Christian Ortiz, Joshua M.V. Gardner, Todd Hillaker, Sara O'Rourke, Brandy McNulty, Tamara A Potapova, Matthew W.Mitchell, Jacob P. Schwartz, Aaron F. Straight, Jennifer L. Gerton, Winston Timp, Ivan A.Alexandrov, Nicolas Altemose, Karen H. Miga

---

Summary

Initial submission:	Received : March 6th 2026
	Scientific editor: Judith Nicholson
First round of review:	Number of reviewers: 4<br>Revision invited : April 17th 2026<br>Revision received : May 18th 2026
Second round of review:	Number of reviewers: 3<br>Accepted : July 13th 2026
Data freely available:	Yes
Code freely available:	Yes

This transparent peer review record is not systematically proofread, type-set, or edited. Special characters, formatting, and equations may fail to render properly. Standard procedural text within the editor's letters has been deleted for the sake of brevity, but all official correspondence specific to the manuscript has been preserved.

Referees' reports, first round of review

Reviewer 1:

Review of Xu et al "Haplotype-resolved centromeric chromatin organization from a complete diploid human genome"

In this study, the authors employed the newly available complete phased diploid human genome T2T-HG002 to conduct an in-depth analysis of centromeric satellite DNA. By integrating this data with ultra-long-read, single-molecule chromatin profiling, they have effectively defined the organization of centromeric chromatin. Detailed annotation of centromeric satellites reveals extensive structural and evolutionary diversity in human centromeres. Intriguingly, despite this variability, the CDR (hypomethylated centromere dip region), composed of 2-9 sub-CDR's, maintains a remarkably consistent length. CENP-A targeted DiMeLo-seq confirms CENP-A localisation to CDR's and strong anti-correlation with DNA methylation. By modulating DNA methylation state with concomitant changes in CDR and CENP-A the authors suggest that "DNA methylation acts as a principal regulator of human centromere organisation".

This study presents a comprehensive analysis of centromeric DNA using cutting-edge, single-molecule long-read techniques. It offers detailed insights and intriguing findings that have the potential to advance our understanding of centromere chromatin structure. The high level of detail and innovative methodology make this work a valuable addition to the field and worthy of publication in Cell Genomics.

Minor comments:

* The relationship between DNA methylation and CDR/CENP-A domain is intriguing. However, why CDR's are unmethylated is still unclear. Have the authors mapped RNA pol II or transcription in HG002 cells?

* DNA methylation loss was accompanied by sub-CDR loss at the CDR boundary, could the authors comment on why this effect might be boundary specific?

* To look at DNA methylation loss the authors used late passage HG002 LCLs.

Could the authors comment on whether there is any change in DNA sequence at late passage?

* Supplementary Figure 12B: The data shown does not match the legend text.
* Supplementary Figure 16 is not mentioned in the main text.

Reviewer 2:

Centromeres are essential chromosome regions using for segregation during cell division. The underlying DNA sequences may be highly repetitive, as in the case of the human genome, where centromeres are constituted from megabase arrays of satellite repeats.

This study examines centromeric chromatin using a complete phased human genome T2T-HG002, together with epigenetic data from cell lines matched to this reference, including a lymphoblastoid and iPSC cell lines, and others. Exact matching of a high quality reference with the experimental data is a strength of the study, enabling precise maps of centromere chromatin to be obtained.

The authors report a custom annotation of tandem repeats including alpha and HSat satellite families in T2T-HG002. The authors present analysis revealing extensive structural polymorphism of the centromere regions between the two assembly haplotypes. The authors further map higher order patterns in the alpha satellites using their custom approaches.

DNA methylation analysis was performed to identify the CDR in each case, which correlated with regions of highest alpha satellite homogeneity and HOR structure.

The authors present very exciting DiMeLo-seq data that allow precise positioning of CENP-A nucleosomes in relation to DNA methylation. This was performed with ultra long ONT reads, providing power to phase the reads into the two haplotypes. To achieve sufficient depth of sequencing, adaptive sampling was also performed. Together this is an impressive technical achievement which has revealed new insights.

Fig 2B is wonderful! The sub-CDRs identified are quite large, 10s kb regions with

very low CG methylation, reduced H3K9me3, and enriched CENP-A (as revealed by deposition of methyl-A). Does this indicate that CG methylation is coupled to H3K9me3? ie more CENP-A means less H3 and less H3K9me3. If CG maintenance is coupled to H3K9me3 maintenance maybe that could explain this pattern?

However it is notable that the CG hypomethylation zones are very striking and 'cleaner' than H3K9me3 and CENP-A which appear a bit noiser in their distribution. Do the authors have thoughts on the different degrees of enrichment/de-enrichment in the subCDRs for these different modifications?

As there has been much recent work on B-boxes, I wondered if it might be worth promoting your B-box analysis in the supplement to one of the main figures, or further developing?

What do the authors believe determines subCDR locations? Could this reflect looping for example? Perhaps CiFi or Pore-C could be informative?

Out of curiosity, have the authors ever attempted to detect non-CG DNA methylation? It has been reported in some human cell types and tissues, but I don't recall a specific statement being made on it since complete centromere sequences have been generated. I believe this would be easy to definitely answer with these data and standard ONT models.

Minor points:

The abstract could be clearer and includes jargon that is hard to parse on first reading, eg 'bidirectional' is ambiguous, as is 'aggregate length'.

Fig 1C the end of the arrows pointing to the sub-CDRs are partially obscured by the figure panel.

Reviewer 3:

Centromere genomics have taken off since the complete sequencing of the centromere byt the T2T consortium. However critical questions remain

regarding the variability and heterogeneity of centromere sequence and epigenetic markers embedded within those sequences. The manuscript by Xu et al. take advantage of the T2T-HG002 assembly to examine the variability between maternal and paternal chromosomes and to determine the change that occur upon passage and differentiation examining HG002 iPSc and LCLs. Significant and important differences are observed between maternal and paternal centromere sequences, as well as differentiated and non-differentiated cell types. The authors detail differences in the flanking and central CDR's that could explain some aspects of centromere variability and movement. Overall, this is an important set of findings and provide a new and detailed examination of the variability in centromere sequences and epigenetics. I have a few suggestions for the manuscript.

1. Comparing IPSc that have been differentiated would be a better comparison than between the LCL and IPSC, given that LCLs show a fair amount of variability through continual passaging passage. The differences between LCL and IPSc may be due to the distinct culturing paths as opposed to a define IPSc vs LCL difference.

2. It would helpful if the main figure set includes a direct comparison between the maternal and paternal CDR locations and CENP-A locations. This could be accomplished either via select examples or a summary graph. The current graphs show size difference, but not location.

3. The data in figure 2F need to be quantified.

4. The reduced strength of mCpG and CENP-A patterns (Figure 3A) compared to the early passage could suggest that there is increased heterogeneity in these later stage cells. Can the author provide some analysis to assess whether this is the case.

5. The assessment of the early versus late stage demonstrates the trajectory of one cell line. An additional replicate would determine if the same trajectory of changes would occur, or if another cell lines may demonstrate a unqiue pattern of changes.

Reviewer 4:

The paper by Xu and colleagues presents a very interesting and detailed study describing centromeric sequence and chromatin architecture in a diploid, haplotype-resolved genome assembly from the HG002 benchmark sample of the Genome in a Bottle Consortium, for which well-characterized biological

resources, sequencing data, and broad donor consent for research are available. Characterizing the enigmatic centromeric loci in this genome is therefore highly relevant, both for advancing our understanding of human centromere biology and for establishing a complete genome and epigenome annotation of this model individual.

Using cutting-edge epigenomic profiling approaches, this work provides novel insights into the organization and heterogeneity of human centromeres, investigating variation in satellite array organization as well as the distribution of CENP-A and H3K9me3 domains together with methylation status at fine resolution. Changes in centromere organization during cell passaging or reprogramming were also detected. Beyond its biological significance, this study provides a comprehensive methodological framework for investigating human centromere biology and possibly for extending this type of approach to other species in the future.

The conclusions are well supported by the large amounts of data and the variety of methodological approaches. Although the manuscript is well written I suggest a few minor adjustments:

1) The size and organization of the CENP-A binding domain within satellite arrays, which could only recently be determined at high resolution thanks to advances in genome assembly and the development of novel epigenomic techniques, is consistent with the size and organization of centromeres devoid of satellite arrays previously identified in other mammalian species. Similarly, interindividual variation in the position of CENP-A binding domains, first discovered in satellite-free centromeres, can now be characterized in detail within satellite arrays. Since these findings reveal a consistent organization of centromeres independent of the presence of satellite DNA, I would recommend commenting on this parallelism in the Discussion.

2) The fact that the HG002 diploid genome is not simply haplotype-phased but that the maternal and paternal chromosomes can be identified and traced could be underlined in the Introduction.

3) Since the concept of active and inactive satellite arrays is fundamental for the understanding of the results, a precise definition of this concept should be

added at the beginning of the Results section or in the Introduction. Similarly, to improve clarity for the broad audience of Cell Genomics, the authors should explain what is known about younger and older HORs and their relationship to CENP-A binding.

4) Some of the Supplementary Tables (3, 5, 6, 7, 8) lack specification of the meaning of each column thus appearing as lists of numbers.

5) Although an explanation is detailed in Supplemental Note 1, the fact that grey regions correspond to deletions should be added to the legend of Supplementary Figure 7.

6) In the current pdf version, the figures are at low resolution. I imagine that this is related to the first-submission guidelines.

Authors' response to the first round of review

Dear Judith,

Thank you again for your editorial management of our manuscript, "Haplotype-resolved centromeric chromatin organization from a complete diploid human genome" (CELL-GENOMICS-D-26-00206), and for coordinating the peer review process. We are grateful for the positive reception of our work and are pleased to submit a revised version of the manuscript. We thank you and all four reviewers for their careful reading of our work and for their constructive feedback. The comments have helped us to strengthen the clarity, rigor, and presentation of our findings. We have also ensured that the revised manuscript complies with Cell Genomics formatting guidelines, including the STAR Methods format, the Key Resources Table, and data and code availability requirements.

The revisions address the four reviewers' principal concerns, which centered on the following areas: mechanistic questions regarding CDR hypomethylation and the relationship between CG methylation and H3K9me3; a request for a more direct comparison of maternal and paternal CDR and CENP-A locations in the main figures; additional analysis of the late-passage data; expansion of the Discussion to address parallels with satellite-free centromeres and to better define key concepts for a broad audience; and a range of figure and supplement corrections, including legend mismatches, missing table column headers, and figure clarity. Our detailed responses to each reviewer follow below (in **blue**).

Reviewers' Comments:

Reviewer #1: In this study, the authors employed the newly available complete phased diploid human genome T2T-HG002 to conduct an in-depth analysis of centromeric satellite DNA. By integrating this data with ultra-long-read, single-molecule chromatin profiling, they have effectively defined the organization of centromeric chromatin. Detailed annotation of centromeric satellites reveals extensive structural and evolutionary diversity in human centromeres. Intriguingly, despite this variability, the CDR (hypomethylated centromere dip region), composed of 2-9 sub-CDR's, maintains a remarkably consistent length. CENP-A targeted DiMeLo-seq confirms CENP-A localisation to CDR's and strong anti-correlation with DNA methylation. By modulating DNA methylation state with concomitant changes in CDR and CENP-A the authors suggest that "DNA methylation acts as a principal regulator of human centromere organisation". This study presents a comprehensive analysis of centromeric DNA using cutting-edge, single-molecule long-read techniques. It offers detailed insights and intriguing findings that have the potential to advance our understanding of centromere chromatin structure. The high level of detail and innovative methodology make this work a valuable addition to the field and worthy of publication in Cell Genomics.

We thank Reviewer #1 for their careful reading of our manuscript and for their generous and accurate summary of our work. We are pleased that the reviewer found the methodology innovative and the findings a valuable contribution to the field, and we appreciate their recommendation for publication in Cell Genomics.

Minor comments:

* The relationship between DNA methylation and CDR/CENP-A domain is intriguing. However, why CDR's are unmethylated is still unclear. Have the authors mapped RNA pol II or transcription in HG002 cells?

We thank the reviewer for raising this important point. The question of whether transcriptional activity

plays a role in CDR architecture is an intriguing one, and we agree it warrants attention. The current literature (Bobkov, *et al.* Journal of Cell Biology (2018)) does suggest that centromeric transcription, where it occurs, tends to be transient and low-level, making it exceptionally difficult to detect reliably, particularly within the highly repetitive sequences that define these regions. Short-read RNA sequencing approaches are not well-suited for this purpose, as reads cannot be mapped to these regions in a meaningful way, and any transcripts originating from CDR loci would likely fall below the detection threshold of standard methods. We have acknowledged this in the Discussion, noting that localized loss of DNA methylation at sub-CDRs may create a permissive chromatin environment for transient transcriptional activity, which has been proposed to facilitate CENP-A loading, and that rigorously testing this hypothesis will require more sophisticated RNA-based experimental approaches than are currently available for these highly repetitive regions. We consider this an important direction for future work.

* DNA methylation loss was accompanied by sub-CDR loss at the CDR boundary, could the authors comment on why this effect might be boundary specific?

We propose that the boundary-specific loss reflects a pre-existing asymmetry in CENP-A density across the CDR. The central sub-CDRs consistently show higher and more stable CENP-A occupancy, while the peripheral sub-CDRs at the CDR boundary exhibit lower and more variable CENP-A and H3K9me3 levels (Fig2D, Fig3C). This asymmetry is not a consequence of late-passage epigenetic changes; we also report detectable differences in early passage cells as well, suggesting that boundary sub-CDRs may be intrinsically less stable. We therefore propose that this lower and more variable chromatin occupancy at the periphery predisposes boundary sub-CDRs to loss when DNA methylation is perturbed at late passage.

* To look at DNA methylation loss the authors used late passage HG002 LCLs. Could the authors comment on whether there is any change in DNA sequence at late passage

This is an important question, and one that has been highlighted by recent work demonstrating that CDR shifts can arise in the context of large structural rearrangements (Porubsky et al., Nature 2025). We agree that DNA sequence stability in our late-passage lines must be carefully considered when interpreting the changes in sub-CDR organization we observe, and we have addressed this at the level of structural variation using Flagger, the quality assessment framework developed and applied by the T2T consortium and the Human Pangenome Reference Consortium as a best-in-class standard for evaluating centromeric array integrity. Remapping 28X coverage (N50 = 58 kb) reads, our Flagger analysis revealed no meaningful structural variation between early and late passage cells: the proportion of regions with expected haploid coverage was 93.9% in early passage and 91.8% in late passage, error-flagged regions increased modestly from 2.4% to 4.3%, and collapsed and duplicated regions remained effectively unchanged (Col: 3.2% vs. 3.2%; Dup: 0.5% vs. 0.6%). We note that confident SNV detection within centromeric repeats remains technically challenging due to sequencing error rates, somatic variation, and the absence of a validated truth set, and a rigorous SNV-level analysis is therefore beyond the scope of the current study. We agree that this is an important direction for future work as variant calling tools continue to mature for repetitive regions.

* Supplementary Figure 12B: The data shown does not match the legend text.

We thank the reviewer for catching this error. The panel in Supplementary Figure 12B has been replaced with the correct distribution chart for the legend text.

* Supplementary Figure 16 is not mentioned in the main text.

We thank the reviewer for bringing this to our attention. We have added a citation to Supplementary Figure 16 in the Results section, where we now report: "Total sub-CDR number decreased approximately two-fold (254 to 118), while mean CDR length increased 2.5-fold (16.0 kb to 40 kb), producing a strong shift toward large continuous domains with maximum sizes reaching ~168 kb (SFig 16)."

Reviewer #2: Centromeres are essential chromosome regions using for segregation during cell division. The underlying DNA sequences may be highly repetitive, as in the case of the human genome, where centromeres are constituted from megabase arrays of satellite repeats.

This study examines centromeric chromatin using a complete phased human genome T2T-HG002, together with epigenetic data from cell lines matched to this reference, including a lymphoblastoid and iPSC cell lines, and others. Exact matching of a high quality reference with the experimental data is a strength of the study, enabling precise maps of centromere chromatin to be obtained.

The authors report a custom annotation of tandem repeats including alpha and HSat satellite families in T2T-HG002. The authors present analysis revealing extensive structural polymorphism of the centromere regions between the two assembly haplotypes. The authors further map higher order patterns in the alpha satellites using their custom approaches.

DNA methylation analysis was performed to identify the CDR in each case, which correlated with regions of highest alpha satellite homogeneity and HOR structure.

The authors present very exciting DiMeLo-seq data that allow precise positioning of CENP-A nucleosomes in relation to DNA methylation. This was performed with ultra long ONT reads, providing power to phase the reads into the two haplotypes. To achieve sufficient depth of sequencing, adaptive sampling was also performed. Together this is an impressive technical achievement which has revealed new insights.

Fig 2B is wonderful! The sub-CDRs identified are quite large, 10s kb regions with very low CG methylation, reduced H3K9me3, and enriched CENP-A (as revealed by deposition of methyl-A). Does this indicate that CG methylation is coupled to H3K9me3? ie more CENP-A means less H3 and less H3K9me3. If CG maintenance is coupled to H3K9me3 maintenance maybe that could explain this pattern?

We thank the reviewer for this insightful mechanistic hypothesis. The model proposed that CENP-A enrichment displaces H3, reducing H3K9me3, which in turn impairs CG methylation maintenance, is biologically plausible. DNMT1 recruitment during replication requires UHRF1 recognition of H3K9me3, directly linking the two marks (Rothbart et al., 2012; Liu et al., 2013). The directionality of this coupling is important, however. To address whether the two marks are interdependent at centromeres, we point the reviewer to Salinas-Luypaert et al. (*Nature Genetics*, 2025), who employed a CENP-B DNA-binding domain fused to the TET1 catalytic domain to forcibly remove DNA methylation from the CDR and subsequently examined H3K9me3 levels. Strikingly, loss of mCpG at the CDR did not result in appreciable changes in H3K9me3, suggesting that these two marks are largely independent at centromeres on the time scale of that experiment (days). However, we note that this does not entirely preclude a coupling between CG methylation maintenance and H3K9me3 over longer time scales. The progressive and gradual loss of DNA methylation we observe, for example, across late passage cells, may reflect a slower dynamic that a targeted short-term demethylation experiment would not be designed to capture. Therefore, while the immediate maintenance of H3K9me3 appears independent of mCpG, a longer-term functional coupling between these marks at centromeres remains an open and important question.

However it is notable that the CG hypomethylation zones are very striking and 'cleaner' than H3K9me3 and CENP-A which appear a bit noiser in their distribution. Do the authors have thoughts on the different degrees of enrichment/de-enrichment in the subCDRs for these different modifications?

The difference in the sharpness of the boundaries between CG hypomethylation and the H3K9me3/CENP-A signals reflects fundamental technical differences in how these marks are measured.

DNA methylation (mCpG) is read directly from the sequencer as a native signal, yielding precise and sharp boundary definitions at single-nucleotide resolution. In contrast, CENP-A and H3K9me3 are measured indirectly via DiMeLo-seq, where an antibody-tethered methyltransferase deposits 6mA as a proxy for protein occupancy. This methyltransferase has a spread of up to ~1kb from the target protein binding site, and while the signal is concentrated within a few hundred bases of the true occupancy site, this inherent spatial diffusion will blur boundaries compared to the crisp readout of direct DNA methylation measurement. As a result, the apparent noisiness and less defined edges of the CENP-A and H3K9me3 distributions in the sub-CDRs are likely a technical feature of the DiMeLo-seq proximity labeling approach rather than a true biological difference in the precision of these chromatin domains.

As there has been much recent work on B-boxes, I wondered if it might be worth promoting your B-box analysis in the supplement to one of the main figures, or further developing?

We thank the reviewer for their enthusiasm for the B-box analysis. We agree that this is an exciting area of growing interest. However, we have chosen to keep this analysis in the supplement for the current manuscript, as we have a forthcoming paper examining B-boxes across a pangenome from 230 genomes (over 6000 complete T2T chromosomes), where this analysis will be substantially more

developed and better contextualized. We feel that further developing the B-box analysis here would be premature, given the more comprehensive treatment it will receive in that upcoming work.

What do the authors believe determines subCDR locations? Could this reflect looping for example? Perhaps CiFi or Pore-C could be informative?

While looping is an intriguing hypothesis, sub-CDR regions coincide with the most near-identical portions of the centromeric array, making high-confidence read mapping extremely challenging. Even with Pore-C, sub-reads are generally too short to map reliably within these repetitive regions, and it would be difficult to distinguish stable from transient looping structures. We consider this an exciting avenue for future investigation as long-read chromatin conformation technologies continue to mature.

Out of curiosity, have the authors ever attempted to detect non-CG DNA methylation? It has been reported in some human cell types and tissues, but I don't recall a specific statement being made on it since complete centromere sequences have been generated. I believe this would be easy to definitely answer with these data and standard ONT models.

We did not specifically examine non-CG DNA methylation in this study. While ONT's Dorado basecaller does include models that nominally support 5mC detection in all sequence contexts (including CHG and CHH), comprehensive benchmarking of these models demonstrates that their performance at non-CG sites is substantially inferior to CpG methylation detection, with markedly increased false positive rates. Furthermore, the available non-CG models have been trained and validated primarily on plant genomes, where non-CG methylation is abundant and biologically canonical. In human cells, where non-CG methylation is sparse and context-specific, validated models with sufficient accuracy for confident non-CG methylation calls at centromeric repetitive sequences do not currently exist. Critically, even if a signal were detected, we lack an orthogonal method to validate such findings, and because we performed adaptive sampling, we cannot confidently assess whether any putative signal represents true enrichment at centromeres relative to other genomic regions.

Minor points:

The abstract could be clearer and includes jargon that is hard to parse on first reading, eg 'bidirectional' is ambiguous, as is 'aggregate length'.

We thank the reviewer for this suggestion. We have revised the abstract to improve clarity: "aggregate length" has been replaced with "combined CDR span", and "bidirectional changes to centromeric and pericentromeric DNA methylation" has been replaced with "both gain and loss of centromeric and pericentromeric DNA methylation", which more explicitly conveys that we observe changes in both directions across distinct cell contexts.

Fig 1C the end of the arrows pointing to the sub-CDRs are partially obscured by the figure panel.

We appreciate this feedback and have revised Fig 1C to ensure the arrows and labels indicating sub-CDRs are fully visible and no longer obscured by the figure panel boundary

Reviewer #3: Centromere genomics have taken off since the complete sequencing of the centromere byt the T2T consortium. However critical questions remain regarding the variability and heterogeneity of centromere sequence and epigenetic markers embedded within those sequences. The manuscript by Xu et al. take advantage of the T2T-HG002 assembly to examine the variability between maternal and paternal chromosomes and to determine the change that occur upon passage and differentiation examining HG002 iPSc and LCLs. Significant and important differences are observed between maternal and paternal centromere sequences, as well as differentiated and non-differentiated cell types. The authors detail differences in the flanking and central CDR's that could explain some aspects of centromere variability and movement. Overall, this is an important set of findings and provide a new and detailed examination of the variability in centromere sequences and epigenetics. I have a few suggestions for the manuscript.

1. Comparing IPSc that have been differentiated would be a better comparison than between the LCL and IPSC, given that LCLs show a fair amount of variability through continual passaging passage. The differences between LCL and IPSc may be due to the distinct culturing paths as opposed to a define IPSc vs LCL difference.

We agree that same-lineage comparisons are more controlled. We point the reviewer to Dong et al. (2026; DOI: 10.64898/2026.02.14.705860), who profiled PBMCs reprogrammed into iPSCs and subsequently differentiated into neural progenitor cells (NPCs) across a three-generation pedigree. Consistent with our findings, they show that reprogramming markedly attenuates CDR hypomethylation by ~49% on average, with CDRs becoming substantially narrower in iPSCs (mean 306 kb in PBMCs vs. 217 kb in iPSCs), directly corroborating our observations of array hypermethylation and sub-CDR merging upon reprogramming. Importantly, CDR positions remain stable across these cell-fate transitions, and hypomethylation is partially restored upon differentiation into NPCs, further supporting the conclusion that the CDR differences we observe between LCLs and iPSCs reflect genuine cell-type-specific centromeric chromatin reorganization rather than artifacts of distinct culturing histories.

2. It would helpful if the main figure set includes a direct comparison between the maternal and paternal CDR locations and CENP-A locations. This could be accomplished either via select examples or a summary graph. The current graphs show size difference, but not location.

We point the reviewer to Figure 1A, which displays CDR locations across all annotated chromosomes for both haplotypes, allowing for visual comparison of maternal and paternal CDR positioning. We note that a direct quantitative comparison of CDR location between haplotypes is complicated by the fact that the maternal and paternal arrays are often substantially different in size and structure, making positional comparisons difficult to interpret in a meaningful way. However, we do discuss the tendency for CDRs to be positioned more proximal to the p-arm, which represents the clearest positional trend we can confidently report across haplotypes.

3. The data in figure 2F need to be quantified.

We have now added a y axis label to this panel.

4. The reduced strength of mCpG and CENP-A patterns (Figure 3A) compared to the early passage could suggest that there is increased heterogeneity in these later stage cells. Can the author provide some analysis to assess whether this is the case.

By examining single-molecule reads, we actually observe decreased heterogeneity in mCpG and CENP-A patterns in late-passage cells, with methylation at CDR boundaries appearing increasingly uniform (Supplementary Figure 13). This is consistent with a bottleneck effect in which extended passaging selectively enriches for a population of HG002 cells with a specific centromeric mCpG pattern.

5. The assessment of the early versus late stage demonstrates the trajectory of one cell line. An additional replicate would determine if the same trajectory of changes would occur, or if another cell lines may demonstrate a unqiue pattern of changes.

We note that Mahlke et al. (doi: https://doi.org/10.1101/2025.02.03.636285) examined centromere epigenetic evolution across multiple independent clones derived from the same parental cell line over prolonged proliferation, and found that while all clones underwent centromeric changes, each acquired a distinct pattern of CENP-A positional drift and CDR evolution. This supports the notion that the trajectory of centromeric changes is not strictly deterministic, but the general phenomenon of progressive epigenetic remodeling with extended passaging is a consistent feature. Our observations in late-passage HG002 LCLs are therefore consistent with this broader pattern, and we consider the examination of additional cell lines an important direction for future work.

Reviewer #4:

The paper by Xu and colleagues presents a very interesting and detailed study describing centromeric sequence and chromatin architecture in a diploid, haplotype-resolved genome assembly from the HG002 benchmark sample of the Genome in a Bottle Consortium, for which well-characterized biological resources, sequencing data, and broad donor consent for research are available. Characterizing the enigmatic centromeric loci in this genome is therefore highly relevant, both for advancing our understanding of human centromere biology and for establishing a complete genome and epigenome annotation of this model individual.

Using cutting-edge epigenomic profiling approaches, this work provides novel insights into the organization and heterogeneity of human centromeres, investigating variation in satellite array organization as well as the distribution of CENP-A and H3K9me3 domains together with methylation status at fine resolution. Changes in centromere organization during cell passaging or reprogramming were also detected. Beyond its biological significance, this study provides a comprehensive methodological framework for investigating human centromere biology and possibly for extending this type of approach to other species in the future.

The conclusions are well supported by the large amounts of data and the variety of methodological approaches. Although the manuscript is well written I suggest a few minor adjustments:

1) The size and organization of the CENP-A binding domain within satellite arrays, which could only recently be determined at high resolution thanks to advances in genome assembly and the development of novel epigenomic techniques, is consistent with the size and organization of centromeres devoid of satellite arrays

previously identified in other mammalian species. Similarly, interindividual variation in the position of CENP-A binding domains, first discovered in satellite-free centromeres, can now be characterized in detail within satellite arrays. Since these findings reveal a consistent organization of centromeres independent of the presence of satellite DNA, I would recommend commenting on this parallelism in the Discussion.

We thank the reviewer for this suggestion and agree that this parallelism is worth highlighting. We have added the following to the Discussion, noting the consistency between CENP-A domain size, organization, and positional variation observed here within alpha satellite arrays and those previously described at satellite-free centromeres in other mammalian species and at non-repetitive neocentromeres in humans.

2) The fact that the HG002 diploid genome is not simply haplotype-phased but that the maternal and paternal chromosomes can be identified and traced could be underlined in the Introduction.

We thank the reviewer for this suggestion. We have added a sentence to the Introduction clarifying that the maternal and paternal chromosomes of HG002 are fully identifiable and traceable, enabling direct comparison of centromere organization between homologs of known parental origin.

3) Since the concept of active and inactive satellite arrays is fundamental for the understanding of the results, a precise definition of this concept should be added at the beginning of the Results section or in the Introduction. Similarly, to improve clarity for the broad audience of Cell Genomics, the authors should explain what is known about younger and older HORs and their relationship to CENP-A binding.

We thank the reviewer for this suggestion. We have added a sentence to the third paragraph of the first Results section defining active and inactive satellite arrays, and have additionally clarified the relationship between younger and older HORs and their association with CENP-A binding to improve accessibility for a broad audience.

4) Some of the Supplementary Tables (3, 5, 6, 7, 8) lack specification of the meaning of each column thus appearing as lists of numbers.

We thank the reviewer for this observation. We have added column labels and descriptions to Supplementary Tables 3, 5, 6, 7, and 8 to clarify the meaning of each column.

5) Although an explanation is detailed in Supplemental Note 1, the fact that grey regions correspond to deletions should be added to the legend of Supplementary Figure 7.

We thank the reviewer for this suggestion. We have updated the legend of Supplementary Figure 7 to clarify that grey regions correspond to deletions.

6) In the current pdf version, the figures are at low resolution. I imagine that this is related to the first-submission guidelines.

We appreciate this comment and have uploaded high-resolution figures for consideration during the publication process.

We thank all four reviewers and the editorial team once more for their time and thoughtful engagement with our work. We believe the revisions have substantially strengthened the manuscript and hope that the revised version is suitable for publication in Cell Genomics.

Referees' report, second round of review

Reviewer 1:

Thank you for addressing my comments. I am satisfied with the revisions.

Reviewer 2:

Thank you, the authors have addressed my concerns. Ian Henderson

Reviewer 3:

The authors have address the concerns from the previous round of review. This is an important set of observations and I support publication of this manuscript in Cell Genomics.

Authors' response to the second round of review
