# Supplementary material for: Haplotype-resolved DiMeLo-seq maps centromeric chromatin in a complete diploid human genome
Source: Cell Genom. 2026 Aug 6;6(8):101324. doi: 10.1016/j.xgen.2026.101324 (PMC13477032; doi:10.1016/j.xgen.2026.101324)
Supplement: Supplementary file 3 [file mmc13.pdf]

Cell Genomics

Short article

Haplotype-resolved DiMeLo-seq maps centromeric chromatin in a complete diploid human genome

Graphical abstract

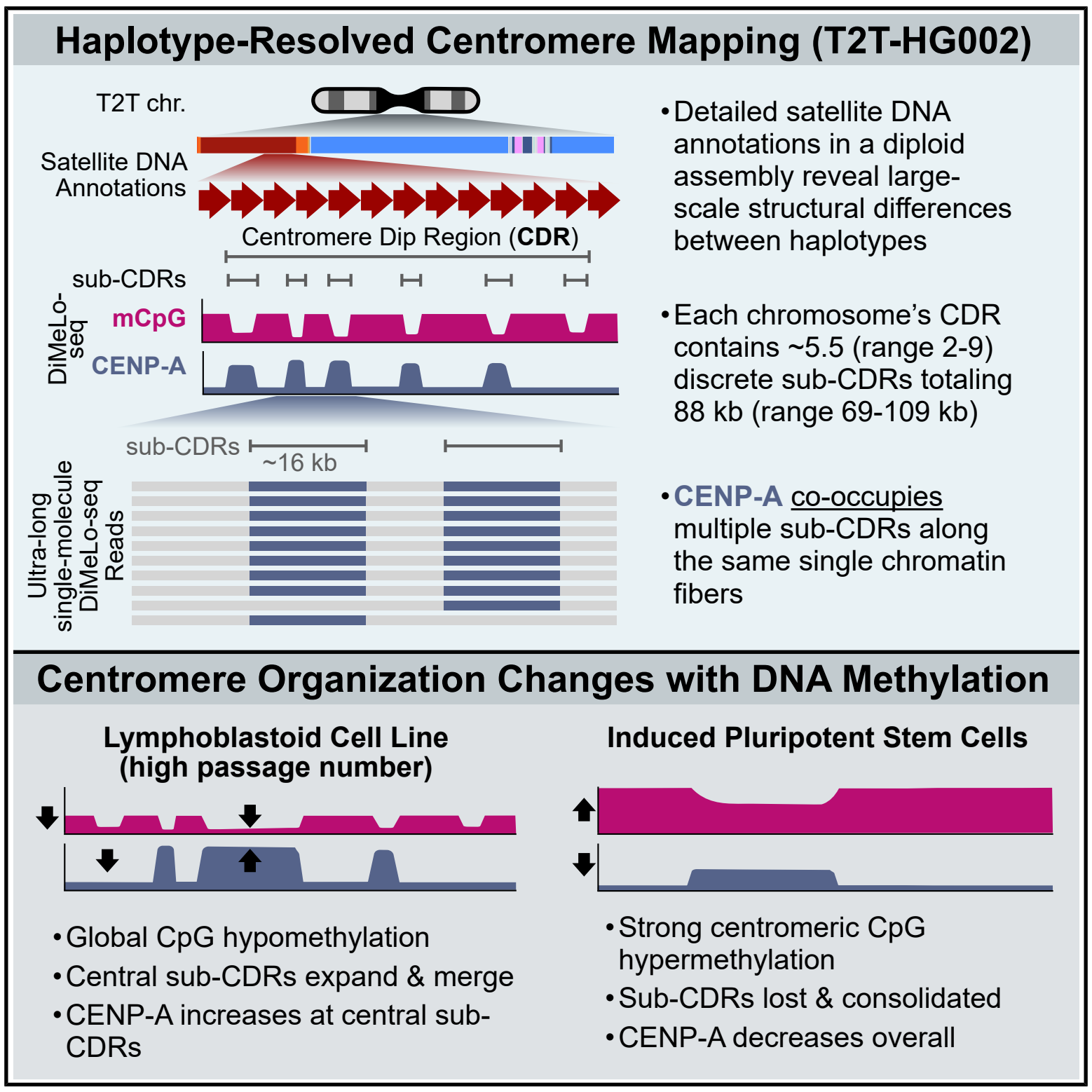

Highlights

- Ultra-long DiMeLo-seq maps CENP-A on both homologs of a diploid LCL genome
- CENP-A co-occupies ∼5.5 discrete hypomethylated subdomains (∼88 kb) per chromosome
- H3K9me3 is typically, but not always, excluded from hypomethylated subdomains
- CENP-A domain size and number change with centromeric DNA hypo- and hypermethylation

Authors

Yuan Xu, Hailey Loucks, Julian Menendez, ..., Ivan A. Alexandrov, Nicolas Altemose, Karen H. Miga

Correspondence

altemose@stanford.edu (N.A.),
khmiga@ucsc.edu (K.H.M.)

In brief

Centromeres direct accurate chromosome inheritance, but their chromatin organization has remained unclear. Xu et al. map the centromere-defining protein CENP-A across both copies of every chromosome in a complete human genome, showing that on each chromosome, CENP-A simultaneously occupies multiple discrete domains that strongly associate with DNA hypomethylation.

Xu et al., 2026, Cell Genomics *6*, 101324
August 12, 2026 © 2026 The Authors. Published by Elsevier Inc.
https://doi.org/10.1016/j.xgen.2026.101324

Cell Genomics

CellPress OPEN ACCESS

Short article

Haplotype-resolved DiMeLo-seq maps centromeric chromatin in a complete diploid human genome

Yuan Xu,[1] Hailey Loucks,[1] Julian Menendez,[1] Fedor Ryabov,[2,3,4] Julian K. Lucas,[1] Monika Cechova,[2,5] Luke Morina,[6] Emily Xu,[1] Danilo Dubocanin,[7] Cy Chittenden,[7] Mobin Asri,[1] Ivo Violich,[2] Christian Ortiz,[8] Joshua M.V. Gardner,[2] Todd Hillaker,[2] Sara O'Rourke,[1] Brandy McNulty,[2] Tamara A. Potapova,[9] Matthew W. Mitchell,[10] Jacob P. Schwartz,[11] Aaron F. Straight,[12] Jennifer L. Gerton,[9] Winston Timp,[6,13] Ivan A. Alexandrov,[14] Nicolas Altemose,[7,15,*] and Karen H. Miga[1,2,16,*]

[1]Department of Biomolecular Engineering, University of California, Santa Cruz, Santa Cruz, CA 95064, USA
[2]UC Santa Cruz Genomics Institute, University of California, Santa Cruz, Santa Cruz, CA 95064, USA
[3]Centre for Biomedical Research and Technology, HSE University, Moscow 101000, Russia
[4]The Center for Bio- and Medical Technologies, Moscow 121205, Russia
[5]Faculty of Informatics, Masaryk University, 601 77 Brno, Czech Republic
[6]Department of Biomedical Engineering, Johns Hopkins University, Baltimore, MD 21218, USA
[7]Department of Genetics, Stanford University School of Medicine, Stanford, CA 94305, USA
[8]Department of Molecular, Cell and Developmental Biology, University of California, Santa Cruz, Santa Cruz, CA 95064, USA
[9]Stowers Institute for Medical Research, Kansas City, MO 64110, USA
[10]Coriell Institute for Medical Research, Camden, NJ 08103, USA
[11]Department of Molecular and Cellular Physiology, Stanford University, Palo Alto, CA 94305, USA
[12]Department of Biochemistry, Stanford University, Palo Alto, CA 94305, USA
[13]Department of Molecular Biology and Genetics, Johns Hopkins University, Baltimore, MD 21205, USA
[14]Department of Anatomy and Anthropology & Department of Human Molecular Genetics and Biochemistry, Faculty of Medical and Health Sciences, Tel Aviv University, Tel Aviv 6997801, Israel
[15]Biohub – San Francisco, San Francisco, CA 94158, USA
[16]Lead contact
*Correspondence: altemose@stanford.edu (N.A.), khmiga@ucsc.edu (K.H.M.)
https://doi.org/10.1016/j.xgen.2026.101324

SUMMARY

Centromeres ensure chromosome segregation, but their chromatin organization within repetitive alpha-satellite DNA has been difficult to resolve. To address this, we generated haplotype-resolved satellite DNA annotations for the complete diploid T2T-HG002 human genome assembly, then we mapped centromere protein A (CENP-A), H3K9me3, and CpG methylation on ultra-long, adaptively sampled nanopore reads using directed methylation with long-read sequencing (DiMeLo-seq). We find that CENP-A occupies multiple discrete subdomains within hypomethylated centromere dip regions (CDRs), with constrained aggregate size and balanced CENP-A dosage between homologous chromosomes despite extensive satellite array variation. We also show that extended lymphoblastoid cell culture and induced pluripotent stem cell (iPSC) reprogramming remodel DNA methylation and alter CENP-A abundance and CDR subdomain organization. These results define a single-molecule, haplotype-resolved framework for studying human centromere plasticity, epigenetic inheritance, and chromosomal instability in development and disease.

INTRODUCTION

Centromeres are essential genomic loci that ensure accurate chromosome segregation, yet fundamental questions about their chromatin architecture remain unresolved.[1] Centromere protein A (CENP-A), a histone H3 variant, epigenetically specifies centromere identity and directs kinetochore assembly.[2,3] Rather than forming a single contiguous domain, CENP-A chromatin is organized into multiple discrete subdomains interspersed with canonical histone H3,[4] a pattern proposed to support higher-order 3D organization that underlies kinetochore structure and function.[5,6] CENP-A overexpression, often observed in cancer, can alter domain boundaries and destabilize centromere function.[7–10] Complete and highly accurate telomere-to-telomere (T2T) genome assemblies now enable detailed studies of the genetic and epigenetic organization of centromeres.[11–13] Prior studies in the T2T-CHM13 genome revealed that CENP-A chromatin coincides with centromere dip regions (CDRs) or localized hypomethylated domains within otherwise heavily methylated alpha-satellite (αSat) higher-order repeat (HOR) arrays.[14–18] CDRs show internal structure composed of multiple discrete hypomethylated "sub-CDRs" separated by methylated intervals, and

© 2026 The Authors. Published by Elsevier Inc.
This is an open access article under the CC BY license (http://creativecommons.org/licenses/by/4.0/).

they tend to localize to the youngest, most homogeneous repeats within each αSat array.[15,19] Complementing these assembly-based analyses, directed methylation with long-read sequencing (DiMeLo-seq), an antibody-directed protein-DNA interaction mapping approach combined with long-read sequencing, has enabled initial maps of CENP-A enrichment within satellite DNA.[20] While these early findings established the correlation between DNA methylation and CENP-A positioning, fundamental questions remain: what determines CENP-A subdomain boundaries? How does this multi-domain architecture vary between homologous chromosomes in diploid genomes, and how does it vary between different cell types from the same donor?

The HG002 genome provides a strong foundation for beginning to answer these questions by combining a complete, high-accuracy diploid assembly[21] with a well-characterized and widely available biological resource. HG002 is a Genome in a Bottle benchmark sample with a stable diploid XY karyotype, extensive publicly available sequencing data, and broad donor consent for research use.[22–24] Multiple matched cell resources are available, including the reference lymphoblastoid cell line (LCL), induced pluripotent stem cell (iPSC) lines derived from lymphoblastoid and peripheral blood cells, and parental cell lines, enabling integrated haplotype-resolved and cross-cell-type analyses. Importantly, the maternal and paternal chromosomes of HG002 are fully identifiable and traceable, enabling direct comparison of centromere organization between homologs of known parental origin rather than simply between unphased haplotypes.

Here, we pair detailed centromere satellite characterization within fully phased diploid T2T assembly with ultra-long-read, single-molecule chromatin profiling to define centromeric chromatin organization. To understand the underlying centromere satellite sequence, we generated haplotype-resolved satellite annotations and multi-scale maps of centromeric repeat structure, revealing extensive haplotype-specific variation in array size, internal repeat unit organization, and evolutionary layering of related satellite repeats within arrays. We then applied ultra-long-read DiMeLo-seq profiling of CENP-A and H3K9me3, a histone H3 lysine 9 trimethylation mark associated with pericentromeric heterochromatin,[25,26] to quantify centromeric chromatin architecture at single-molecule resolution. We show that CENP-A domains form multiple ordered subdomains whose aggregate size and protein dosage are broadly constrained across centromeres and between homologs. Finally, by comparing HG002 iPSCs and extended passaged HG002 LCLs, we show that changes in DNA methylation levels are accompanied by remodeling of subdomain boundaries and CENP-A domain spans, establishing methylation as a likely regulator of core centromeric chromatin architecture. Together, these results provide a diploid, single-molecule framework for studying centromere organization and its epigenetic plasticity across human genomes and cell states.

RESULTS

Haplotype-resolved annotation reveals multi-scale variation in human centromeres

The complete and highly accurate phased haplotype assemblies in the T2T-HG002 diploid human genome benchmark[21] enable comparative analysis of centromeric and pericentromeric regions across chromosomes and haplotypes. To support genetic and epigenetic studies of human centromeres, we implemented an automated tool suite to predict satellite DNA annotations across the diploid HG002 assembly (STAR Methods). Building on prior expert-curated satellite annotation tracks developed for the T2T CHM13 centromere assemblies, we applied an automated method to generate centromeric satellite ("CenSat") annotations in the diploid HG002 genome, enabling genome-wide classification of satellite families and systematic definition of array organization (Figures 1A and S1; Table S1; STAR Methods).

The diploid HG002 assembly contains ∼336 Mb of annotated satellite sequence, with αSat (∼174 Mb) and classical human satellites 1, 2, and 3 (HSat1–3, ∼138 Mb) constituting the largest fractions and with smaller contributions from beta-satellite (∼13 Mb) and other CenSat subclasses. Diploid, haplotype-resolved satellite annotation enables direct comparison of maternal and paternal chromosomes[13] and reveals widespread megabase-scale differences in centromeric and pericentromeric arrays, with at least 19 autosomal loci exhibiting haplotype-specific size differences greater than 0.5 Mb (Table S2). The largest haplotype-specific differences occur in pericentromeric HSat1–3 arrays and in active αSat arrays, with the most extreme example at the chromosome 9 (chr9) HSat3 locus, which differs by 10.1 Mb between haplotypes (21.07 Mb maternal vs. 11.00 Mb paternal) and contains large inversions and other structural rearrangements (Figure S2). HSat2 on chr1 differs by 6.74 Mb between homologs and contains distinct sequence compositions (STAR Methods; Table S3), while the paternal haplotype contains an additional ∼500 kb polymorphic block of HSat3 with an adjacent beta-satellite sequence (Figure S3). Interspersed HSat1A arrays are observed embedded within active αSat arrays on chr3 and chr4, differing in size by ∼0.61 Mb on chr3 and ∼1.1 Mb on chr4, indicating haplotype-specific restructuring and mixed satellite composition within kinetochore-associated centromeric domains (Figure S4). Finally, the αSat HOR array on chr5 shows the largest homolog-specific size difference among active arrays, with a total length of 9.49 Mb on the paternal haplotype and only 3.59 Mb on the maternal haplotype, most likely resulting from a large triplication event within the array (Figure 1B). Based on currently available assemblies, the 9.49 Mb paternal chr5 array appears to be the largest αSat array reported in a human genome to date.[11–13,19]

Differences between homologous centromeres are not limited to total array length but also extend to internal repeat organization and evolutionary structure within αSat arrays (Figure S5). αSat DNA is organized into multimeric HORs that form homogenized arrays associated with kinetochore assembly and centromere function. Some chromosomes have multiple αSat HOR arrays, but typically only one "active" HOR array contains the centromere, which occupies only a subregion of the active array, while the other HOR arrays remain inactive and do not contain CENPs across their entire length. Using HOR-level annotation together with structural variant (StV) classification to detect monomer-scale insertions and deletions within canonical HOR units, we observed broad variation in HOR structural composition across HG002

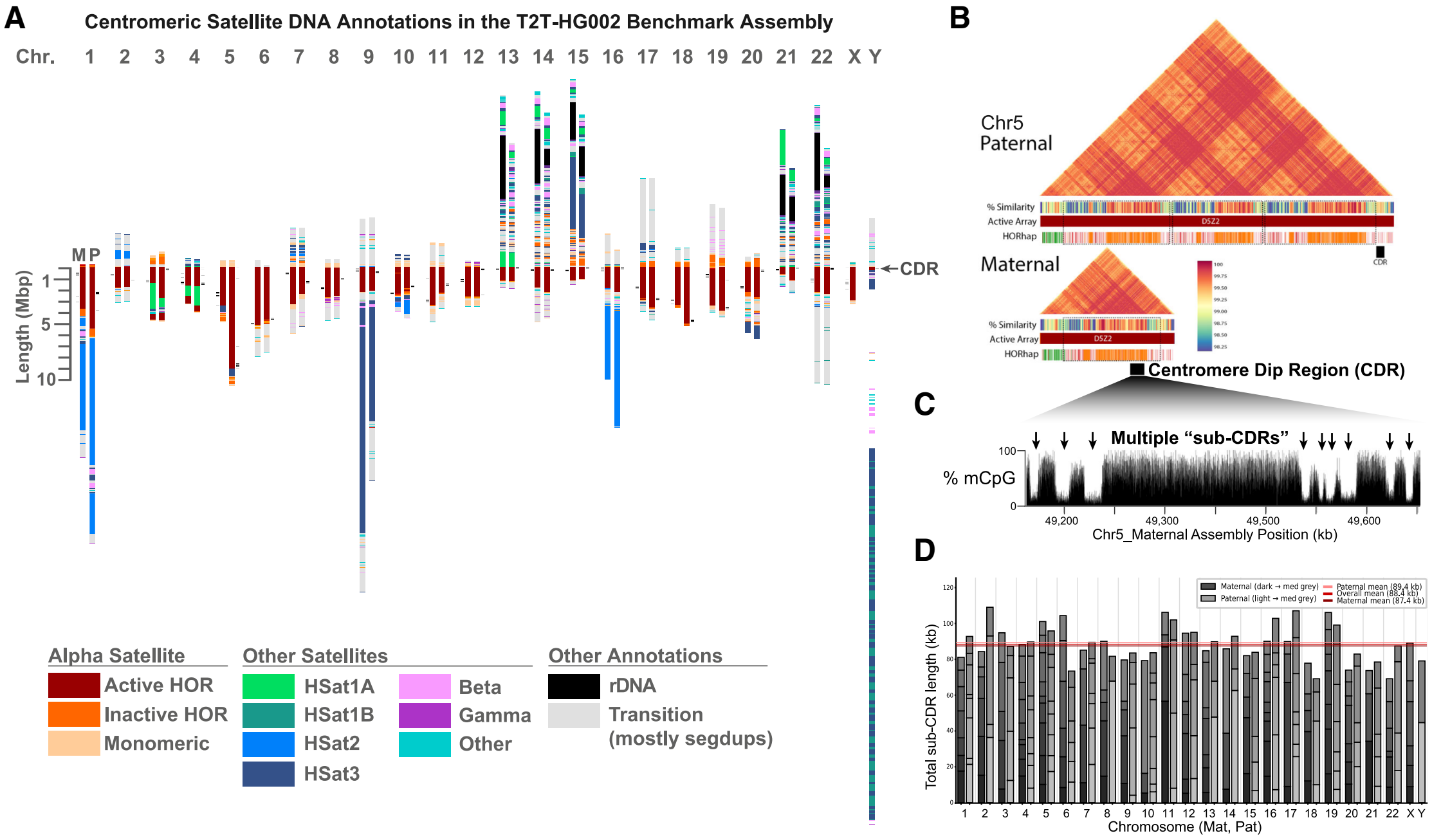

Figure 1. Haplotype-resolved centromere annotation reveals multi-scale variation in the diploid HG002 genome

(A) Centromeric satellite (CenSat) annotations for maternal ("M") and paternal ("P") chromosomes. Annotations are centered at the p-arm proximal boundary of the active array. Acrocentric short arms (13p, 14p, 15p, 21p, and 22p) are included due to their enrichment of satellite classes common to centromeric regions. The locations of centromere dip regions (CDRs) are indicated in black.

(B) Chromosome 5 paternal and maternal haplotypes illustrating the largest homolog-specific size difference observed among active αSat arrays (9.49 Mb paternal vs. 3.59 Mb maternal), with higher-order repeat haplotypes (HORhap) and a 1D modplot heatmap[27] and 1D projected ModDotPlot tracks. Repeat annotations highlight a triplication in the chromosome 5 paternal active array. CDRs are shown in black within the q-arm proximal region.

(C) CpG methylation track across the chromosome 5 maternal active αSat array, illustrating nine discrete sub-CDR hypomethylation dips.

(D) Stacked bar plot of sub-CDR lengths for all chromosomes, ordered by genomic position, with mean aggregate sub-CDR lengths indicated separately for maternal (dark red), paternal (light red), and all chromosomes combined (red). Despite extensive variation in active array length, total or aggregate sub-CDR lengths per chromosome appear similar between homologs and consistent between chromosomes genome-wide (mean: ∼88 kb, range: 69–109 kb).

centromeres (Figure S6; Table S4). Some active arrays are structurally uniform, including chr11, chr14, and chr22 on both haplotypes, along with chrX, where a single structural HOR variant accounts for at least 95% of units. In contrast, other centromeres show high structural diversity, most prominently chr19 on both haplotypes, followed by chr1, chr4, chr5, and chr10, each containing numerous distinct HOR StVs at appreciable frequency. Homologous centromeres can also differ markedly in variant composition even when total array sizes are similar. For example, the chr13 active arrays have comparable lengths but highly distinct variant profiles (Figure S7). To further resolve this structure, we grouped HOR units sharing characteristic sequence variants into haplotype classes, termed HORhaps (STAR Methods), and mapped their distribution across active arrays. HORhap composition often differs substantially between homologs, again illustrated by chr13, where profiles indicate near-complete haplotype turnover with only a small residual paternal haplotype tract at the edge of the maternal array (Figure S7). Using these HORhap patterns, we also created maps of superHORs, which are defined by recurring HOR combinations that occupy large fractions of several arrays, including chr8 (∼0.46 paternal, ∼0.51 maternal), chr15 (∼0.43 paternal, ∼0.40 maternal), and chr9 (∼0.33 paternal, ∼0.38 maternal) (STAR Methods).

To link the structural and evolutionary variation described above with functional centromere identity, we next analyzed CDRs, the localized hypomethylated domains within αSat HOR arrays that mark sites of CENP-A chromatin assembly (Figures 1A–1C; Table S5). Using genome-wide methylation profiles, we identified the span of each CDR and defined sub-CDR domains across active centromeres on both homologs (STAR Methods; Figures 1A–1C). When classified by array position, in agreement with prior studies,[28] we find a plurality of CDRs localized to the p-arm-proximal portion of the active array (41.3%), whereas fewer occurred near the q-arm (28.2%) (Figure S8). Consistent with prior observations in T2T-CHM13[15] and recent observations across many assemblies,[19] we observe that CDRs preferentially occur within more sequence-homogeneous αSat subregions (quantitative comparison of local HOR similarity; permutation test, $N = 100{,}000$, $p < 0.0001$), (Figure S9). Next, we asked whether CDRs preferentially overlapped younger or older HORhap domains. Although CDRs more frequently overlapped younger HORhaps (28 vs. 9 chromosomes), this trend was not statistically significant after accounting for their relative genomic proportions (binomial test, $p = 0.092$; see STAR Methods; Table S5).

Although the total span from the first to last sub-CDR varies across chromosomes and between maternal and paternal

homologs and dramatically across the genome (Figure S10), the summed length of sub-CDR segments is comparatively stable genome-wide, averaging ∼88 kb (range: 69–109 kb; Figure 1D), with no apparent relationship to the total active HOR array size (Figure S11). Despite extensive haplotype-specific differences in array size, structural composition, and inferred expansion age, the total hypomethylated core domain is remarkably constrained in length. Together, these results show that hypomethylated centromere cores can be reproducibly identified in diploid T2T assemblies, are associated with locally homogeneous αSat sequences, and maintain a shared functional sub-CDR aggregate length despite the highly variable sizes of the associated active HOR arrays.

Single-molecule profiling reveals CENP-A domain organization and dosage at centromeres

Having established haplotype-resolved centromeric sequence organization and well-defined CDRs, we next examined variation in centromeric chromatin structure across homologs. CENP-A-containing nucleosomes mark sites of kinetochore assembly, yet their precise relationship to CDRs remains unclear, including how consistently they colocalize with CDRs and whether CENP-A occupancy spans the full CDR or only subregions across a population of cells. To address this, we generated a high-coverage, ultra-long-read epigenomic dataset to enable detailed mapping of centromeric chromatin in HG002 LCLs. To do this, we performed DiMeLo-seq,[20] which combines antibody-targeted methylation with nanopore sequencing (STAR Methods), to directly map CENP-A-associated DNA interactions across HG002 active arrays. Because CDR spans typically extend ∼100–400 kb based on our HG002 analysis, high-coverage, ultra-long (100 kb+) nanopore reads are required to phase signals to maternal and paternal haplotypes, span the CDR, and measure CENP-A domain architecture on single DNA molecules. Achieving the resolution needed to quantify epigenetic variation across CDRs and active arrays requires deep coverage; however, CENP-A-associated regions constitute only a small fraction of the genome. Existing restriction-based centromere enrichment approaches require substantial input material and introduce handling steps that reduce achievable read length.[20,29] To overcome these constraints, we implemented nanopore adaptive sampling (with 100,000 bases flanking each active array; Table S6) after ultra-long library preparation, using a depletion strategy that selectively rejects non-αSat molecules in real time based on alignment of the initial few hundred bp of each read,[30] thereby enriching centromeric arrays while preserving ultra-long read lengths (N50 63 kb; with ∼20× coverage 100 kb+ reads; STAR Methods; Figure 2A). This strategy dramatically improved coverage of centromeric regions, as illustrated by the chrX αSat HOR array, where adaptive sampling achieves ∼54× coverage, which is about 30 times higher than the rest of the genome. Per flow cell, this approach achieves approximately 4-fold enriched coverage of centromeric regions without additional sample preparation (Figure 2A). Coverage is consistent across chromosomes and across each HOR array (Figure S12). Overall, this strategy yields a uniquely high-resolution, haplotype-resolved, centromere-targeted epigenomic dataset, establishing a foundation for systematic analysis of CENP-A chromatin across fully assembled human centromeres.

Alignment of DiMeLo-seq reads to the diploid HG002 assembly enabled haplotype-resolved chromatin profiling across active centromere arrays. Mappability analyses confirmed that reads at these lengths align confidently and with haplotype specificity to active centromere arrays (STAR Methods). Individual molecules showed multiple CENP-A-enriched segments that coincided with annotated sub-CDRs, and boundary-centered analyses (STAR Methods) across all centromeres revealed a consistent inverse relationship between CpG methylation and CENP-A enrichment, with the CENP-A signal increasing as CpG methylation decreased (Figures 2B and 2C). To define the flanking heterochromatin context, we also generated ultra-long DiMeLo-seq datasets targeting H3K9me3, a hallmark of pericentromeric heterochromatin that typically co-occurs with CpG hypermethylation. Across centromeres, H3K9me3 enrichment closely tracked CpG methylation and showed coordinated depletion at sub-CDR intervals, forming reciprocal patterns relative to the CENP-A signal and sharply delineating centromere core vs. pericentromeric domains (Figure 2C; consistent with other recent work).[32] The concordant CpG and H3K9me3 profiles, together with their exclusion from CENP-A-enriched regions, reveal a compartmentalized chromatin architecture at centromeres. We observed that sub-CDR boundaries sometimes displayed mixed methylation states, in which only a subset of reads retained high 5mC, while others were hypomethylated, consistent with plasticity in exact sub-CDR boundaries across the population of cells. Clustering based on CpG signal separated these reads into multiple 5mC-defined groups (STAR Methods), supporting the interpretation that these patterns reflect genuine molecule-level epigenetic heterogeneity rather than measurement noise (Figure S13). To further resolve chromatin footprints at single-molecule resolution, we generated and analyzed nanopore ultra-long Fiber-seq[33] data for HG002 LCLs (STAR Methods). Consistent with prior observations,[34] we observed smaller nucleosome footprints within sub-CDRs in the active arrays (vs. non-active arrays and outside of sub-CDRs), with positioning adjacent to canonical CENP-B box motifs (Figure S14). Notably, the reduced footprint size is consistent with prior reports that CENP-A-containing nucleosomes adopt a more compact structure than canonical H3 nucleosomes,[35] supporting the interpretation that these protected regions reflect centromere-specific chromatin architecture.

Given that the aggregate length of sub-CDR intervals is similar across centromeres and between homologs, we next tested whether CENP-A signal is also quantitatively balanced across these domains. We estimated CENP-A dosage using DiMeLo-seq 6mA signal density, defined as the number of detected mA bases divided by the total number of adenine positions within each interval (STAR Methods). CENP-A density is largely consistent across sub-CDRs and shows similar levels between homologous centromeres, matching the constrained total span of aggregated sub-CDR domains (Figure 2D). These measurements indicate that CENP-A chromatin dosage is broadly balanced across human centromeres (Figure 2E), consistent with prior GFP-based estimates of genome-wide CENP-A abundance.[36] In further support of this observation,

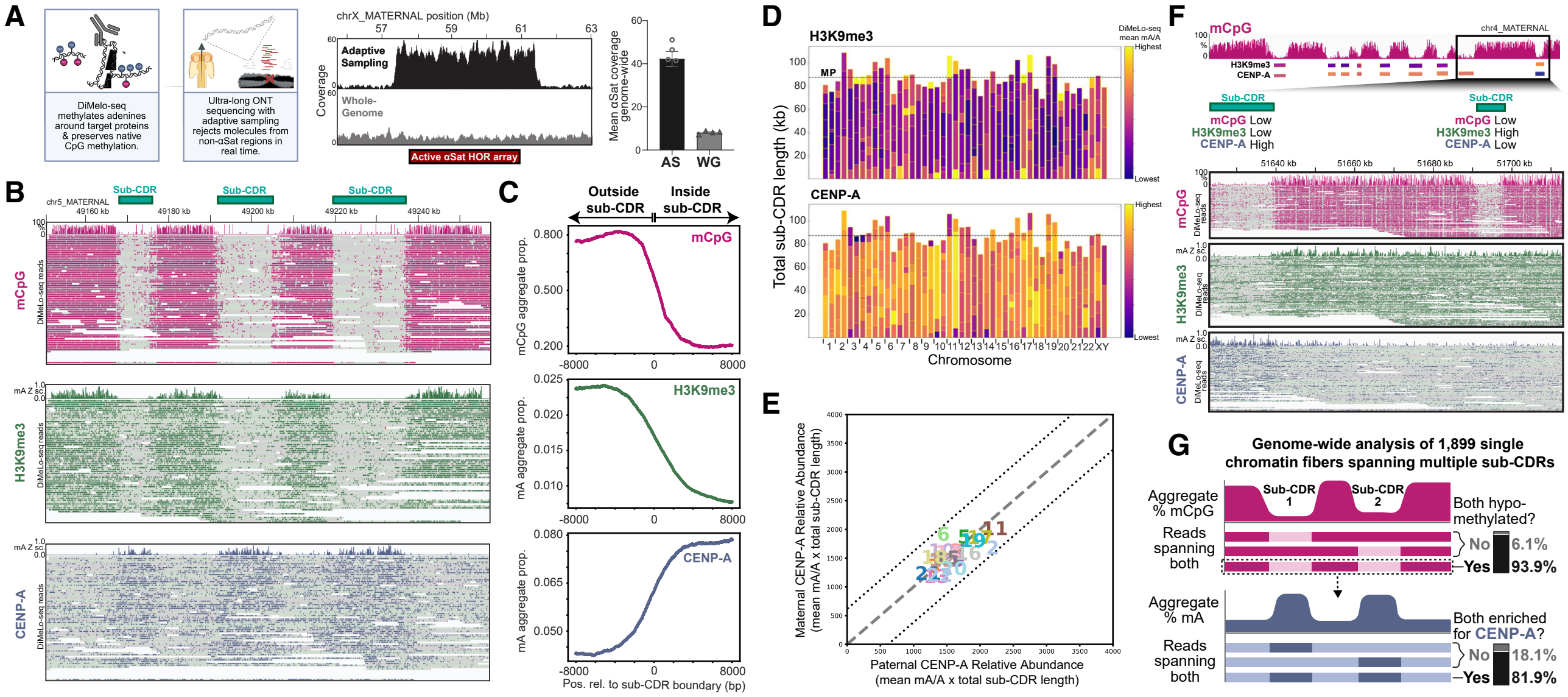

Figure 2. Single-molecule profiling of CENP-A chromatin architecture across haplotype-resolved human centromeres

(A) Schematic of the adaptive sampling DiMeLo-seq enrichment strategy and data processing pipeline. Coverage comparisons between adaptive sampling and whole-genome sequencing are shown for the chromosome X αSat HOR array and genome-wide, illustrating ~30-fold enrichment of centromeric regions relative to the rest of the genome.

(B) Integrative Genomics Viewer (IGV)[31] visualization of a region spanning a CDR on chromosome 5 (chr5_MATERNAL:49150874–49257508) within the active αSat array. mCpG (magenta), H3K9me3 (green), and CENP-A (blue) DiMeLo-seq signal tracks are shown, illustrating the relationship between CENP-A enrichment and the heterochromatic (H3K9me3 and mCpG) boundaries of sub-CDR intervals (indicated by black bars).

(C) Aggregate profile plots of mCpG, H3K9me3, and CENP-A signals centered on CDR boundaries across all centromeres, demonstrating an inverse relationship between CpG hypomethylation and CENP-A enrichment relative to flanking H3K9me3-marked heterochromatin.

(D) Stacked heatmaps of H3K9me3 (top) and CENP-A (bottom) DiMeLo-seq 6mA signal density across all aggregated sub-CDRs, ordered by chromosomal position and separated by maternal and paternal haplotype. The dotted line indicates the mean length (~90 kb) of aggregate sub-CDRs. The majority of sub-CDRs display an inverse relationship between CENP-A and H3K9me3 (where the CENP-A signal is high and the H3K9me3 signal is low), with variation observed primarily in the outermost sub-CDRs.

(E) Scatterplot comparing DiMeLo-seq CENP-A 6mA relative abundance between maternal and paternal homologs across all centromeres, showing a broadly balanced signal between haplotypes.

(F) IGV visualization of an atypical sub-CDR at chromosome 4 (chr4_MATERNAL:51622694–51707732), where CENP-A enrichment is absent, and H3K9me3 is present within the sub-CDR, representing a departure from the canonical centromeric chromatin architecture in which H3K9me3 and mCpG are excluded from sub-CDRs.

(G) Schematic illustrating co-occupancy of CENP-A across multiple sub-CDRs on individual ultra-long nanopore reads, as assessed using a decision-tree classifier trained on single-molecule signals from CDR and non-CDR regions. The majority of reads spanning two sub-CDRs exhibit CENP-A enrichment at both sub-CDRs.

we observe CENP-C DiMeLo-seq mA density to also be quantitatively balanced across chromosomes and between homologous centromeres (Figure S15). Notably, the greatest variability occurs in smaller, outer sub-CDRs, where reduced CENP-A density is accompanied by an increased H3K9me3 signal (Figure 2D). These intervals mark localized departures from the typical reciprocal pattern among CENP-A, CpG hypomethylation, and H3K9me3, including sites where sub-CDR methylation signatures are present without corresponding CENP-A enrichment and, conversely, where heterochromatin marking extends into hypomethylated sub-CDR regions (Figure 2F). Overall, these dosage and boundary patterns indicate that CENP-A is broadly balanced across centromeres and between homologs, while limited edge variability is detectable at a fine scale.

We next leveraged the unique ultra-long, single-molecule nature of our DiMeLo-seq data to test whether multiple sub-CDR domains are co-occupied by CENP-A on the same chromatin fibers (as illustrated in Figure 2G) or whether they arise from averaging across heterogeneous cell populations. Single-molecule analysis supports the former. Of 247,716 ultra-long reads overlapping at least one sub-CDR, 1,899 fully span two or more sub-CDRs. Among these spanning reads, 93.88% show mCpG depletion across all annotated sub-CDR intervals, consistent with the predicted spacing and organization of sub-CDRs, with only a small fraction indicating variation in sub-CDR structure. CENP-A enrichment patterns show similar concordance, with 81.85% of reads spanning multiple sub-CDRs exhibiting the CENP-A signal at more than one sub-CDR on the same molecule, whereas 18.15% lack signal at one or more spanned sub-CDRs (Figure 2G). Together, these single-molecule sequencing results indicate that the majority of centromeres in the sampled cell population contain multiple CENP-A-associated subdomains while also revealing measurable cell-to-cell variation in sub-CDR occupancy by CENP-A.

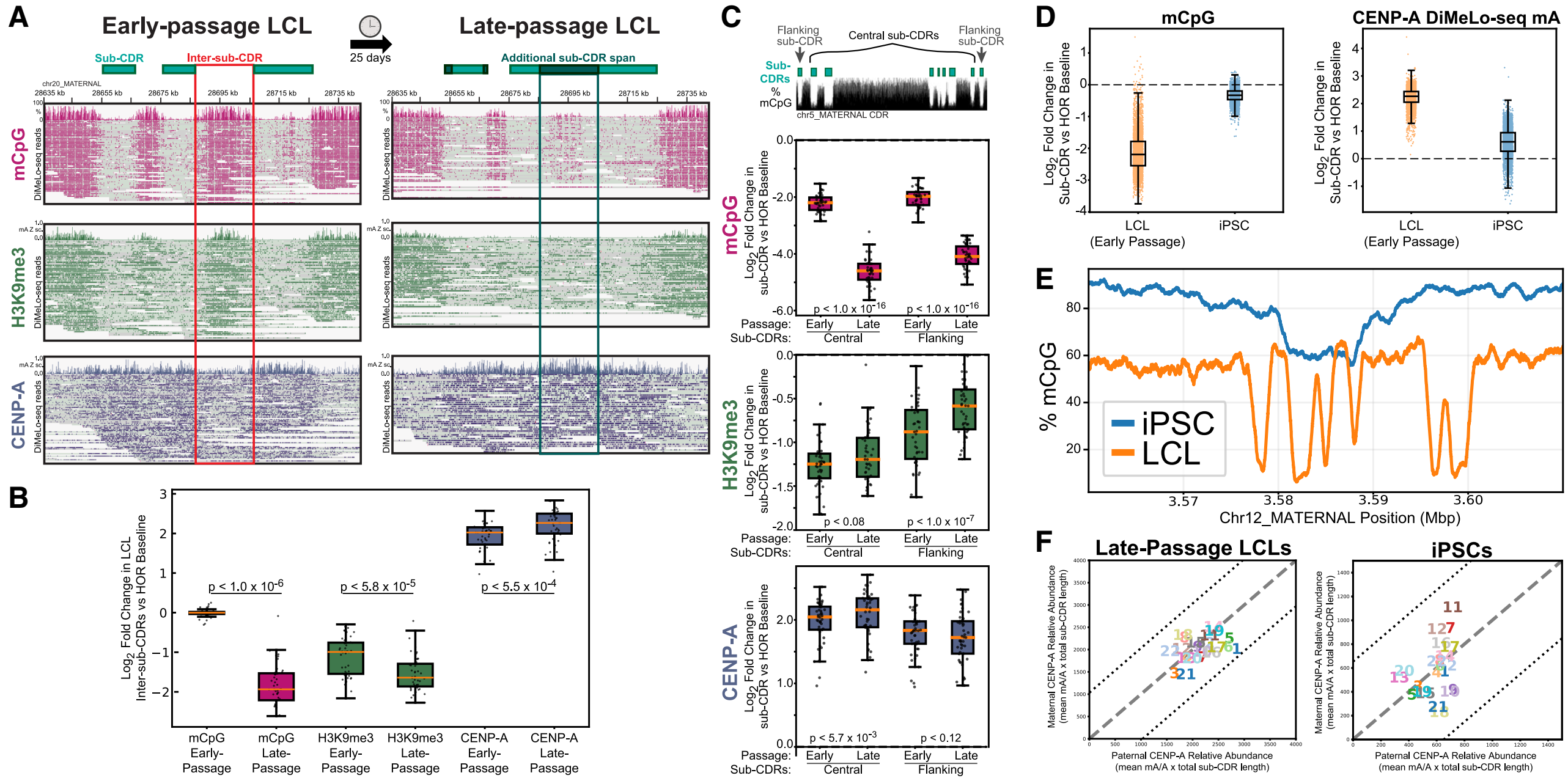

Figure 3. DNA methylation state influences sub-CDR boundaries and CENP-A domain organization

(A) IGV visualization of the chromosome 20 (chr20_MATERNAL:28635505–28746668) CDR region showing mCpG (magenta), H3K9me3 (green), and CENP-A (blue) DiMeLo-seq signal tracks in early- and late-passage (25 days) HG002 LCLs. Sub-CDRs are highlighted in light green. Sites where methylated intervals separating neighboring sub-CDRs were diminished are indicated by dark green bars and a red box, resulting in more frequent formation of continuous CENP-A-occupied regions.

(B) Log_2 fold change of mCpG, H3K9me3, and CENP-A DiMeLo-seq signal densities at inter-sub-CDR boundaries between early- and late-passage LCLs. Within regions of boundary loss, there is a significant decrease in mCpG between early and late passages, a concurrent decrease in H3K9me3, and a corresponding increase in CENP-A.

(C) Illustration of central and flanking sub-CDRs in the chr5_MATERNAL CDR region. Genome-wide fold change plots show CENP-A density increasing in central sub-CDRs but not in flanking sub-CDRs in late-passage LCLs, with H3K9me3 showing the reciprocal pattern and mCpG remaining largely unchanged across sub-CDR positions.

(D) Fold change analysis of mCpG and CENP-A signal densities across 1 kb bins within sub-CDRs in HG002 early-passage LCLs and iPSCs, with non-CDR active array regions used as a baseline. HG002 hiPSCs show a reduction in CENP-A enrichment and an increase in CpG methylation within the active array.

(E) Line plot comparing sub-CDR positions in hiPSCs and LCLs for chr12_MATERNAL. The hiPSC active array shows elevated CpG methylation in the active array (~90%) and within sub-CDRs (~60%) relative to LCLs (~60% and ~20%, respectively). Although the CDR in hiPSCs is broader than in LCLs, it occupies only a partial span of the corresponding LCL CDR.

(F) Scatterplots of relative CENP-A abundance, estimated by multiplying mean 6mA/A density by total or aggregate sub-CDR(s) length, for maternal and paternal homologs in late-passage LCLs and iPSCs. Despite extensive sub-CDR reorganization, relative CENP-A abundance remains proportional across chromosomes and balanced between homologs.

DNA methylation state controls sub-CDR boundaries and CENP-A domain size

Under typical conditions in HG002 lymphoblastoid cells, centromeric chromatin is organized into distinct and functionally specialized domains. In this organization, discrete islands of CENP-A chromatin are interspersed and largely mutually exclusive with H3K9me3-enriched, CpG-methylated pericentromeric heterochromatin. However, it remains poorly understood whether this fine-scale epigenetic architecture is robust to larger epigenetic changes across different cell types and cell states. To begin to test this, we passaged HG002 LCLs for an additional 25 days (which we refer to as "late-passage" cells), as extended culture of transformed LCLs has been associated with CpG methylation changes,[37] which may in turn affect centromere organization. We generated ONT ultra-long DiMeLo-seq profiles for CENP-A and H3K9me3 in these late-passage cells (STAR Methods) and compared them to our initial (or "early-passage") sub-CDR analyses (Figure S16). We found that methylated intervals separating neighboring sub-CDRs were diminished (Figure 3A), and the H3K9me3 signal was reduced at inter-sub-CDR boundaries, resulting in more frequent formation of continuous CENP-A-occupied regions (Figures 3A and 3B). Sub-CDR expansion and consolidation were reported genome-wide, with a 16.9% reduction in sub-CDR number (254–211) accompanied by a 444 kb increase in total CDR length (increasing by 10.9%) (Table S7; Figure S17). This reorganization was primarily driven by boundary resizing rather than wholesale domain gain or loss: 97.2% of early-passage sub-CDRs (247 of 254) were maintained in late-passage cells, with only 7 sub-CDRs lost entirely and 4 novel sub-CDRs appearing. Of the maintained sub-CDRs, 184 (74.5%) expanded, while 63 contracted, further supporting that boundary shifts, rather than *de novo* domain turnover, are the principal mode of reorganization. Sub-CDRs were correspondingly larger, with mean length increasing from

16 to 21.4 kb and maximum sub-CDR size expanding to ~100 kb in late-passage cells compared to 68 kb in early-passage ones. This reorganization was widespread, with 17 of 24 chromosomes showing reduced sub-CDR counts and most chromosomes exhibiting increased total CDR span, although several centromeres showed minimal change (<10%), including chr3, chr17, and chrY, indicating chromosome-specific responses. Further, sub-CDR remodeling was positionally biased; that is, centrally located sub-CDRs preferentially expanded, whereas sub-CDRs flanking CDR boundaries more often gained the H3K9me3 signal in late-passage cells (Figure 3C). CpG methylation levels did not differ between central and outer sub-CDRs, indicating that boundary-proximal CENP-A loss and heterochromatin gain are not explained by local CpG differences (Figure S18). Collectively, these results show that methylation loss is associated with boundary erosion and positionally structured sub-CDR remodeling, leading to consolidation of CENP-A chromatin into fewer, larger centromeric domains.

Having observed that methylation loss is accompanied by sub-CDR boundary erosion and CENP-A domain expansion, we next asked whether, conversely, elevated DNA methylation levels associate with reciprocal changes in centromeric domain organization. To test this, we analyzed CENP-A and sub-CDR architecture in donor-matched HG002 iPSCs (with quality assessment in STAR Methods), which show increased CpG methylation across active centromeric arrays relative to early-passage LCLs (Figure 3D; STAR Methods). Whereas LCL arrays typically exhibit ~60% CpG methylation with pronounced CDR dips below 20%, iPSC arrays are more uniformly methylated (~90%) and display only shallow CDR depressions (~60%) (Figure 3E). Under these hypermethylated conditions, CDR organization was extensively reorganized (Figure S19; Table S8). Total sub-CDR number decreased approximately 2-fold (254–118), while mean CDR length increased 2.5-fold (16.0–40 kb), producing a strong shift toward large continuous domains with maximum sizes reaching ~168 kb (Figure S16). Despite this, total CDR coverage increased (4.1 Mb in early-passage LCLs to 4.8 Mb in human iPSCs [hiPSCs]), indicating genome-wide merging of adjacent sub-CDRs. The majority of centromeres showed increased total CDR span despite fewer domains, with the largest gains on chr7, chr11, chr14, and chr17 (Figure S19). Notably, sequence similarity analysis (1D ModDotPlot similarity data) indicates that these methylation-associated domain expansions are likely not driven by underlying sequence identity, as regions incorporated into expanded iPSC domains are indistinguishable from former LCL inter-sub-CDR gaps, and no consistent directional shift in similarity is observed across chromosomes. We next examined whether the reorganization of sub-CDRs was accompanied by changes in centromeric chromatin composition and found that CENP-A density also decreased significantly relative to LCLs (Figure 3D). However, although overall CENP-A density is reduced, the DiMeLo-seq 6mA signal density within individual sub-CDRs remains largely equivalent across chromosomes and between homologs, with average sub-CDR DiMeLo-seq 6mA signals within 2-fold, with two notable exceptions describing small sub-CDRs at a distance with weak support (Figures 3F and S20; STAR Methods). While most homolog pairs show consistent 6mA signal density and aggregate sub-CDR lengths, a small subset of chromosomes display inter-homolog asymmetry (Figures 3F and S20), which may reflect biological variability or technical limitations in defining sub-CDR boundaries within highly methylated active arrays. Notably, similar to early-passage lines (Figure 2E), we observe a preservation of relative CENP-A DiMeLo-seq 6mA signal density under hypomethylated conditions in late-passage LCLs: although sub-CDRs expanded and boundaries eroded, homolog balance was roughly maintained (Figures 3F and S21). Whether this plasticity in centromeric architecture is a general feature—or whether it is amplified in pluripotent cells, where hypomethylation is pervasive and epigenetic landscapes are broadly reshaped—remains an important open question. Together, these findings support a model in which centromeric chromatin exhibits methylation-sensitive architectural plasticity, where shifts in DNA methylation levels reshape sub-CDR organization.

DISCUSSION

Our work has provided insights into the organization and heterogeneity of human centromeres enabled by the HG002 benchmark diploid assembly in combination with long-read, single-molecule epigenomic technologies. By building and deploying automated satellite DNA annotation tools, we uncovered substantial variation in satellite DNA array size, structure, and sequence composition when comparing the maternal and paternal haplotypes of HG002. Furthermore, we optimized ONT's adaptive sampling method to enable enriched coverage of centromeric regions without any additional sample preparation steps,[20] overcoming challenges due to the repetitiveness and high polymorphism of these regions. By combining this approach with ultra-long nanopore sequencing, we obtained single-molecule reads that could span across CDRs, which are clusters of hypomethylated sub-CDR regions that form the core of human centromeres. Using DiMeLo-seq, we could use these long, centromere-enriched reads to examine the localization of CENP-A and H3K9me3 at fine scales while simultaneously measuring endogenous mCpG on the same single chromatin fibers.

These unique data enabled us to measure several fundamental properties of human centromeres. We observed that centromeres in HG002 LCLs contain on average ~5.5 hypomethylated sub-CDR regions (range: 2–9), which on average have a summed length of 88 kb (range: 69–109 kb), irrespective of the number of sub-CDRs or the total length of the active αSat array on that chromosome. From DiMeLo-seq data, we also found that the mean intensity of CENP-A-targeted DiMeLo-seq signal was relatively constant across haplotypes and chromosomes. Together, these data suggest that the span of centromeric chromatin and the dose of CENP-A per chromosome are fairly uniform across chromosomes despite high variation in αSat array size, consistent with prior work from quantitative imaging of CENP-A.[36] Notably, the size, organization, and cell-to-cell heterogeneity of CENP-A binding domains we observe within αSat arrays are consistent with those described at satellite-free centromeres in other mammalian species and at non-repetitive neocentromeres in humans, where CENP-A domains of comparable size and interindividual positional variation have been

characterized in the absence of satellite DNA.[38] In horses, donkeys, and zebras, CENP-A binding domains span regions of a few hundred kilobases and exhibit positional variation between individuals, giving rise to heritable epialleles through centromere sliding.[39–42] This parallel organization across fundamentally different sequence contexts is consistent with centromere size, spacing, and epigenetic heterogeneity being influenced by factors beyond primary satellite DNA sequence alone. However, this does not exclude a role for satellite arrays, and shared sequence features within and beyond these regions remain incompletely characterized.

We observed at fine scales on single chromatin fibers that CENP-A localization is strongly anti-correlated with mCpG, while H3K9me3 is strongly correlated with mCpG, consistent with other recent work using DiMeLo-seq.[19,26,32,43,44] However, our single-molecule data allow us to observe important exceptions to these trends. For example, we observed several hypomethylated regions that lack CENP-A and are enriched for H3K9me3. Furthermore, given our ultra-long sequencing reads, we observed that distinct sub-CDRs tend to be co-occupied by CENP-A on the same chromatin fibers. This allows us to rule out a model in which CENP-A heterogeneously occupies one of several sub-CDRs in each cell. Thus, on single chromatin fibers, CENP-A is highly enriched within multiple neighboring spans of hypomethylated DNA separated by hypermethylated DNA enriched for H3K9me3. Perhaps these separate domains of CENP-A along single chromatin fibers can explain recent observations of multi-partite mitotic centromere structures using super-resolution microscopy.[6]

Intriguingly, we also observed that the length and number of individual sub-CDRs vary across chromosomes and correlate with changes in array methylation due to cell passaging or reprogramming. This raises the possibility that shifts in sub-CDR organization and in CENP-A balance may influence the capacity to form and faithfully inherit multi-partite centromere architectures. Understanding what drives this variability and why certain sub-CDRs on particular chromosomes are more susceptible to reorganization than others remains an open question. An important and unanswered question is whether shifts in sub-CDR organization occur naturally over time within an individual, for example, during aging, tissue differentiation, or in response to environmental epigenetic perturbations. If so, centromeric chromatin remodeling may represent an underreported somatic epigenetic variation with potential consequences for long-term chromosome segregation fidelity. We recently demonstrated in separate work that perturbing DNA methylation at centromeres causally alters CENP-A localization and centromere function.[44] Others have recently shown that perturbing pericentric histone modifications can lead to loss of centromeric chromatin confinement[26,32,43] and that fine-scale sub-CDR organization can undergo dramatic shifts in cancer cells[9] and sperm cells.[45] While the mechanistic basis for CDR hypomethylation remains unresolved, one intriguing possibility is that localized loss of DNA methylation at sub-CDRs may create a permissive chromatin environment for transient transcriptional activity, which has been proposed to facilitate CENP-A loading; however, rigorously testing this hypothesis will require more sophisticated RNA-based experimental approaches than are currently available for these highly repetitive regions.[46]

Beyond DNA methylation, the molecular mechanisms that establish and maintain sub-CDR boundaries and spacing remain poorly understood. Higher-order chromatin features, such as centromere-specific looping architectures, phase-separated chromatin compartments, or other secondary structures not yet well characterized in this context, may play important roles in organizing the multi-partite centromere core and represent compelling targets for future investigation. Previous work has shown, using microscopy, that cellular reprogramming induces depletion of CENP-A at centromeres, accompanied by increases in segregation errors.[47] Our DiMeLo-seq results in HG002 iPSCs confirm this loss of CENP-A with an orthogonal genomic approach and further demonstrate that this loss of CENP-A is accompanied by DNA hypermethylation and restructuring of the fine-scale organization of centromeric chromatin. Together, these insights suggest that the mechanisms governing centromeric architecture and function are complex and still not fully resolved, presenting an exciting opportunity for future exploration. The rich sequence annotation and scalable tools presented here, when combined with DiMeLo-seq across diverse cell types and additional genome assemblies, will provide a foundation for future investigation of the genetic and epigenetic influences on sub-CDR organization and its inheritance.

Limitations of the study

Our conclusions derive from a single donor genome, HG002, which is a benchmark XY lymphoblastoid line (LCL) and its derived iPSCs. This constrains the generalizability of the haplotype-resolved patterns we describe. Comparisons across many individuals, additional karyotypes, and primary, non-transformed tissues will be needed to establish how broadly the number, size, spacing, and inter-homolog balance of CENP-A subdomains are conserved. Importantly, the relationship we report between DNA methylation and centromeric chromatin architecture is associative rather than causal: it rests on comparisons between early- and late-passage LCLs and between LCLs and iPSCs, contexts in which extended culture of Epstein-Barr virus (EBV)-transformed cells and cellular reprogramming each remodel many epigenetic and transcriptional features beyond CpG methylation. We did not directly manipulate DNA methylation in the present study, although targeted perturbations of centromeric methylation reported in related work support a causal role.[44]

Several technical considerations further bound our interpretations. DiMeLo-seq measures antibody-directed adenine methylation density as an indirect, relative proxy for protein occupancy; our CENP-A "dosage" estimates therefore reflect relative signal rather than absolute molecule counts and depend on antibody specificity and epitope accessibility within centromeric chromatin. Sub-CDR boundary definition is intrinsically imprecise where domain edges display mixed methylation across the cell population and is more difficult within the uniformly hypermethylated active arrays of iPSCs; accordingly, some inter-homolog asymmetries we observe may reflect these measurement limits rather than true biological differences. Finally, our study characterizes chromatin organization but does not directly assay the

functional consequences—kinetochore assembly or chromosome segregation fidelity—of the architectural changes we describe, nor could we test the proposed link between sub-CDR hypomethylation and permissive transcription, which awaits RNA-based approaches suited to these highly repetitive regions.

RESOURCE AVAILABILITY

Lead contact

Requests for further information and resources should be directed to and will be fulfilled by the lead contact, Karen H. Miga (khmiga@ucsc.edu).

Materials availability

This study did not generate new unique reagents. The LCL GM24385, hiPSC line GM26105 (reprogrammed from GM24385), and hiPSC line GM27730 (reprogrammed from fibroblasts) were obtained from the NIGMS Human Genetic Cell Repository at the Coriell Institute for Medical Research.

Data and code availability

- DiMeLo-seq and nanopore ONT-UL genomic datasets have been deposited at SRA: PRJNA1451277 and are publicly available as of the date of publication.
- This paper analyzes existing, publicly available data, accessible at SRA: SRX14226557 and SRA: SRX14226558.
- All original code has been deposited at Zenodo: https://doi.org/10.5281/zenodo.20214995 and is publicly available as of the date of publication.
- Any additional information required to reanalyze the data reported in this paper is available from the lead contact upon request.

ACKNOWLEDGMENTS

Grants supporting this work are from the US National Institutes of Health (NIH), awarded to K.H.M. (NIH/NHGRI R01 1R01HG011274 and UM1 HG010971). H.L. is supported by NIH training grant T32HG123442. D.D. is supported by NIH training grant T32GM141828. F.R. was supported by the HSE Basic Research Program. M.C. is supported by the MUNI Award in Science and Humanities StG/CoG (MUNI/SC/1916/2024). T.A.P. is supported by R50CA305001 and SIMR. J.L.G. is supported by R01CA266339 and SIMR. N.A. is an HHMI Hanna H. Gray Fellow, Pew Biomedical Scholar, and Biohub Investigator. K.H.M. was supported in part by the Searle Scholars Program.

AUTHOR CONTRIBUTIONS

K.H.M., N.A., and Y.X. conceived of the study. Y.X., J.M.V.G., and B.M. performed the experiments. Y.X., H.L., J.M., F.R., J.K.L., M.C., L.M., E.X., D.D., M.A., I.V., C.O., T.H., S.O'R., W.T., and I.A.A. analyzed the data. C.C., T.A.P., J.L.G., J.P.S., A.F.S., and M.W.M. provided key reagents and samples. K.H.M., N.A., and Y.X. wrote the paper. K.H.M. and N.A. supervised the study.

DECLARATION OF INTERESTS

N.A. is named as an inventor on patent applications related to DiMeLo-seq.

DECLARATION OF GENERATIVE AI AND AI-ASSISTED TECHNOLOGIES IN THE WRITING PROCESS

During the preparation of this work, the authors used LLMs to assist with specific programming tasks and on rare occasions to improve the language of the manuscript. After using these services, the authors reviewed and edited the content as needed and take full responsibility for the content of the published article.

STAR★METHODS

Detailed methods are provided in the online version of this paper and include the following:

- KEY RESOURCES TABLE
- EXPERIMENTAL MODEL AND STUDY PARTICIPANT DETAILS
  - Cell lines
  - Influence of sex and gender on results
  - Ethical oversight
- METHOD DETAILS
  - Centromere satellite annotation tool suite
  - Higher-order repeat haplotype (HORhap) analysis
  - HORhap age analysis
  - SuperHOR annotation
  - Subfamily characterization of human satellites
  - Local identity calculation
  - Centromere mappability assessment
  - Quality control and characterization of hiPSC lines
  - CDR predictions
  - Fiber-seq
  - Cell culture and passage
  - DiMeLo-seq
  - DNA extraction
  - ONT ultra-long PromethION library prep and sequencing
  - PromethION adaptive sampling
  - DiMeLo-seq analysis
  - mA visualization and aggregate quantification
  - Single-molecule analysis of sub-CDR co-occupancy
  - Epigenetic comparisons between flanking and central CDRs in early-passage LCLs and late-passage LCLs
  - Epigenetic comparisons between differentially methylated regions in early-passage LCLs and late-passage LCLs
  - Epigenetic comparisons between centromeric differentially methylated regions in early-passage LCLs and iPSCs
  - Single-molecule read clustering based on methylation
- QUANTIFICATION AND STATISTICAL ANALYSIS
  - Dosage analysis
  - Read-level density thresholding
  - Density normalization
  - Epigenetic comparisons between early- and late-passage cells, or LCL and iPSCs
  - hiPSC CDR prediction

SUPPLEMENTAL INFORMATION

Supplemental information can be found online at https://doi.org/10.1016/j.xgen.2026.101324.

Received: March 6, 2026
Revised: July 12, 2026
Accepted: July 13, 2026
Published: August 6, 2026

REFERENCES

1. Jaggi, K.E., Hoyt, S.J., O'Neill, R.J., and Sullivan, B.A. (2026). A genomic and epigenomic view of human centromeres. Nat. Rev. Genet. *27*, 371–384. https://doi.org/10.1038/s41576-025-00923-1.
2. Black, B.E., and Cleveland, D.W. (2011). Epigenetic centromere propagation and the nature of CENP-a nucleosomes. Cell *144*, 471–479. https://doi.org/10.1016/j.cell.2011.02.002.
3. Van Hooser, A.A., Ouspenski, I.I., Gregson, H.C., Starr, D.A., Yen, T.J., Goldberg, M.L., Yokomori, K., Earnshaw, W.C., Sullivan, K.F., and Brinkley, B.R. (2001). Specification of kinetochore-forming chromatin by the

histone H3 variant CENP-A. J. Cell Sci. *114*, 3529–3542. https://doi.org/10.1242/jcs.114.19.3529.

4. Sullivan, B.A., and Karpen, G.H. (2004). Centromeric chromatin exhibits a histone modification pattern that is distinct from both euchromatin and heterochromatin. Nat. Struct. Mol. Biol. *11*, 1076–1083. https://doi.org/10.1038/nsmb845.
5. Blower, M.D., Sullivan, B.A., and Karpen, G.H. (2002). Conserved organization of centromeric chromatin in flies and humans. Dev. Cell *2*, 319–330. https://doi.org/10.1016/s1534-5807(02)00135-1.
6. Sacristan, C., Samejima, K., Ruiz, L.A., Deb, M., Lambers, M.L.A., Buckle, A., Brackley, C.A., Robertson, D., Hori, T., Webb, S., et al. (2024). Vertebrate centromeres in mitosis are functionally bipartite structures stabilized by cohesin. Cell *187*, 3006–3023.e26. https://doi.org/10.1016/j.cell.2024.04.014.
7. Shrestha, R.L., Rossi, A., Wangsa, D., Hogan, A.K., Zaldana, K.S., Suva, E., Chung, Y.J., Sanders, C.L., Difilippantonio, S., Karpova, T.S., et al. (2021). CENP-A overexpression promotes aneuploidy with karyotypic heterogeneity. J. Cell Biol. *220*, e202007195. https://doi.org/10.1083/jcb.202007195.
8. Tomonaga, T., Matsushita, K., Yamaguchi, S., Oohashi, T., Shimada, H., Ochiai, T., Yoda, K., and Nomura, F. (2003). Overexpression and mistargeting of centromere protein-A in human primary colorectal cancer. Cancer Res. *63*, 3511–3516.
9. Sohn, M.-H., Dubocanin, D., Vollger, M.R., Kwon, Y., Minkina, A., Munson, K.M., Hart, S.F., Ranchalis, J.E., Parmalee, N.L., Sedeño-Cortés, A.E., et al. (2025). A telomere-to-telomere map of somatic mutation burden and functional impact in cancer. Preprint at bioRxiv. https://doi.org/10.1101/2025.10.10.681725.
10. Sullivan, L.L., Boivin, C.D., Mravinac, B., Song, I.Y., and Sullivan, B.A. (2011). Genomic size of CENP-A domain is proportional to total alpha satellite array size at human centromeres and expands in cancer cells. Chromosome Res. *19*, 457–470. https://doi.org/10.1007/s10577-011-9208-5.
11. Nurk, S., Koren, S., Rhie, A., Rautiainen, M., Bzikadze, A.V., Mikheenko, A., Vollger, M.R., Altemose, N., Uralsky, L., Gershman, A., et al. (2022). The complete sequence of a human genome. Science *376*, 44–53. https://doi.org/10.1126/science.abj6987.
12. Logsdon, G.A., Rozanski, A.N., Ryabov, F., Potapova, T., Shepelev, V.A., Catacchio, C.R., Porubsky, D., Mao, Y., Yoo, D., Rautiainen, M., et al. (2024). The variation and evolution of complete human centromeres. Nature *629*, 136–145. https://doi.org/10.1038/s41586-024-07278-3.
13. Volpe, E., Colantoni, A., Corda, L., Di Tommaso, E., Pelliccia, F., Ottalevi, R., Guarracino, A., Licastro, D., Faino, L., Capulli, M., et al. (2025). The reference genome of the human diploid cell line RPE-1. Nat. Commun. *16*, 7751. https://doi.org/10.1038/s41467-025-62428-z.
14. Logsdon, G.A., Vollger, M.R., Hsieh, P., Mao, Y., Liskovykh, M.A., Koren, S., Nurk, S., Mercuri, L., Dishuck, P.C., Rhie, A., et al. (2021). The structure, function and evolution of a complete human chromosome 8. Nature *593*, 101–107. https://doi.org/10.1038/s41586-021-03420-7.
15. Altemose, N., Logsdon, G.A., Bzikadze, A.V., Sidhwani, P., Langley, S.A., Caldas, G.V., Hoyt, S.J., Uralsky, L., Ryabov, F.D., Shew, C.J., et al. (2022). Complete genomic and epigenetic maps of human centromeres. Science *376*, eabl4178. https://doi.org/10.1126/science.abl4178.
16. Gershman, A., Sauria, M.E.G., Guitart, X., Vollger, M.R., Hook, P.W., Hoyt, S.J., Jain, M., Shumate, A., Razaghi, R., Koren, S., et al. (2022). Epigenetic patterns in a complete human genome. Science *376*, eabj5089. https://doi.org/10.1126/science.abj5089.
17. Miga, K.H., Koren, S., Rhie, A., Vollger, M.R., Gershman, A., Bzikadze, A., Brooks, S., Howe, E., Porubsky, D., Logsdon, G.A., et al. (2020). Telomere-to-telomere assembly of a complete human X chromosome. Nature *585*, 79–84. https://doi.org/10.1038/s41586-020-2547-7.
18. Willard, H.F., and Waye, J.S. (1987). Chromosome-specific subsets of human alpha satellite DNA: analysis of sequence divergence within and between chromosomal subsets and evidence for an ancestral pentameric repeat. J. Mol. Evol. *25*, 207–214. https://doi.org/10.1007/bf02100014.
19. Gao, S., Oshima, K.K., Chuang, S.-C., Loftus, M., Potapova, T.A., Montanari, A., Gordon, D.S., and Yang, Z. (2026). Human Genome Structural Variation Consortium, Human Pangenome Reference Consortium, et al. A global view of human centromere variation and evolution. Nature. https://doi.org/10.1038/s41586-026-10841-9.
20. Altemose, N., Maslan, A., Smith, O.K., Sundararajan, K., Brown, R.R., Mishra, R., Detweiler, A.M., Neff, N., Miga, K.H., Straight, A.F., and Streets, A. (2022). DiMeLo-seq: a long-read, single-molecule method for mapping protein-DNA interactions genome wide. Nat. Methods *19*, 711–723. https://doi.org/10.1038/s41592-022-01475-6.
21. Hansen, N.F., Dwarshuis, N., Ji, H.J., Rhie, A., Loucks, H., Logsdon, G.A., Vollger, M.R., Storer, J.M., Kim, J., Adam, E., et al. (2026). A complete diploid human genome benchmark for personalized genomics. Cell *189*, 4857–4875.e31. https://doi.org/10.1016/j.cell.2026.06.016.
22. Zook, J.M., McDaniel, J., Olson, N.D., Wagner, J., Parikh, H., Heaton, H., Irvine, S.A., Trigg, L., Truty, R., McLean, C.Y., et al. (2019). An open resource for accurately benchmarking small variant and reference calls. Nat. Biotechnol. *37*, 561–566. https://doi.org/10.1038/s41587-019-0074-6.
23. Zook, J.M., Catoe, D., McDaniel, J., Vang, L., Spies, N., Sidow, A., Weng, Z., Liu, Y., Mason, C.E., Alexander, N., et al. (2016). Extensive sequencing of seven human genomes to characterize benchmark reference materials. Sci. Data *3*, 160025. https://doi.org/10.1038/sdata.2016.25.
24. Scheinfeldt, L., Pompetti, A., Calendo, G., Pozner, T., Grandizio, C., Smith, G., Hodges, K., Gharani, N., Kusic, D., Mitchell, M., and Turan, N. (2025). New iPSC resource with long-read whole genome sequencing characterizations for enhanced in vitro modeling. Preprint at bioRxiv. https://doi.org/10.1101/2025.06.17.660113.
25. Lehnertz, B., Ueda, Y., Derijck, A.A.H.A., Braunschweig, U., Perez-Burgos, L., Kubicek, S., Chen, T., Li, E., Jenuwein, T., and Peters, A.H.F.M. (2003). Suv39h-mediated histone H3 lysine 9 methylation directs DNA methylation to major satellite repeats at pericentric heterochromatin. Curr. Biol. *13*, 1192–1200. https://doi.org/10.1016/s0960-9822(03)00432-9.
26. Sidhwani, P., Schwartz, J.P., Fryer, K.A., and Straight, A.F. (2025). Histone H3 lysine methyltransferase activities control compartmentalization of human centromeres. Preprint at bioRxiv. https://doi.org/10.1101/2025.07.01.662447.
27. Sweeten, A.P., Schatz, M.C., and Phillippy, A.M. (2024). ModDotPlot-rapid and interactive visualization of tandem repeats. Bioinformatics *40*, btae493. https://doi.org/10.1093/bioinformatics/btae493.
28. Cechova, M., Potapova, T.A., Rechtsteiner, A., Hickey, G., Mari, R.S., Mastoras, M., Menendez, J., Poláková, N., Hebbar, P., Ryabov, F., et al. (2025). Complete genomes of a multi-generational pedigree to expand studies of genetic and epigenetic inheritance. Preprint at bioRxiv. https://doi.org/10.64898/2025.12.14.693655.
29. Gamba, R., Mazzucco, G., Wilhelm, T., Velikovsky, L., Salinas-Luypaert, C., Chardon, F., Picotto, J., Bohec, M., Baulande, S., Doksani, Y., and Fachinetti, D. (2022). Enrichment of centromeric DNA from human cells. PLoS Genet. *18*, e1010306. https://doi.org/10.1371/journal.pgen.1010306.
30. Payne, A., Holmes, N., Clarke, T., Munro, R., Debebe, B.J., and Loose, M. (2021). Readfish enables targeted nanopore sequencing of gigabase-sized genomes. Nat. Biotechnol. *39*, 442–450. https://doi.org/10.1038/s41587-020-00746-x.
31. Robinson, J.T., Thorvaldsdóttir, H., Winckler, W., Guttman, M., Lander, E.S., Getz, G., and Mesirov, J.P. (2011). Integrative genomics viewer. Nat. Biotechnol. *29*, 24–26. https://doi.org/10.1038/nbt.1754.
32. Carty, B.L., Dubocanin, D., Murillo-Pineda, M., Dumont, M., Volpe, E., Mikulski, P., Humes, J., Whittingham, O., Fachinetti, D., Giunta, S., et al. (2026). Heterochromatin boundaries maintain centromere position, size and number. Nat. Struct. Mol. Biol. *33*, 220–234. https://doi.org/10.1038/s41594-025-01706-2.

33. Stergachis, A.B., Debo, B.M., Haugen, E., Churchman, L.S., and Stamatoyannopoulos, J.A. (2020). Single-molecule regulatory architectures captured by chromatin fiber sequencing. Science *368*, 1449–1454. https://doi.org/10.1126/science.aaz1646.

34. Dubocanin, D., Hartley, G.A., Sedeño Cortés, A.E., Mao, Y., Hedouin, S., Ranchalis, J., Agarwal, A., Logsdon, G.A., Munson, K.M., Real, T., et al. (2025). Conservation of dichromatin organization along regional centromeres. Cell Genom. *5*, 100819. https://doi.org/10.1016/j.xgen.2025.100819.

35. Tachiwana, H., Kagawa, W., Shiga, T., Osakabe, A., Miya, Y., Saito, K., Hayashi-Takanaka, Y., Oda, T., Sato, M., Park, S.-Y., et al. (2011). Crystal structure of the human centromeric nucleosome containing CENP-A. Nature *476*, 232–235. https://doi.org/10.1038/nature10258.

36. Bodor, D.L., Mata, J.F., Sergeev, M., David, A.F., Salimian, K.J., Panchenko, T., Cleveland, D.W., Black, B.E., Shah, J.V., and Jansen, L.E. (2014). The quantitative architecture of centromeric chromatin. eLife *3*, e02137. https://doi.org/10.7554/eLife.02137.

37. Grafodatskaya, D., Choufani, S., Ferreira, J.C., Butcher, D.T., Lou, Y., Zhao, C., Scherer, S.W., and Weksberg, R. (2010). EBV transformation and cell culturing destabilizes DNA methylation in human lymphoblastoid cell lines. Genomics *95*, 73–83. https://doi.org/10.1016/j.ygeno.2009.12.001.

38. Mahlke, M.A., Lumerman, L., Ly, P., and Nechemia-Arbely, Y. (2023). Epigenetic centromere identity is precisely maintained through DNA replication but is uniquely specified among human cells. Life Sci. Alliance *6*, e202201807. https://doi.org/10.26508/lsa.202201807.

39. Purgato, S., Belloni, E., Piras, F.M., Zoli, M., Badiale, C., Cerutti, F., Mazzagatti, A., Perini, G., Della Valle, G., Nergadze, S.G., et al. (2015). Centromere sliding on a mammalian chromosome. Chromosoma *124*, 277–287. https://doi.org/10.1007/s00412-014-0493-6.

40. Cappelletti, E., Piras, F.M., Biundo, M., Bellone, R.R., Finno, C.J., Kalbfleisch, T.S., Petersen, J.L., Nergadze, S.G., and Giulotto, E. (2025). CENP-A and centromere evolution in equids. Chromosome Res. *33*, 13. https://doi.org/10.1007/s10577-025-09773-3.

41. Nergadze, S.G., Piras, F.M., Gamba, R., Corbo, M., Cerutti, F., McCarter, J.G.W., Cappelletti, E., Gozzo, F., Harman, R.M., Antczak, D.F., et al. (2018). Birth, evolution, and transmission of satellite-free mammalian centromeric domains. Genome Res. *28*, 789–799. https://doi.org/10.1101/gr.231159.117.

42. Cappelletti, E., Piras, F.M., Sola, L., Santagostino, M., Petersen, J.L., Bellone, R.R., Finno, C.J., Peng, S., Kalbfleisch, T.S., Bailey, E., et al. (2023). The localization of centromere protein A is conserved among tissues. Commun. Biol. *6*, 963. https://doi.org/10.1038/s42003-023-05335-7.

43. Mahlke, M.A., Lumerman, L., Nath, P., Chittenden, C., Hoyt, S., Koeppel, J., Xu, Y., Raphael, R., Zaffina, K., Hook, P.W., et al. (2025). Evolution and instability of human centromeres are accelerated by heterochromatin boundary loss and CENP-A overexpression. Preprint at bioRxiv. https://doi.org/10.1101/2025.02.03.636285.

44. Salinas-Luypaert, C., Dubocanin, D., Lee, R.J., Andrade Ruiz, L., Gamba, R., Grison, M., Velikovsky, L., Angrisani, A., Scelfo, A., Xu, Y., et al. (2025). DNA methylation influences human centromere positioning and function. Nat. Genet. *57*, 2509–2521. https://doi.org/10.1038/s41588-025-02324-w.

45. Tullius, T.W., Heuer, R.A., Bohaczuk, S.C., Mallory, B., Dubocanin, D., Ranchalis, J., Ayaz, A., Mason, C.E., Seli, E., Phillippy, A.M., et al. (2025). Protamine lacunae preserve the paternal chromatin landscape in sperm. Preprint at bioRxiv. https://doi.org/10.1101/2025.10.03.680364.

46. Wong, N.C., Wong, L.H., Quach, J.M., Canham, P., Craig, J.M., Song, J.Z., Clark, S.J., and Choo, K.H.A. (2006). Permissive transcriptional activity at the centromere through pockets of DNA hypomethylation. PLoS Genet. *2*, e17. https://doi.org/10.1371/journal.pgen.0020017.

47. Milagre, I., Pereira, C., Oliveira, R.A., and Jansen, L.E.T. (2020). Reprogramming of human cells to pluripotency induces CENP-A chromatin depletion. Open Biol. *10*, 200227. https://doi.org/10.1098/rsob.200227.

48. Edgar, R.C. (2004). MUSCLE: multiple sequence alignment with high accuracy and high throughput. Nucleic Acids Res. *32*, 1792–1797. https://doi.org/10.1093/nar/gkh340.

49. Smit, AFA, Hubley, R & Green, P. RepeatMasker. http://www.repeatmasker.org.

50. Altemose, N., Miga, K.H., Maggioni, M., and Willard, H.F. (2014). Genomic characterization of large heterochromatic gaps in the human genome assembly. PLoS Comput. Biol. *10*, e1003628. https://doi.org/10.1371/journal.pcbi.1003628.

51. Jain, C., Rhie, A., Hansen, N.F., Koren, S., and Phillippy, A.M. (2022). Long-read mapping to repetitive reference sequences using Winnowmap2. Nat. Methods *19*, 705–710. https://doi.org/10.1038/s41592-022-01457-8.

52. Smirnov, E., Chmúrčiaková, N., Liška, F., Bažantová, P., and Cmarko, D. (2021). Variability of Human rDNA. Cells *10*, 196. https://doi.org/10.3390/cells10020196.

53. Milhaven, M., and Pfeifer, S.P. (2023). Performance evaluation of six popular short-read simulators. Heredity *130*, 55–63. https://doi.org/10.1038/s41437-022-00577-3.

54. Li, H. (2018). Minimap2: pairwise alignment for nucleotide sequences. Bioinformatics *34*, 3094–3100. https://doi.org/10.1093/bioinformatics/bty191.

55. Jain, C., Rhie, A., Zhang, H., Chu, C., Walenz, B.P., Koren, S., and Phillippy, A.M. (2020). Weighted minimizer sampling improves long read mapping. Bioinformatics *36*, i111–i118. https://doi.org/10.1093/bioinformatics/btaa435.

56. Liao, W.-W., Asri, M., Ebler, J., Doerr, D., Haukness, M., Hickey, G., Lu, S., Lucas, J.K., Monlong, J., Abel, H.J., et al. (2023). A draft human pangenome reference. Nature *617*, 312–324. https://doi.org/10.1038/s41586-023-05896-x.

57. Jha, A., Bohaczuk, S.C., Mao, Y., Ranchalis, J., Mallory, B.J., Min, A.T., Hamm, M.O., Swanson, E., Dubocanin, D., Finkbeiner, C., et al. (2024). DNA-m6A calling and integrated long-read epigenetic and genetic analysis with fibertools. Genome Res. *34*, 1976–1986. https://doi.org/10.1101/gr.279095.124.

STAR★METHODS

KEY RESOURCES TABLE

REAGENT or RESOURCE	SOURCE	IDENTIFIER
**Antibodies**		
Anti-Histone H3 (tri methyl K9) antibody - ChIP Grade	Abcam	Cat# ab8898, RRID: AB_306848
Anti-CENP-C (Human) pAb	MBL Life Science	Cat# PD030, RRID: AB_10693556
CENP-A monoclonal antibody (3–19)	Enzo Life Sciences	Cat# ADI-KAM-CC006, RRID: AB_10621614
prhaBAD-*anti*-mouse-Nb-Hia5	Addgene	RRID: Addgene_239626
pA-Hia5	Addgene	RRID: Addgene_174372
**Biological samples**		
HG002 lymphoblastoid cells (GM24385)	Coriell Institute	RRID: CVCL_1C78
HG002 iPSC from Fibroblast (GM27730)	Coriell Institute	RRID: CVCL_ZH94
HG002 iPSC from LCL (GM26105)	Coriell Institute	RRID: CVCL_LP11
**Chemicals, peptides, and recombinant proteins**		
HEPES-KOH 1M pH 7.5	Boston BioProducts	BBH-75-K
NaCl 5 Molar	Sigma-Aldrich	59222C-500ML
Spermidine 6.4M	Sigma-Aldrich	S0266-5G
Roche cOmplete^{TM} EDTA-free Protease Inhibitor Tablet	Sigma-Aldrich	11873580001
Bovine Serum Albumin	Sigma-Aldrich	A6003-25G
Digitonin	Sigma-Aldrich	300410-250MG
Tween 20	Sigma-Aldrich	P7949-100ML
KCl	Sigma-Aldrich	PX1405-1
EDTA 0.5 Molarity pH 8	Invitrogen	15575-038
EGTA 0.5 Molarity pH 8	Fisher	50-255-956
S-Adenosylmethionine 32 Milimolar	NEB	B9003S
PFA, 16%	EMS	15710
Glycine	Fisher	BP381-1
Trypan Blue	Fisher	T10282
RNAse cocktail	Thermo	AM2286
Phosphate Buffered Saline	Gibco	10010-023
Tris-HCL pH 8.0 1M	Invitrogen	15568-025
Isopropanol	Fisher	BP2618
Ethanol	Fisher	BP2818
RPMI 1640*	Gibco	21875034
Glutamax	Gibco	35050061
Human FGF-basic	Gibco	13256-029
Tissue culture flask (vented lid) – equipment	Cell Treat	229341
RPMI-1640	Gibco	11875093
mTeSR^{TM}1	Stem Cell Technologies	85850
DMEM/F-12	Gibco	11320033
KnockOut Serum Replacement	Gibco	10828028
DMSO [10%]	Sigma Aldrich	D8418
Fetal Bovine Serum	Gibco	27140079
2mL Freezing Vials	Corning	430659
Versene Solution	Gibco	15040066

(*Continued on next page*)

Continued		
REAGENT or RESOURCE	SOURCE	IDENTIFIER
TrypLE Express	Gibco	12604013
Penicillin-streptomycin	Gibco	15070063
Cell Freezing Medium-DMSO Serum free 1×	Sigma-Aldrich	C6295
Critical commercial assays		
Qubit dsDNA BR Assay Kit	Fisher	Q32850
Qubit Protein Assay Kit	Fisher	Q33211
Deposited data		
Sequencing data submission	This paper	SRA: PRJNA1451277
Publicly available HG002 sequencing data used in this study	Genome-in-a-Bottle (GIAB); NCBI SRA	SRA: SRX14226557
Publicly available HG002 sequencing data used in this study	Genome-in-a-Bottle (GIAB); NCBI SRA	SRA: SRX14226558
Software and algorithms		
IGV (Integrative Genomics Viewer) v2.x	Broad Institute	https://software.broadinstitute.org/software/igv/
Dorado v0.6.1	Oxford Nanopore Technologies	https://github.com/nanoporetech/dorado
Winnowmap v2.03	Marbl	https://github.com/marbl/Winnowmap
modkit v0.4.1	Oxford Nanopore Technologies	https://github.com/nanoporetech/modkit
fibertools v0.5.4	University of Washington	https://github.com/fiberseq/fibertools-rs
samtools	Wellcome Sanger Institute	http://www.htslib.org/
bedtools	University of Utah	https://bedtools.readthedocs.io
HDBSCAN	GitHub (McInnes et al.)	https://github.com/scikit-learn-contrib/hdbscan
NumPy	GitHub (Harris et al.)	https://numpy.org
SciPy	GitHub (Virtanen et al.)	https://scipy.org
Pandas	The Pandas Development Team	https://pandas.pydata.org
Matplotlib	GitHub (Hunter et al.)	https://matplotlib.org
ModDotPlot	Sweeten et al.[27]	https://github.com/marbl/ModDotPlot
muscle3	Edgar et al.[48]	https://drive5.com/muscle
RepeatMasker	Smit et al.[49]	https://www.repeatmasker.org
horhap_tool	GitHub (Ryabov et al.)	https://github.com/fedorrik/horhap_tool
superHOR annotation notebook	GitHub (Ryabov et al.)	https://github.com/fedorrik/superHOR_HG002
Code availability		
Analysis code	This paper	Zenodo: 10.5281/zenodo.20214995
Other		
Eppendorf DNA LoBind tubes (1.5 mL, 5mL)	Eppendorf	022431021, 0030108310
Wide bore 200 µL and 1000 µL pipette tips	Mettler Toledo	30389241, 30389218
Flow cells	Oxford Nanopore	FLO-PRO114M
Monarch HMW DNA Extraction Kit for tissue	NEB	T3060
Ultra-Long DNA Sequencing Kit V14	Oxford Nanopore Technologies	SQK-ULK114
Flow Cell Wash Kit	Oxford Nanopore Technologies	EXP-WSH004

EXPERIMENTAL MODEL AND STUDY PARTICIPANT DETAILS

Cell lines

GM24385, GM26105, and GM27730

All cell lines used in this study are human (Homo sapiens) and derived from a single donor (n = 1; HG002/Personal Genome Project participant huAA53E0). Coriell reports the donor as male, White, and 45 years of age at the time of sampling. The GM26105 and GM27730 induced pluripotent stem cell (iPSC) lines have a reported 46,XY karyotype. GM24385 is an Epstein–Barr virus-transformed B-lymphocyte cell line; GM26105 is an iPSC line derived from the GM24385 line; and GM27730 is an iPSC line derived from fibroblasts. Gender identity, ancestry beyond the reported race, ethnicity, and socioeconomic status are not reported in the

Coriell catalog records and were therefore unavailable to the investigators. The absence of these participant characteristics and the use of cell lines derived from a single donor represent limitations on the generalizability of these findings across diverse human populations. Species identity was confirmed by Coriell using a LINE assay. No additional STR-based authentication was performed in this study; lines were used as distributed by the Coriell Institute. The absence of STR-based authentication represents a limitation for independent verification of cell line identity.

Influence of sex and gender on results

The donor was reported by Coriell as male, and the GM26105 and GM27730 cell lines have a 46,XY karyotype. Gender identity was not reported and could not be evaluated. No sex- or gender-based analyses were performed, because all cell lines were derived from a single donor and therefore did not permit comparisons across sex or gender groups. This limits the generalizability of findings to cell lines derived from individuals of other sexes, gender identities, or sex chromosome complements, including XX-derived models, and represents a limitation of the current study.

Ethical oversight

The cell lines used in this study were obtained from the Coriell Institute for Medical Research through the NIGMS Human Genetic Cell Repository. Coriell identifies these samples as Personal Genome Project Institutional Guidance on Informed Consent (PIGI)-consented samples. The donor provided informed consent for research use through the source repository. The investigators did not prospectively recruit human participants or obtain new human biospecimens, and no identifying donor information was accessible to the investigators. Research involving human induced pluripotent stem cell (hiPSC) lines GM26105 and GM27730 was conducted in compliance with the ISSCR 2021 Guidelines for Stem Cell Research and Clinical Translation as routine pluripotent stem cell research. Both lines were originally derived by the Coriell Institute under informed consent permitting research use, including differentiation and developmental modeling. No new embryos or gametes were created or used.

METHOD DETAILS

Centromere satellite annotation tool suite

Centromere satellite ('CenSat') annotation track

Centromeric and pericentromeric satellites were annotated using the CenSat workflow. The analysis code and modified implementations of the annotation tools described below are provided together in a single Zenodo software package (Zenodo: 10.5281/zenodo.20214994). Alpha-satellite (αSat) sequences were annotated using a modified implementation of HumAS-HMMER, which identifies αSat monomers using hidden Markov models (HMMs) representing known higher-order repeats (HORs) and suprachromosomal families (SFs). Monomer annotations were sorted and consolidated into broad αSat classes comprising active centromeric HOR arrays, inactive pericentromeric HORs, divergent HORs (dHORs), and monomeric αSat regions. Classical satellites (HSATII and HSATIII) were annotated using the k-*mer*-based approach described,[50] which uses a database of human classical satellite-specific k-mers. Additional pericentromeric satellite families, including HSat1A, HSat1B, beta satellite, gamma satellite, SST1, SATR, and ACRO sequences, were annotated using RepeatMasker.[49] Ribosomal DNA (rDNA) arrays were identified using HMMs generated from the first and last 700 bp of the human rDNA repeat unit (GenBank: U13369.1)[51] and sequences corresponding to the 18S (RefSeq: XR_007084227.1) and 5.8S (RefSeq: XR_007084259.1)[52] rRNA genes. Annotations were integrated to generate a comprehensive map of centromere-associated satellites and tandem repeats. Overlapping annotations were resolved, satellite arrays <2 kb were removed, and adjacent annotations of the same class were merged. Centromere transition (CT) regions were subsequently defined based on their proximity to centromeric satellites by applying a 2 Mb BEDTools merge from the active αSat array through adjacent regions containing pericentromeric satellite annotations.

Higher-order repeat haplotype (HORhap) analysis

Within active αSat HOR arrays, individual HOR units accumulate sequence variants that distinguish subsets of otherwise closely related repeats. Groups of HOR units sharing characteristic sequence variants were defined as HOR haplotypes (HORhaps). To characterize haplotype-level variation within αSat HOR arrays, we developed a three-stage annotation pipeline integrating HOR monomer annotation, HOR structural variant (StV) identification, and sequence-based clustering of HOR copies. This approach accommodates HOR StVs with distinct monomer compositions, including full-length, deletion, and insertion variants. The pipeline was applied to both homologs of each HG002 chromosome and provides a framework for HORhap annotation in population-scale assemblies. Software and associated tools are available through Zenodo (https://doi.org/10.5281/zenodo.20214994).

HOR monomers were annotated using HumAS-HMMER with the AS-HORs HMM profile database. Each annotation specifies the HOR family (e.g., S2C9H1L) and the position of the monomer within the HOR unit (e.g., S2C9H1L.2). In the second stage, StVs within active αSat arrays were identified from monomer annotations using stv_tool (Table S9). Consecutive monomers were merged into HOR units, such that each annotation represented a full-length HOR (e.g., S2C9H1L.1-7) or an StV with an altered monomer composition. For example, S2C9H1L.1-3_6–7 denotes deletion of monomers 4 and 5. Variants containing insertions were represented using analogous notation. StV annotations were used as input to horhap_tool, and both homologs of each chromosome were analyzed jointly. HOR sequences corresponding to the two most abundant StVs were retrieved for sequence-based HORhap classification.

If the second most abundant StV represented <1% of HOR copies, only the predominant StV was included. Conversely, if the third most abundant StV represented >14% of HOR copies, the three most abundant StVs were included. Chromosome 19 was excluded from HORhap analysis because of its complex StV composition.

Selected HOR sequences were aligned using MUSCLE3.[53] When three StVs were included, sequences from each StV class were aligned separately together with a full-length HOR reference to minimize erroneous alignment of nonhomologous monomers and inappropriate colocalization of distinct monomer deletions. The three resulting alignments were subsequently merged into a single multiple sequence alignment.

Pairwise Hamming distances were calculated across aligned HOR sequences, followed by hierarchical clustering using Ward's linkage. HOR copies were partitioned into k clusters, with k values from 2 to 9 evaluated for each chromosome pair. BED files and alignment visualizations were generated for each value of k, and a single clustering solution was selected for each chromosome by inspection. The resulting HORhap assignment was appended to the HOR annotation (e.g., S2C9H1L.1-7, where C2 denotes HORhap class 2). HORhap-assigned FASTA sequences can subsequently be used to train HMM profiles for rapid annotation of additional assemblies with HumAS-HMMER, without *de novo* HORhap inference. This HMM-based annotation step was not required here because both HG002 homologs were included directly in the HORhap analysis.

HORhap age analysis

Previous analyses of CHM13 centromeres suggested that kinetochore-associated regions preferentially overlap more recently expanded HORhap arrays.[15] To assess whether this relationship was also observed in HG002, we estimated the relative age of HORhaps based on sequence divergence among HOR copies. Mean pairwise divergence was calculated among HOR units assigned to each HORhap, with lower divergence interpreted as evidence of more recent expansion. Phylogenetic trees constructed from HORhap consensus sequences were additionally used to assess relationships among HORhaps and inform relative age assignments. HORhap tracks were annotated according to the inferred relative age of each HORhap. HORhap annotations were intersected with CDR coordinates to determine whether CDRs overlapped relatively younger or older HORhaps. CDRs overlapped younger HORhaps on 28 chromosomes and older HORhaps on 9 chromosomes (Table S10). Nine chromosomes for which HORhaps could not be clearly distinguished were excluded from this analysis. Although CDRs more frequently overlapped younger HORhaps, consistent with the trend previously reported for CHM13 centromeres, this association was reduced after accounting for the relative aggregate span of younger and older HORhap arrays. A binomial test did not reject the null hypothesis of no preferential CDR association with younger HORhaps ($p = 0.092$) (Table S10). P-values were calculated in Python using SciPy (scipy.stats.binomtest(28, 37, 0.64, alternative="greater")).

SuperHOR annotation

To investigate whether HOR units form higher-order repetitive structures beyond the standard HOR periodicity, we produced a superHOR annotation for HG002. HORhap annotations were scanned for n-mers of HORs (n = 2–9) repeated at least three times consecutively. Each "unit" in the n-mer is itself a HOR, and at least one HOR in an n-mer should have a HORhap assignment different from the others. Identified superHOR arrays are colored by their *n* value, providing an immediate visual indication of the periodicity in each superHOR array.

Subfamily characterization of human satellites

Arrays annotated as HSat2,3 in the CenSat annotation for T2T-HG002 were further categorized into the 3 HSat2 subfamilies and 11 HSat3 subfamilies previously identified.[50] HuRef reads assigned to each subfamily were aligned to the T2T-HG002 reference using Winnowmap (k = 15).[51]

Local identity calculation

Active arrays from the hg002v1.1 assembly were identified in the CenSat v2.0 BED file by selecting entries ending with "H1L". Adjacent intervals (within 200 kb) were merged using bedtools. FASTA sequences for each active array were extracted with samtools and input to ModDotPlot[27] using a 5 kb window size and delta = 0. The resultant bed files, with all-vs-all identities, were processed using a custom Python script that averages the identity values for each region's closest neighbors (default of two regions on each side). Averaged values were assigned to a color scale determined either by a fixed minimum identity threshold (90%) or by the 10th percentile of observed values, and the results were written to BED files.

Centromere mappability assessment

Accurate genome assembly of human centromeres remains a fundamental challenge due to the highly repetitive nature of centromeric satellite DNA, including alpha satellites organized into higher-order repeats (HORs) and human satellite sequences (HSat1–3). Existing mappability tools were developed for short-read sequencing and have not been validated on fully assembled centromeric sequences. Moreover, they do not capture the mappability characteristics of long reads, which are now central to efforts to resolve previously intractable genomic regions. Here, we describe a method for generating long-read centromere mappability tracks and evaluate the performance of two state-of-the-art aligners, Minimap2[54] and Winnowmap,[55] for confidently mapping reads to these regions.

To establish a ground truth for read alignment performance, we simulated error-free reads from the centromeric regions of the T2T-HG002 (HG002v1.0.1) reference genome.[21] Centromeric regions were defined by the coordinates of the active HOR array for each chromosome, expanded by 5 Mb on both sides to capture flanking satellite sequences and transition regions. Using the read simulator wgsim,[53] we generated 10,000 error-free reads per chromosome at eleven read sizes ranging from 1 kb to 300 kb, as well as 30× coverage simulations for each read size. Reads were simulated with 0% error rate to eliminate sequencing noise as a confounding variable, enabling a clean assessment of how genomic sequence complexity alone affects alignment accuracy. Simulated reads were aligned to the full diploid HG002 reference genome using both Minimap2 v2.26 and Winnowmap v2.03 under ONT parameters (map-ont). Each aligned read was classified into one of six categories: (1) uniquely mapped reads with a single, perfect alignment to the correct haplotype; (2) multi-mapping reads with one top alignment to the correct haplotype; (3) multi-mapping reads with two top alignments both to the correct haplotype; (4) multi-mapping reads with two top alignments to different haplotypes, or three or more top alignments; (5) reads mapping to an incorrect chromosome; and (6) unmapped reads. A "top alignment" was defined by a perfect CIGAR string, zero edit distance (NM:i:0), and an alignment score equal to twice the read length, strict criteria appropriate for error-free simulated reads.

Mappability tracks were generated in BED format by recording genomic intervals covered by uniquely mapped reads at each read size. These tracks reveal that mappability increases substantially with read length. Active HOR arrays, which are the functionally critical regions of the centromere where CENP-A binds to facilitate kinetochore assembly, become fully mappable at read sizes of 40–60 kb for most chromosomes, indicating that these arrays are largely haplotype-specific. Diverged and monomeric HOR regions remain largely unmappable until read sizes of 200 kb or greater, consistent with their higher degree of sequence similarity across haplotypes and homologous chromosomes. Human satellite regions, including HSat1A and portions of HSat2, also become uniquely mappable at 60 kb, suggesting these sequences are sufficiently haplotype-specific to support confident alignment at ultra-long read lengths. Notably, non-centromeric flanking regions showed few mappable intervals despite their presumed lower repetitiveness, likely because reads originating from these regions align preferentially to other haplotypic copies as non-primary alignments. These mappability tracks provide a practical resource for filtering variant calls and assembly evaluations to regions of high alignment confidence.

Quality control and characterization of hiPSC lines

Initial genomic characterization of GM26105 and GM27730, including short-read whole-genome sequencing, karyotyping, pluripotency marker validation, and confirmation of high single-nucleotide variant (SNV) and small indel (<50 bp) concordance with the donor reference, was previously described.[24] Here we report additional quality control analyses performed specifically to support the centromere-focused studies described in the main text, including long-read nanopore sequencing, structural variant and genome integrity assessment, CDR calling, and long-read RNA expression profiling of CENP-A.

Human iPSC lines were cultured as previously described,[24] without antibiotics or antifungals in mTeSR1 medium (STEMCELL Technologies) and maintained at 37°C with 5% CO2 and ambient oxygen levels on Matrigel-coated tissue culture vessels. Cells were passaged when reaching approximately 75–85% confluency at a split ratio of 1:3–1:6. Subcultivation was performed using standard dissociation reagents (Versene or ReLeSR), and cells were reseeded in fresh Matrigel-coated vessels with mTeSR1 medium. Medium was replaced daily to maintain optimal culture conditions. Cell density and morphology were monitored routinely to ensure healthy proliferation and the absence of contamination. For cryopreservation, cells were frozen in a medium composed of 90% KnockOut Serum Replacement and 10% DMSO.

To enable assessment of structural variation and centromere-level genome integrity, we generated Oxford Nanopore Technology (ONT) ultra-long (UL) whole-genome sequencing (WGS) data from GM26105 and GM27730. DiMeLo-seq[20] was performed on the PromethION platform (PromethION, R10.4.1) using an ultra-long read protocol optimized for high-molecular-weight DNA isolation and library preparation, for both GM26105 and GM27730. In this experiment, we utilized a modified partial ultra-long method, where we added a step of shearing the DNA by pipetting with a standard P1000 pipette tip. This step was performed until no viscosity was observed in the samples. Furthermore, during the fragmentation mix step after the addition of fragmentase, we utilized the standard P1000 pipette tips instead of the widebore P1000 tips. Both experiments achieved read length N50s of 108,349 bp and 96,890 bp, respectively. Raw signal data were basecalled using Dorado v1.0.2 with modified base detection enabled for 5-methylcytosine (5mC) and N6-methyladenine (6mA) using dna_r10.4.1_e8.2_400bps_sup@v5.2.0. The coverage for GM26105 was 9.4812, and the average coverage for GM27730 was 16.9247.

Prior QC of these lines[24] confirmed high concordance of SNVs and small indels relative to the GIAB HG002 reference callset; however, structural variants (SVs) and large-scale rearrangements, particularly within centromeric satellite arrays, were not systematically evaluated. These regions are prone to somatic copy number variation and rearrangement during reprogramming and extended culture, and instability in centromeric sequence could confound downstream analyses of centromere identity and chromatin organization. Furthermore, the use of the complete T2T-HG002 assembly as the alignment target introduces unique mapping considerations in centromeric regions not encountered with GRCh38. To assess large-scale genome integrity, we applied Flagger,[56] a reference-based read-depth analysis tool designed to flag regions of unexpected coverage in long-read assemblies and alignments. Flagger partitions the genome into coverage-state categories, including haploid, diploid, collapsed (over-represented), and erroneous (under-represented) windows, by modeling expected read depth distributions and identifying statistically anomalous regions. Critically, Flagger was developed and validated in the context of complete T2T assemblies, where coverage anomalies in centromeric

and segmentally duplicated regions are particularly informative. We focused Flagger analysis on centromeric regions of all 23 chromosome pairs to identify any evidence of copy number change, deletion, or collapse in the two hiPSC lines relative to the donor LCL. Using Flagger v1.1.0, we found that 99.3% of the evaluated CenSat sequence was classified as haploid, with minor fractions flagged as erroneous (0.6%), collapsed (0.1%), or duplicated (0.04%), indicating that centromeric architecture is broadly preserved across both hiPSC lines. Importantly, no assembly errors were reported overlapping with CDR intervals. However, it is possible that sites of decreased methylation (due to read misalignment) may have contributed to the limited false peak calls in the hiPSC that are outside of the primary CDR location.

CDR predictions

Lymphoblastoid cell line CDR prediction

For LCLs, mCpG wiggle tracks were generated from the processed methylation calls and visualized in Integrative Genomics Viewer (IGV)[31] alongside the corresponding raw read-level mCpG signal. Sub-CDRs were manually annotated as contiguous regions in which both the wiggle-track mCpG percentage and the raw read mCpG signal showed a pronounced reduction, typically from an average of approximately 60–40% mCpG down to 25–10%. Intervening regions between sub-CDRs that did not display this marked decrease were excluded from the prediction, and CDR boundaries in LCLs were defined solely on the basis of these mCpG patterns.

hiPSC CDR prediction

Centromere Dip Regions (CDRs) mark sites that have reduced CpG methylation relative to the surrounding active αSat HOR array. The HG002 hiPSC active arrays have higher array methylation (average 87% vs. ~60% for matched LCLs), and present a challenge to predict sub-CDR boundaries, as shown below for the chromosome 10 PATERNAL array (Figure S22A). Therefore, in an effort to define sub-CDRs in the hiPSC 5mC dataset, we developed a sliding-window CDR caller that detects all contiguous regions falling below a per-array threshold, treats each as a distinct candidate domain, and assigns each domain a composite reliability score to distinguish well-supported calls from marginal detections at the detection boundary. CDR calling was performed on phased, per-CpG methylation bedgraph files derived from Oxford Nanopore long-read sequencing. Each file represents a single haplotype of a single alpha-satellite array, with columns: chromosome/haplotype name, CpG start position, CpG end position, and methylation percentage (0–100). Methylation values were generated using DiMeLo-seq or equivalent long-read methylation-aware base-calling pipelines. Each bedgraph covers the full span of the assembled alpha-satellite array for the corresponding haplotype.

For each haplotype array, the mean CpG methylation was computed across all CpG sites in the input bedgraph(Figure S22B). This array-wide mean serves as the baseline methylation level for the array. The CDR detection threshold was set at 10 percentage points below the array-wide (i.e., CDR threshold = array mean methylation −10%). This array-relative threshold accounts for the substantial variation in baseline methylation across centromeric arrays (ranging from approximately 77% on chrY to 96% on chr10) and avoids applying a fixed absolute threshold that would be inappropriately stringent for lower-methylation arrays or permissive for higher-methylation arrays.

Sliding Window Smoothing: A sliding window was applied across each array to smooth the per-CpG methylation signal and reduce the influence of individual CpG-level noise. Windows of 10 kb were stepped at 1 kb intervals across the array coordinates. For each window, the mean methylation of all CpG sites falling within the window boundaries was computed. Windows containing fewer than 3 CpGs were excluded from analysis to avoid unreliable mean estimates in sparsely covered regions.

Domain Calling: CDR domains were identified as contiguous runs of windows whose mean methylation fell below the CDR threshold. Each uninterrupted run of below-threshold windows was treated as a single candidate domain, with its genomic boundaries defined by the positions of the first and last windows in the run. This multi-domain approach explicitly allows for complex CDR architectures containing multiple distinct hypomethylated sub-domains separated by partial methylation recoveries, which are observed on a number of chromosomes. Candidate domains containing no individual CpG observations in the raw bedgraph data (degenerate calls arising from window boundary effects) were discarded.

Domain Reliability Scoring: The sliding window approach can produce candidate domains that are small, shallow, and sit only marginally below the detection threshold. Such calls may reflect local noise in the methylation signal rather than a genuine hypomethylated sub-domain. To distinguish well-supported CDR domains from marginal calls, we assigned each candidate domain a composite reliability score (0–100) based on three orthogonal properties. The reliability score was calculated as a weighted sum of three normalized components: methylation depth (50%), CpG count (30%), and domain span (20%). The depth score quantified the reduction in mean domain methylation relative to the detection threshold and was capped at 1.5 times the threshold offset to limit the influence of extreme values. The CpG score reflected the number of CpG observations supporting each domain and saturated at 100 CpGs, whereas the span score reflected domain length and saturated at 20 kb. Component scores were calculated as (depth_score = \min[(threshold - domain_mean)/threshold_offset, 1.5]/1.5), (cpg_score = \min(nCpG/100, 1.0)), and (span_score = \min(span_kb/20, 1.0)). The composite score was calculated as (0.50 \times depth_score +0.30 \times cpg_score +0.20 \times span_score), scaled to a 0–100 range, and clamped at zero.

Confidence Classification: Domains with a reliability score ≥50 are classified as high confidence; domains with a score <50 are classified as LOW_CONFIDENCE. The threshold of 50 was selected by inspection of the score distributions across all chromosomes: it reliably excluded domains with very small size (<5 kb), very few CpGs (<30), and methylation means within 2–3 percentage points of the threshold, while retaining domains with substantive hypomethylation and adequate CpG support. Consideration of the data provides empirical support for a cutoff at 50. Across all 141 domains called genome-wide, there is a natural gap in the score

distribution: the maximum LOW_CONFIDENCE score is 49.9 and the minimum high confidence score is 50.1, the two classes do not overlap at all. This is not an artifact of the cutoff being imposed; the scoring components (depth, CpG count, span) jointly produce a bimodal distribution where marginal domains cluster in the 0–50 range and well-supported domains cluster from ~50 upward. However, we noted that specific chromosomes, like chromosome 21 MATERNAL are much smaller than the other CDR estimates, and have several domains in the score category of 40–49 (Figure S22C).

CDR Total Span Definition: The total CDR span was defined as the genomic interval from the start of the first high confidence domain to the end of the last high confidence domain for each haplotype. Peripheral low confidence domains were excluded because these domains were occasionally separated from the primary CDR cluster by > 200 kb and substantially inflated the apparent CDR extent. Restricting the span to high confidence domains therefore provided a conservative estimate of CDR extent.

CDRs were identified using cdr_caller_v2.py (included in Zenodo software package Zenodo: https://doi.org/10.5281/zenodo.20214994), implemented in Python 3 with NumPy (v1.24+) and Matplotlib (v3.7+). Unless otherwise specified, CDR calling used a 10 kb sliding window with a 1 kb step, a minimum of three CpGs per window, a detection threshold 10 percentage points below the array-wide mean methylation, and a reliability score cutoff of 50 for high confidence classification.

Fiber-seq

To perform the Fiber-seq experiment,[32] all reagents were freshly prepared and syringe-filtered through a 0.2 μm filter before being kept on ice. Live cells (1M–5M per condition) were pelleted at 300 × *g* for 5 min and washed with PBS.

Pelleted cells were resuspended in 1 mL of Dig-Wash buffer (0.02% digitonin, 20 mM HEPES-KOH, pH 7.5, 150 mM NaCl, 0.5 mM Spermidine, 1 Roche Complete tablet-EDTA (11873580001) per 50 mL buffer, 0.1% BSA) and incubated on ice for 5 min. Note that using detergents other than digitonin and Tween may reduce methylation efficiency. The nuclei suspension was split into separate tubes for each condition and spun down at 4°C at 500 × *g* for 3 min. All subsequent spins were performed under the same conditions, and all steps involving pipetting nuclei were performed with wide bore tips.

The supernatant was removed, and the pellet was gently resuspended in 200 μL of Tween-Wash (0.1% Tween 20, 20 mM HEPES-KOH, pH 7.5, 150 mM NaCl, 0.5 mM Spermidine, 1 Roche Complete tablet-EDTA per 50 mL buffer, 0.1% BSA) containing the primary antibody at a 1:50 dilution. Samples were placed on a rotator at 4°C for 2 h. Nuclei were then pelleted, washed twice with 0.95 mL Tween-Wash, and resuspended in 200 μL of Tween-Wash containing 200 nM Hia5. The concentration of Hia5 was measured using the Qubit Protein Assay Kit (Q33211). For methyltransferase binding, nuclei were placed on a rotator at 4°C for 2 h. Nuclei were then spun down, washed twice with 0.95 mL Tween-Wash, and resuspended in 100 μL of Activation Buffer (15 mM Tris, pH 8.0, 15 mM NaCl, 60 mM KCl, 1 mM EDTA, pH 8.0, 0.5 mM EGTA, pH 8.0, 0.5 mM Spermidine, 0.1% BSA, 800 μM SAM). The nuclei were incubated at 37°C for 1 h before spinning and resuspending in 100 μL of cold PBS.

The extraction process was initiated by thawing the Extraction EB at room temperature, followed by vortexing and placing it on ice. In a separate tube, a pre-mix of 1.8 mL of NEB Monarch® HMW gDNA Tissue Lysis Buffer and 60 μL of Proteinase K was prepared. This pre-mixed lysis buffer was then added to the suspected nuclei from the experiment, followed by slow pipette mixing using a 1 mL wide-bore pipette. The samples were incubated at 56°C for 60 min at 300 rpm, allowing for thorough lysis. After this, protein separation was achieved by adding 900 μL of Protein Separation Solution, followed by mixing with a Hula Mixer and centrifugation at 16,000 × g for 10 min at 4°C. The DNA, present in the upper phase, was carefully aspirated using a wide-bore pipette tip to avoid disturbing the protein phase below. To facilitate DNA precipitation, three glass beads were added, followed by the addition of 2.5 mL of isopropanol and mixing on a rotator mixer at 10 rpm for 20 min. After resting the tubes at room temperature for 1 min, the isopropanol was carefully aspirated, leaving the DNA bound to the glass beads. The samples were then washed twice with 2 mL of Wash Buffer, and the remaining wash buffer was removed by quick soft spinning. Finally, the beads were transferred into a 2 mL tube containing 200 μL of Extraction EB and incubated overnight at room temperature to allow DNA extraction. The DNA was then separated from the beads by spinning, and the final volume was adjusted to 750 μL by adding additional Extraction EB for downstream applications. To begin the data analysis, ONT Fiberseq data was aligned to the hg002v1.1 genome using Winnowmap (v2.03). ONT dorado called MM/ML tags were filtering using modkit (v0.4.1) call-mods to 0.1. As per recommendation from the fibertools book.[57] Nucleosome calls were added after filtering to individual reads using fibertools (v0.5.4) add-nucleosomes. Aggregate 6mA/A fraction modified bedMethyl files were calculated using modkit pileup (v0.4.1). Fibertools (v0.5.4) extract was used to calculate genomic positions of nucleosomes and MSPs for aggregation.

Cell culture and passage

HG002 lymphoblastoid cells (GM24385; Coriell Institute; mycoplasma tested) were maintained in RPMI 1640 (Gibco, 11875093 or 21875034; 2 g/L glucose) supplemented with 2 mM L-glutamine provided as GlutaMAX (Gibco, 35050061), 15% fetal bovine serum (Gibco, 26140079; lot-selected from a reputable source, Southern Hemisphere origin preferred), and 1% penicillin–streptomycin (Gibco, 15070063) at 37°C in a humidified atmosphere of 5% CO2.

HG002 LCL were passaged at a split ratio of 1:3 after growing to a density of 0.9–1.0 × 10^6 viable cells/mL. At each passage, twelve million cells were pelleted at 300 × g for 5 min and resuspended in cell freezing media (Sigma-Aldrich C6295) at a concentration of 5.0 million cells/mL. A Mr. Frosty freezing container (Thermo Scientific, 5100–0001) was used to cryopreserve the harvested cells at −80°C. Ten million cells were cryopreserved for DiMeLo-seq, and two million cells cryopreserved for future culturing.

CellPress
OPEN ACCESS

GM27730 iPSC (Coriell Institute; mycoplasma tested) were maintained in DMEM/F12 supplemented with 20% KnockOut Serum Replacement (KOSR) and 10 ng/mL basic fibroblast growth factor (bFGF) at 37°C in a humidified atmosphere of 5% CO2 on CF1 mouse embryonic fibroblast (MEF) feeders prepared on 0.1% gelatin-coated surfaces. Cells were passaged every 5–7 days at a 1:6 split ratio using TrypLE Express.

GM26105 iPSC (Coriell Institute; mycoplasma tested) were maintained in mTeSR1 at 37°C in a humidified atmosphere of 5% CO2 on Matrigel-coated surfaces. Cells were passaged every 5–7 days at a 1:8 split ratio using Versene and recovered in 2 wells of a 6-well plate.

DiMeLo-seq

All reagents were freshly prepared and syringe-filtered through a 0.2 μm filter before being kept on ice. Cells (1M–5M per condition) were pelleted at 300 × *g* for 5 min and washed with PBS. For experiments targeting CENP-A, H3K9me3, and CENP-C, live cells were used. The standard DiMeLo-seq protocol's[19] nuclear isolation can then be continued.

Pelleted cells were resuspended in 1 mL of Dig-Wash buffer (0.02% digitonin, 20 mM HEPES-KOH, pH 7.5, 150 mM NaCl, 0.5 mM Spermidine, 1 Roche Complete tablet-EDTA (11873580001) per 50 mL buffer, 0.1% BSA) and incubated on ice for 5 min. Note that using detergents other than digitonin and Tween may reduce methylation efficiency. The nuclei suspension was split into separate tubes for each condition and spun down at 4°C at 500 × *g* for 3 min. All subsequent spins were performed under the same conditions, and all steps involving pipetting nuclei were performed with wide bore tips.

The supernatant was removed, and the pellet was gently resolved in 200 μL of Tween-Wash (0.1% Tween 20, 20 mM HEPES-KOH, pH 7.5, 150 mM NaCl, 0.5 mM Spermidine, 1 Roche Complete tablet-EDTA per 50 mL buffer, 0.1% BSA) containing the primary antibody at a 1:50 dilution. An antibody targeting CENP-A (ADI-KAM-CC006-E), H3K9me3 (ab8898), or CENP-C (PD030) was used. Samples were placed on a rotator at 4°C for 2 h. Nuclei were then pelleted, washed twice with 0.95 mL Tween-Wash, and resolved in 200 μL of Tween-Wash containing 200 nM pA-Hia5 for CENP-C and H3K9me3 and anti-mouse nanobody for CENP-A. The concentration of pA-Hia5/anti-mouse nanobody was measured using the Qubit Protein Assay Kit (Q33211). For methyltransferase binding, nuclei were placed on a rotator at 4°C for 2 h. Nuclei were then spun down, washed twice with 0.95 mL Tween-Wash, and resuspended in 100 μL of Activation Buffer (15 mM Tris, pH 8.0, 15 mM NaCl, 60 mM KCl, 1 mM EDTA, pH 8.0, 0.5 mM EGTA, pH 8.0, 0.5 mM Spermidine, 0.1% BSA, 800 μM SAM). The nuclei were incubated at 37°C for 1 h before spinning and resuspending in 100 μL of cold PBS.

DNA extraction

The nuclei were combined with 1.8 mL NEB Monarch HMW gDNA Tissue Lysis Buffer and 60μL proteinase K (800 units/mL) in a 5 mL Eppendorf DNA LoBind tube, and then gently mixed by pipetting with a wide-bore tip. The samples were incubated at 56°C for 60 min at 300 rpm, allowing for thorough lysis. After this, protein separation was achieved by adding 900 μL of Protein Separation Solution, followed by mixing with a rotator mixer for 10 min at 10 rpm and centrifugation at 16,000 × g for 10 min at 4°C. The upper phase containing the DNA was carefully aspirated by pipetting with a wide-bore tip and transferred to a fresh 5 mL Eppendorf DNA LoBind tube.To facilitate DNA precipitation, three NEB Monarch DNA Capture Beads were added, followed by the addition of 2.5 mL of isopropanol and mixing with a rotator mixer at 10 rpm for 20 min. After resting the tubes at room temperature for 1 min, the isopropanol was carefully aspirated, leaving the DNA bound to the glass beads. The glass beads were washed twice and transferred to a bead retainer where any remaining wash buffer was removed by a soft momentary centrifugation. Finally, the beads were transferred to a tube containing 200 μL elution buffer (ONT Extraction EB) and allowed to incubate at room temperature overnight. The DNA was then separated from the beads by centrifugation in a bead retainer for 1 min at 1000 × *g*. The final volume was adjusted to 750μL elution buffer.

ONT ultra-long PromethION library prep and sequencing

In a 1.5 mL Eppendorf DNA LoBind tube, 6 μL fragmentation enzyme (FRA) was diluted with 244 μL dilution buffer (FDB), and mixed by pipetting. The resulting solution was then combined with 750 μL sample DNA and mixed thoroughly by pipetting fifteen times with a wide-bore tip The sample reaction was then incubated at room temperature for 10 min, and placed on ice for 5 min. After cooling, 5 μL rapid adapter (RA) was added to the sample, mixed by pipetting with a wide-bore tip five times, and incubated for 30 min at room temperature. For DNA precipitation, thaw Elution Buffer and Precipitation buffer components, briefly centrifuge, and vortex to mix. Keep all kit components on ice. Following the addition of 500 μL of Precipitation Reagent using a regular pipette tip, we gently rotated the sample on a rotator mixer for 20 min at 10 rpm to promote DNA precipitation. Centrifuge sample at 2000 × *g* for three min. Carefully remove the supernatant, taking care not to aspirate the DNA. Spin down the tube at 2000xg for three minutes and remove any residual supernatant, avoiding DNA aspiration. Add 275 μL of Elution Buffer and incubate overnight at room temperature. Gently mix the DNA library ten times with a P1000 wide-bore pipette tip to prevent sample heterogeneity. For storage, it is recommended to store libraries in Eppendorf DNA LoBind tubes at 4°C for short-term use or reloading flow cells between washes.

PromethION adaptive sampling

For each adaptive sampling run, we provided the PromethION platform with a reference file containing CHM13 coordinates corresponding to mappable regions Table S6. This BED file defines the complement of αSat repeat regions expanded by 100 kb flanks,

excluding easily mappable portions of the ~3.1 Gb T2T-CHM13v2.0 genome while targeting ~190 Mb (6.2%) of pericentromeric and centromeric repeats that are typically refractory to short-read mapping. The PromethION sequencer performed real-time basecalling and mapping of the first few hundred base pairs of each DNA molecule. All samples were sequenced on a PromethION 48 sequencer (PRO-SEQ048) using R10.4.1 Flow Cells (FLO-PRO114M). The SKU wash kits (EXP-WSH004) were used in this process. Critically, we ran the adaptive sampling sequencing run in DEPLETE mode for this bed file. If the DNA molecules mapped to the CHM13 reference coordinates (i.e., mappable euchromatin), they were rejected; otherwise, unmapped molecules, which were enriched for centromeric sequences, were allowed to be fully sequenced. This approach specifically targets centromeres because they comprise long tracts of highly repetitive αSat DNA (2.8% of the genome alone), which evades alignment due to their length, homogeneity, and complexity.

DiMeLo-seq analysis

Basecalling and filtering

Raw Nanopore sequencing data were basecalled from POD5 files using Dorado (Oxford Nanopore Technologies) to simultaneously detect nucleotide sequences and base modifications. Basecalling was performed with the model dna_r10.4.1_e8.2_400bps_sup@v4.2.0, which supports the detection of both 5-methylcytosine and N6-methyladenine. All basecalling was executed on the Phoenix HPC cluster using Dorado v0.6.1 to generate BAM files containing per-base methylation tags for 5mC and 6mA. These unaligned BAM files were then processed using a batch-submitted SLURM script (HG002_dimelo_raw_data_processing; Zenodo: https://doi.org/10.5281/zenodo.20214995) that performs conversion, alignment, and post-processing optimized for long-read Nanopore data. The script converts BAM to FASTQ preserving modification tags, aligns to the HG002 reference using Winnowmap, and generates sorted and indexed primary alignments.

mA visualization and aggregate quantification

Individual reads were visualized using IGV[31] to inspect methylation patterns in the centromere regions. Chromosomal paternal-maternal CENP-A, CENP-C, correlations were visualized as scatterplots (x axis: paternal mA/A density; y axis: maternal mA/A density) with size-difference color-coding. Outliers were identified as chromosomes with standardized residuals $|z| > 3$ from the identity line ($y = x$), where residuals = maternal - paternal density, $z = |(\text{residual} - \mu)/\sigma|$. Furthermore, to visualize the presence of epigenetic marks (CENP-A, CENP-C, and H3K9me3) in chromosomal sub-CDRs, predicted CDR coordination bed files were used to extract reads in those genomic locations. For each chromosome, three metrics were compared between paternal and maternal haplotypes: mA/A density, desired read length, and their product (density × length) as an estimate of integrated CENP-A/C dosage. Pearson correlation coefficients and mean squared error from the identity line ($y = x$) were calculated for each metric. Outlier chromosomes were defined as those with standardized residuals $|z| > 3$, where residuals represent the maternal − paternal difference and z-scores are computed relative to the cross-chromosomal mean and standard deviation of those residuals.

Single-molecule analysis of sub-CDR co-occupancy

Single-molecule 6mA and 5mC densities were calculated for individual reads in CDR and non-CDR active αSat defined regions. For each read overlapping a defined CDR or non-CDR region, the modification score array was extracted from the MM/ML BAM tags and filtered by a minimum quality score threshold (6mA: ≥230/255; 5mC: ≥210/255). non-CDR regions were defined as active αSat intervals 10kb away from the CDRs, tiled into 5,000 bp non-overlapping windows with up to 15 windows retained per chromosome homolog. To identify per-mark density thresholds distinguishing CDR from non-CDR regions, a decision tree algorithm was implemented in Python. Per-read densities from CDR and non-CDR regions were pooled and sorted, and candidate thresholds were evaluated at midpoints between consecutive density values. The threshold minimizing the weighted Gini impurity across the two resulting partitions was selected as optimal. This procedure was applied independently for each epigenetic mark, yielding the following genome-wide thresholds: CENP-A 6mA density: 0.011; CENP-C 6mA density: 0.014; H3K9me3 6mA density: 0.015. These thresholds were used to classify individual reads as CDR-enriched or non-CDR at single-molecule resolution.

Epigenetic comparisons between flanking and central CDRs in early-passage LCLs and late-passage LCLs

All raw reads were visualized in IGV.[31] The 5mC methylation island regions are defined as regions exhibiting CpG methylation enrichment in early-passage LCLs that is absent in late-passage LCLs. To characterize the epigenetic status of these differentially methylated island regions, per-chromosome mean 5mC densities were calculated for center CDR, flanking CDR, and non-CDR baseline regions using single-molecule CpG methylation calls extracted from DiMeLo-seq BAM files. CENP-A and H3K9me3 5mC densities were then normalized to the non-CDR active array baseline mean, and $\log_2$ fold-changes were computed per chromosome. Distributions of these $\log_2$ fold-changes were compared between early- and late-passage conditions for center and flanking CDR subregions using Welch's two-sample t-tests, with effect sizes reported as Cohen's d.

Epigenetic comparisons between differentially methylated regions in early-passage LCLs and late-passage LCLs

To identify differentially methylated regions in-between sub-CDRs, CpG methylation profiles were compared between early- and late-passage LCLs across the chromosomal CDRs. Sub-CDR boundaries were first identified in both passage conditions. Regions exhibiting CpG methylation enrichment in the early-passage LCL that transitioned to a depleted state in the late-passage LCL were

isolated by subtracting the late-passage sub-CDR coordinates from those of the early-passage condition. The resulting intervals (regions present in the early-passage methylation landscape but absent in the late-passage) were marked.

Next, to characterize the epigenetic state of differentially methylated regions, CENP-A, 5mC, and H3K9me3 signal densities were extracted from the differentiated regions. Per-read modification densities were calculated from DiMeLo-seq BAM files, with 6mA calls filtered to a minimum quality of 230/255 and 5mC calls to 230/255. The six resulting density tracks (CENP-A, 5mC, and H3K9me3 for each passage condition) were merged by genomic coordinates. Signal densities within each compartment were then normalized to chromosome-specific non-CDR baseline values computed separately for each mark and passage condition, and $\log_2$ fold-changes were calculated per region. Distributions of $\log_2$ fold-changes were visualized as boxplots with individual chromosome-level data points overlaid, enabling direct comparison of epigenetic signal enrichment or depletion across passage conditions.

Epigenetic comparisons between centromeric differentially methylated regions in early-passage LCLs and iPSCs

To compare CENP-A and CpG methylation enrichment at CDRs between the early-passage LCL and iPSC cell states, per-read 6mA and 5mC densities were extracted from DiMeLo-seq BAM files across active αSat array windows and CDR windows. For each mark, a chromosome-specific background mean density was computed from active αSat windows after excluding any window falling within 50 kb of a CDR interval, ensuring that background estimates reflected chromatin state distal from CDR-proximal regions. CDR window densities were then normalized to this background mean, and $\log_2$ fold-changes were calculated per CDR window. Distributions of log2 fold-changes were compared between the early-passage LCL and iPSC and visualized as boxplots with individual CDR-level data points overlaid.

Single-molecule read clustering based on methylation

CDRs were classified into three categories: edge regions (outermost CDR boundaries), sinking regions (small disappearing sub-domains), and stable island regions (persistently hypomethylated CDR cores), each comprising ten genomic intervals defined in BED format. For each region, CpG dinucleotides were enumerated directly from the reference sequence.

Per-read CpG methylation matrices were constructed by extracting 5mC modification probabilities at each CpG site within a given CDR interval, retaining only reads spanning at least 95% of the region (windowFrac = 0.95). Each matrix was organized with individual reads as rows and CpG positions as columns, with values representing continuous methylation probability scores (0 = unmethylated, 1 = fully methylated), yielding a high-resolution, read-level view of methylation heterogeneity across each CDR.

These per-read methylation matrices were used as input to HDBSCAN (Hierarchical Density-Based Spatial Clustering of Applications with Noise) to identify subpopulations of reads sharing distinct methylation patterns within each CDR. Hierarchical linkage was computed using Ward's minimum variance method, which minimizes within-cluster variance at each merge step and is well-suited to identifying compact, well-separated clusters in continuous data. Clustering parameters were set as follows: minimum cluster size = 5, minimum samples = 3, and cluster selection epsilon = 0.005. Reads assigned to noise (cluster label −1) were excluded from interpretation. Clustering was performed independently for each of the ten regions within each CDR category (edge, sinking, stable), allowing region-specific methylation substructure to be resolved without assumptions about the number of clusters. The resulting cluster assignments were visualized as heatmaps of per-read methylation probability, ordered by cluster membership, with accompanying dendrograms to illustrate hierarchical relationships between read groups.

QUANTIFICATION AND STATISTICAL ANALYSIS

All statistical analyses were performed in Python using the SciPy, NumPy, and Pandas libraries. Statistical details for each comparison are reported in the corresponding figure legends.

Dosage analysis

To assess allele-specific CENP-A dosage, mean signal density and total CDR length in bp were computed separately for paternal and maternal chromosomes. A dosage proxy was calculated as the product of density and CDR length for each chromosome and haplotype. Pearson correlation coefficients and mean squared error (MSE) were computed between paternal and maternal values for each of the three metrics (density, length, and density × length) using NumPy. Chromosomes were flagged as outliers if their paternal–maternal residual exceeded a Z score threshold of $|Z| > 3.0$, where Z-scores were computed from the mean and standard deviation of the residual distribution across all chromosomes. Analyses were performed separately for young, old, and iPSC-derived cells. N is the number of chromosomal haploid types.

Read-level density thresholding

To classify individual reads as CDR or non-CDR based on their modification density, an optimal density threshold was determined for each chromosome for CENP-A, H3K9me3 and mCpG independently. Non-CDR active αSat regions were used as the background reference. For each chromosome, read-level modification densities were computed across 5,000 bp segments within CDR and non-CDR regions, with up to 15 segments sampled per chromosome from non-CDR regions to balance class representation. The optimal threshold was identified by exhaustive search over all midpoints between sorted density values, selecting the threshold that

minimized the weighted Gini impurity across CDR and non-CDR classes. Thresholds were determined separately for 6mA (filtering value ≥230) and 5mCG (filtering value ≥230) modification calls. Thresholds derived from this procedure were subsequently applied to classify reads in downstream single chromatin fiber analysis.

Density normalization

For each histone mark (CENP-A, H3K9me3, mCpG), mean signal density was computed per chromosome for each genomic region (center CDR, flanking CDR, and Non-CDR). Per-chromosome $\log_2$ fold-changes were calculated by dividing the regional mean density by a chromosome-specific baseline density derived from Non-CDR regions, followed by a $\log_2$ transformation. Separate baseline values were computed for early-passage and late-passage cells to control for global differences in signal levels. Values with a fold-change of zero or below were excluded prior to $\log_2$ transformation.

Epigenetic comparisons between early- and late-passage cells, or LCL and iPSCs

Differences in $\log_2$ fold-change between early- and late-passage cells were assessed using a paired two-tailed Student's *t* test, with chromosomes serving as paired units. Only chromosomes present in both early and late datasets were included in statistical comparisons. The number of shared chromosomes per comparison is reported in the results. A significance threshold of $p < 0.05$ was applied. n represents the number of chromosomes in the HG002 cell line.

hiPSC CDR prediction

Centromere Dip Regions in HG002 hiPSC haplotypes were identified using a sliding-window CDR caller applied to phased, per-CpG methylation bedgraph files derived from Oxford Nanopore long-read sequencing. For each haplotype array, a detection threshold was set at 10 percentage points below the array-wide mean methylation, accounting for the substantial variation in baseline methylation across centromeric arrays. Methylation signal was smoothed using a 10 kb sliding window stepped at 1 kb intervals (minimum 3 CpGs per window), and contiguous runs of below-threshold windows were called as candidate domains. Each candidate domain was assigned a composite reliability score (0–100) weighted by depth of hypomethylation (50%), CpG count (30%), and domain span (20%). Domains scoring ≥40 were retained for analysis; those scoring ≥50 were classified as HIGH_CONFIDENCE. CDR total span was defined as the interval from the start of the first to the end of the last HIGH_CONFIDENCE domain per haplotype. CDR calling was performed using cdr_caller_v2.py (available as Supplemental Code), implemented in Python 3 using NumPy and Matplotlib.

Cell Genomics, Volume *6*

Supplemental information

Haplotype-resolved DiMeLo-seq maps centromeric chromatin in a complete diploid human genome

Yuan Xu, Hailey Loucks, Julian Menendez, Fedor Ryabov, Julian K. Lucas, Monika Cechova, Luke Morina, Emily Xu, Danilo Dubocanin, Cy Chittenden, Mobin Asri, Ivo Violich, Christian Ortiz, Joshua M.V. Gardner, Todd Hillaker, Sara O'Rourke, Brandy McNulty, Tamara A. Potapova, Matthew W. Mitchell, Jacob P. Schwartz, Aaron F. Straight, Jennifer L. Gerton, Winston Timp, Ivan A. Alexandrov, Nicolas Altemose, and Karen H. Miga

Supplemental Information accompanying the paper

"Haplotype-resolved centromeric chromatin organization from a complete diploid human genome"

Figure S1: Diploid centromeric satellite annotations for HG002, related to Figure 1 2
Figure S2: Chromosome 9 HSat3 variation, related to Figure 1 3
Figure S3: Chromosome 1 HSat SF variation, related to Figure 1 4
Figure S4: Chromosome 3 and 4 Alpha and HSat1A variation, related to Figure 1 5
Figure S5: Moddotplot with paired 1D moddotplot track, related to Figure 1 6
Figure S6: HOR Structural Variant (StV) profiles, related to Figure 1 7
Figure S7: Chromosome 13 Structural Variant (StV) and HORhap analysis, related to Figure 1 8
Figure S8: CDR p-arm proximal regions, related to Figure 1 9
Figure S9: CDR association with homogenized regions, related to Figure 1 10
Figure S10: Total CDR span per haplotype in early passage LCL (HG002), related to Figure 1 11
Figure S11: CDR (subCDR and span) vs active array size, related to Figure 1 12
Figure S12: Coverage estimates of adaptive sampling, related to Figure 2 13
Figure S13: Clustering methylation data into groups, related to Figure 2 14
Figure S14: Fiber-seq nucleosome organization relative to CENP-B motifs, related to Figure 2 15
Figure S15: CENP-C DiMeLo-seq 6mA density (early passage), related to Figure 2 16
Figure S16: Methylation frequency across active arrays in early vs late passage, related to Figure 3 17
Figure S17: Difference in sub-CDR structure in early vs late passage, related to Figure 3 18
Figure S18: Enrichment within sub-CDRs across early- and late-passage, related to Figure 3 19
Figure S19: Comparison between HG002 early passage and hiPSC CDRs, related to Figure 3 20
Figure S20: CENP-A DiMeLo-seq Density in hiPSCs, related to Figure 3 21
Figure S21: CENP-A DiMeLo-seq density in late passage lines, related to Figure 3 22
Figure S22: hiPSC array CpG density and CDR prediction, related to Figure 3 23

References 24

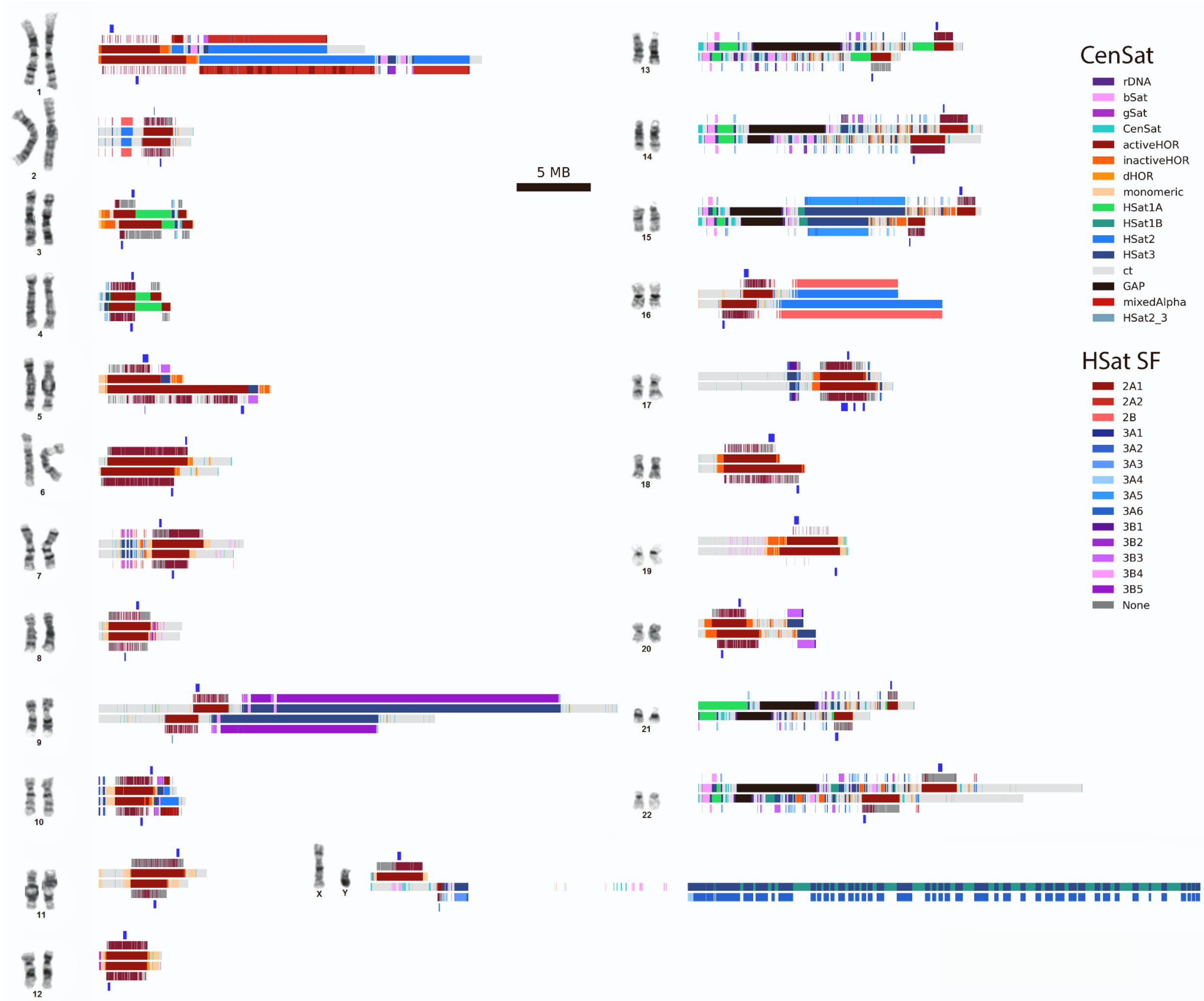

Figure S1: Diploid centromeric satellite annotations for HG002, related to Figure 1

HG002 g-banding karyotype images displaying centromeric satellite (censat) annotation tracks for all chromosomes in the diploid HG002 genome, generated using an automated annotation method. For each chromosome, the maternal haplotype is shown on top and the paternal haplotype on the bottom. Annotation tracks are arranged symmetrically from the center outward and include: (1) cenSat family annotations, (2) alpha-satellite higher-order repeat (HOR) haplotypes and satellite superfamilies (HSat SF), and (3) centromeric dip regions (highlighted in blue). Alpha-satellite HOR haplotypes are colored on a gradient from younger HORs (red) to older HORs (grey)

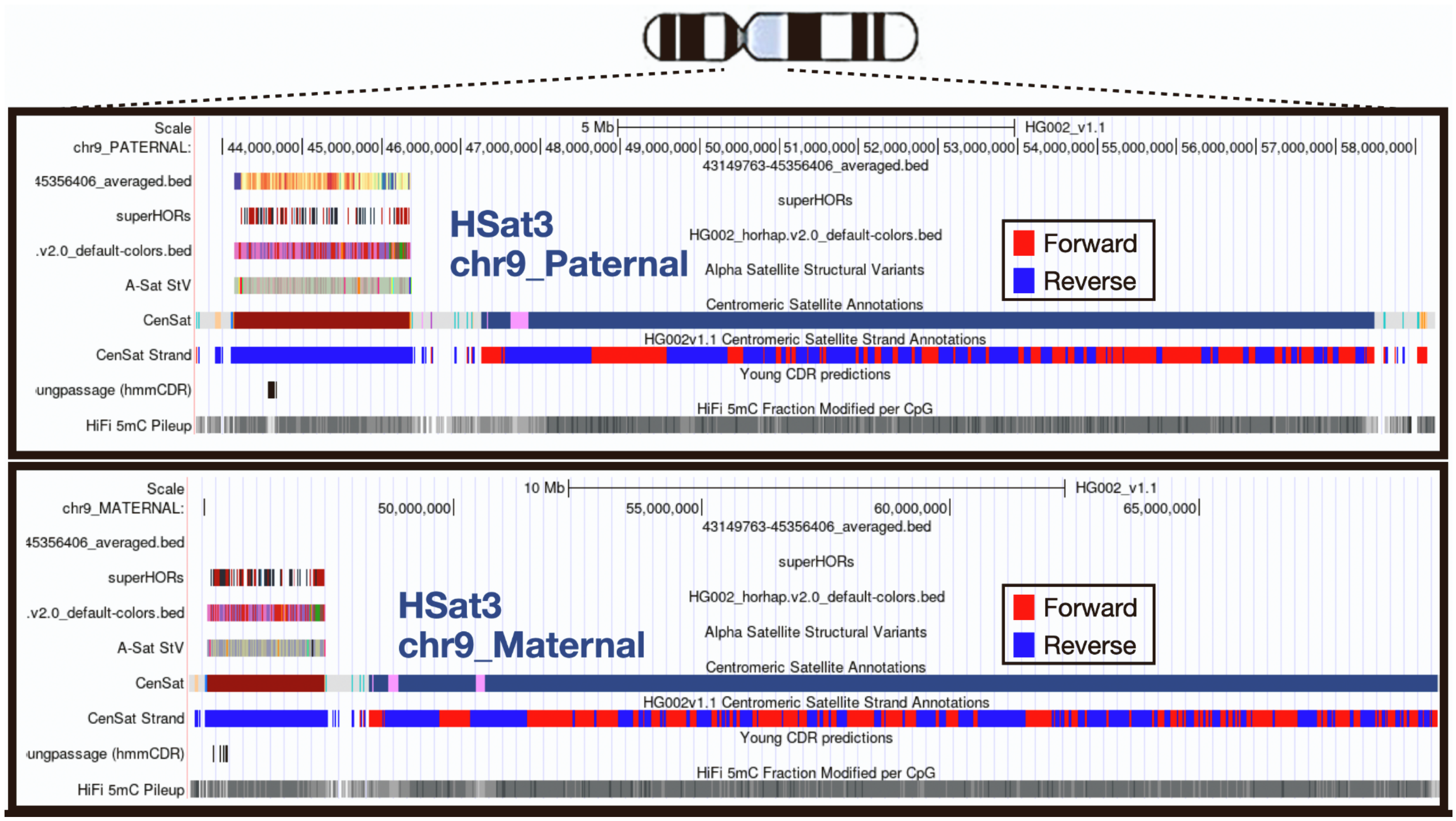

Figure S2: Chromosome 9 HSat3 variation, related to Figure 1

UCSC Genome Browser track hub [1] visualizations of the centromeric region of chromosome 9 for the maternal (chr9_maternal: 45–70 Mb) and paternal (chr9_paternal: 44–58 Mb) haplotypes, displaying cenSat satellite annotations. Unlike most satellite families, which are arranged head-to-tail in a single orientation (e.g., alpha satellite, dark red, cenSat track), Human Satellite 3 (HSat3, dark blue) on chromosome 9 exhibits both length variation and major shifts in repeat orientation across the array. Forward and reverse HSat3 orientations are shown in red and blue, respectively, revealing multiple megabase-scale strand domains arranged in large blocks. The order and polarity of these orientation segments differ between maternal and paternal chromosomes, consistent with large internal inversions and strand-polarized repeat substructure across the HSat3 array.

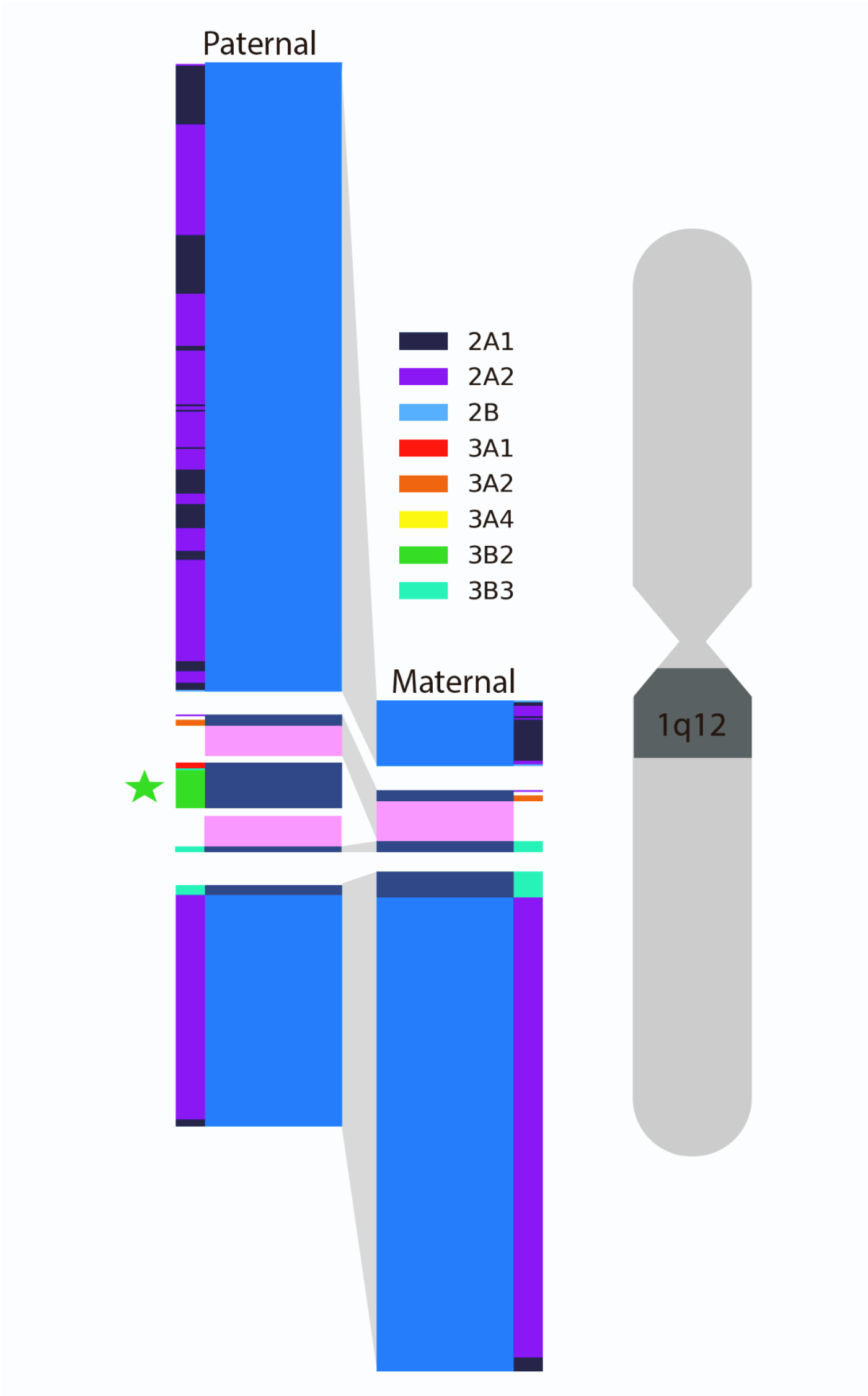

Figure S3: Chromosome 1 HSat SF variation, related to Figure 1

Comparison of Chromosome 1q12 region between paternal and maternal haplotypes, large Hsat2 arrays have been truncated and are not to scale. Outer columns represent HSat superfamilies, and thicker inner columns represent CenSat annotation. Homologous arrays are connected. HSat2 on chromosome 1 differs by 6.74 Mb between homologs and contains distinct sequence compositions. The paternal haplotype contains a unique HSat3B2 array marked with a star and an adjacent beta-satellite sequence, while the paternal haplotype contains an additional ~500 kb polymorphic block of HSat3 with an adjacent beta-satellite sequence.

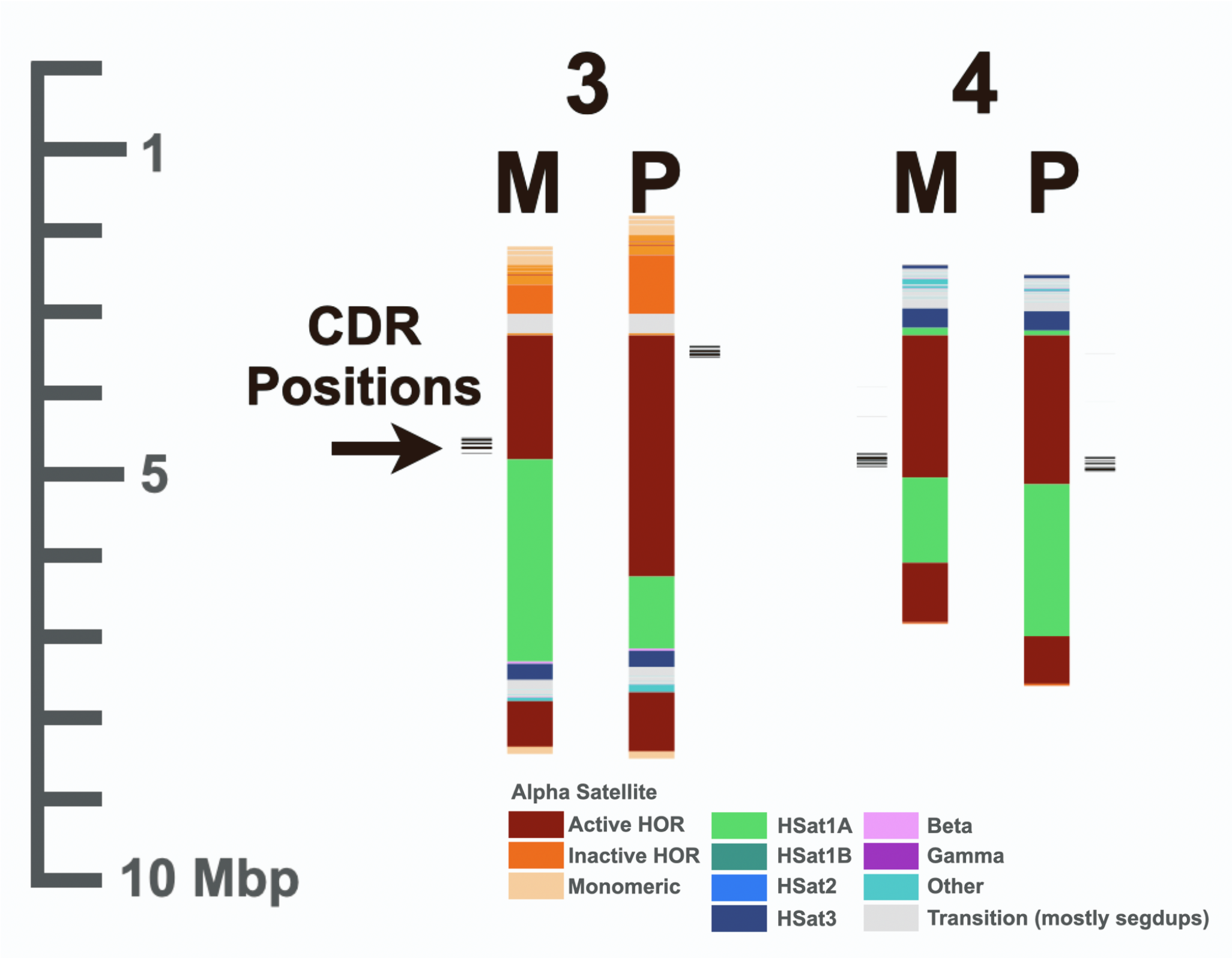

Figure S4: Chromosome 3 and 4 Alpha and HSat1A variation, related to Figure 1

CenSat annotations are shown for the maternal (M) and paternal (P) haplotypes of chromosomes 3 and 4, with satellite subfamily identity indicated by color (see key). Scale bars are provided for each haplotype. Centromere dip regions (CDRs), which mark the position of the kinetochore-associated active alpha-satellite (αSat) array, are indicated by black tick marks; the CDR on chromosome 3M is highlighted by an arrow. These chromosomes illustrate two distinct forms of centromeric structural variation. First, interspersed HSat1A arrays (green) are embedded within the active αSat arrays on both chromosomes, splitting the array into discontinuous blocks and differing in size between haplotypes (~0.61 Mb on chr3; ~1.1 Mb on chr4). Second, the active αSat arrays differ in total length between haplotypes, reflecting haplotype-specific restructuring of kinetochore-associated centromeric domains. On chromosome 3, additional complexity is observed in the form of interspersed centromere transition (CT) regions containing lncRNA candidate loci (grey), as well as HSat3 and other smaller centromeric satellite classes, indicating a more structurally heterogeneous array organization relative to chromosome 4.

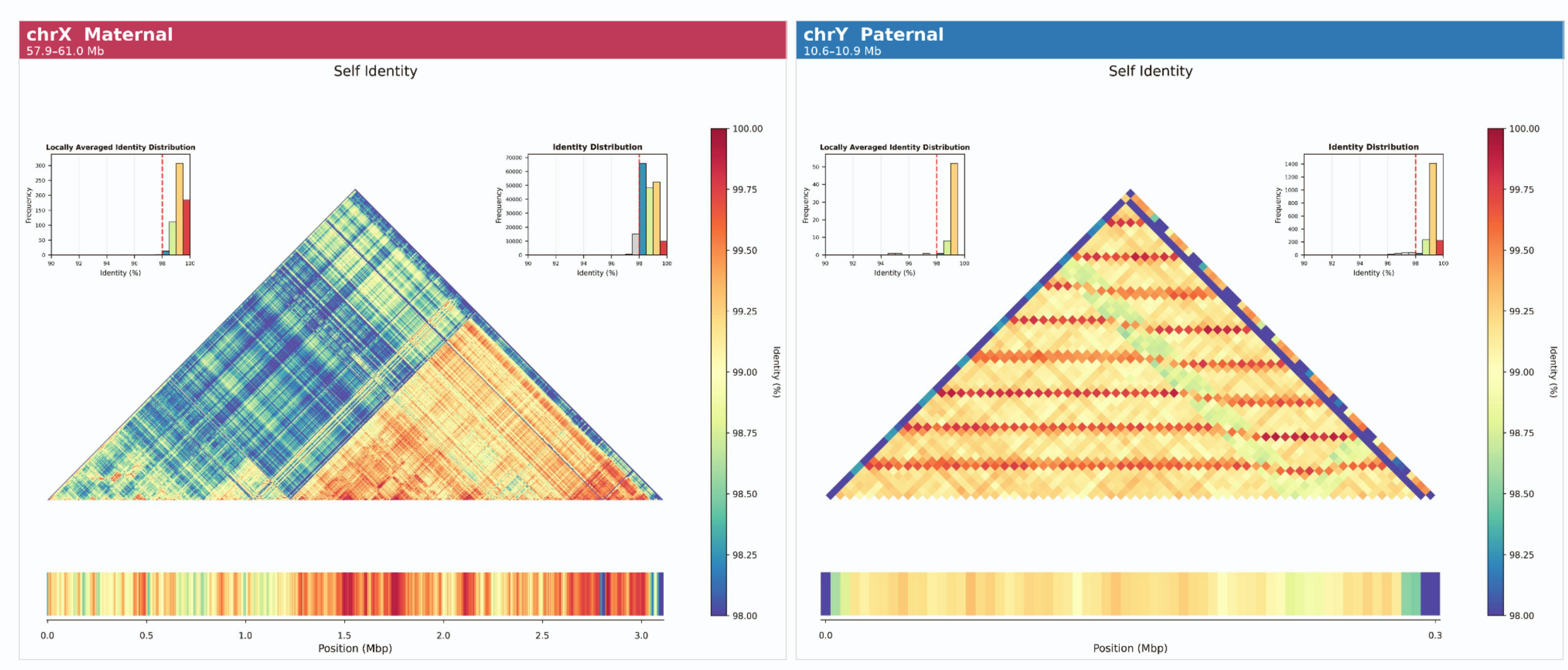

Figure S5: Moddotplot with paired 1D moddotplot track, related to Figure 1

Triangular self-identity heatmaps are shown for the active alpha-satellite HOR (αSat) arrays of X and Y chromosomes. Active arrays were identified from the HG002v1.1 assembly using the cenSat v2.0 annotation by selecting entries with the suffix "H1L." Adjacent intervals within 200 kb were merged, and FASTA sequences for each array were extracted and input to ModDotPlot[2] (window size = 5 kb, $\delta = 0$) to generate all-vs-all pairwise identity values. Identity values were locally averaged across the two nearest neighboring windows on each side using a custom Python script. The resulting values are displayed on a color scale ranging from a fixed lower bound of 90% identity (or the 10th percentile of observed values if higher) to 100%, with warmer colors (red/orange) indicating higher identity and cooler colors (blue/purple) indicating lower identity. The bar below each triangle represents the locally averaged identity at each position along the array. Inset histograms show the distributions of locally averaged identity (left) and raw all-vs-all pairwise identity (right); the red dashed line indicates the mean. Genomic coordinates (GRCh38) are indicated in the panel header.

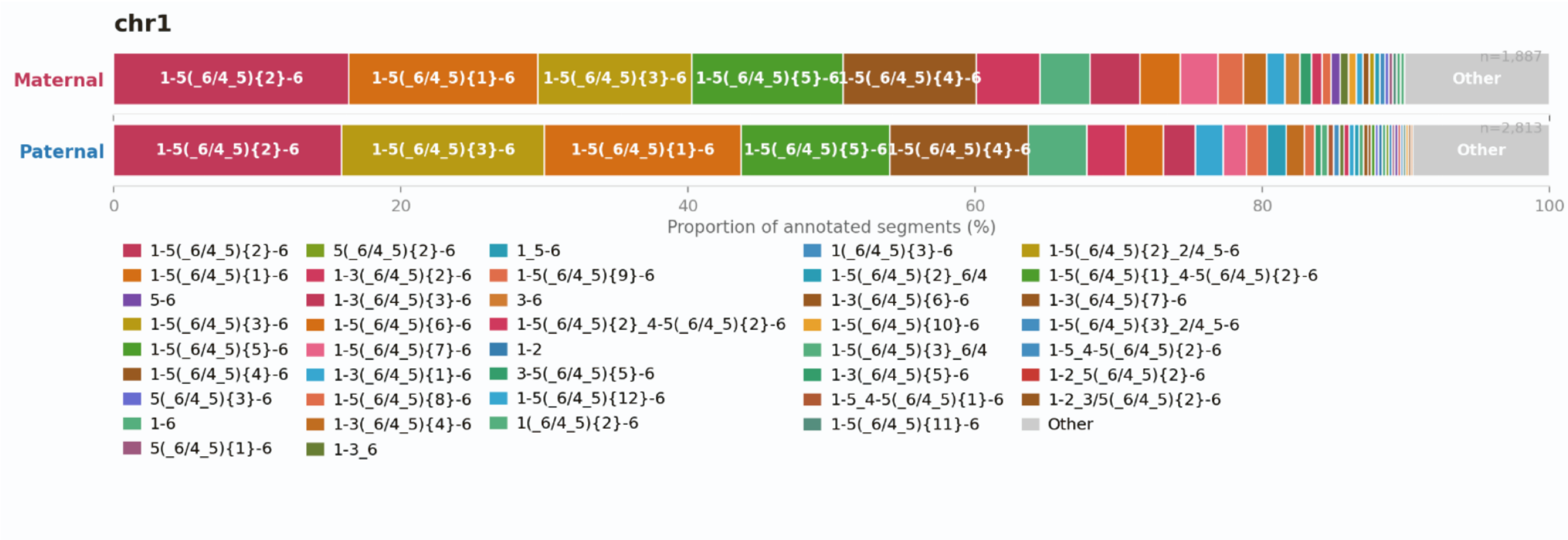

Figure S6: HOR Structural Variant (StV) profiles, related to Figure 1
Stacked horizontal bar charts showing the proportional distribution of alpha-satellite HOR structural variants for chromosome 1 (as an example, with all chromosomes referenced in Table S4), with maternal (top) and paternal (bottom) haplotypes shown separately. Each colored segment represents a unique HOR variant, labeled by its structural suffix (common chromosome-specific HOR prefix removed for clarity). Segment width reflects the proportion of total annotated array segments occupied by each variant. Variants with fewer than 5 are grouped into "Other" (grey). The total number of annotated segments per haplotype (n) is indicated at the right of each bar.

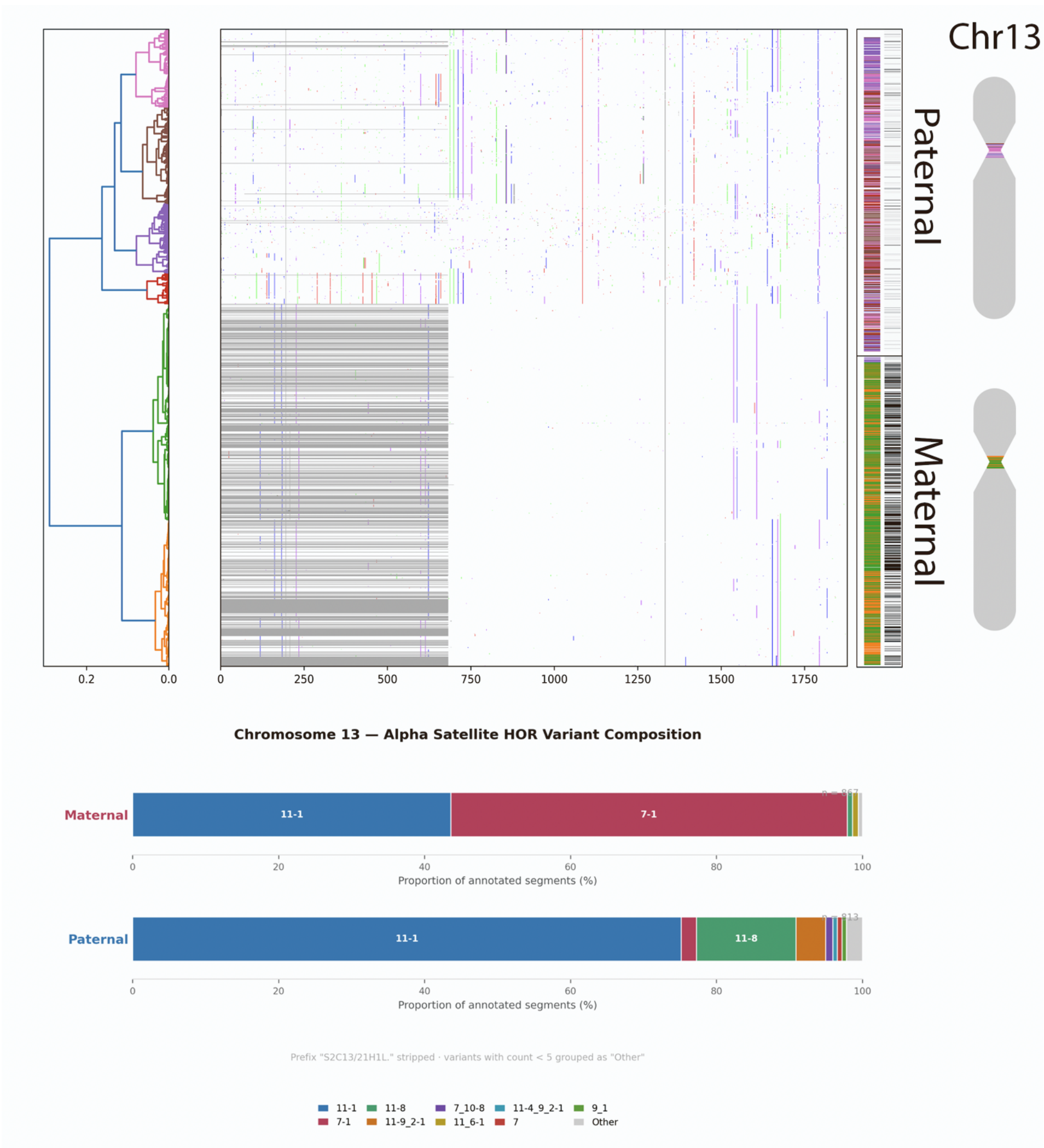

Figure S7: Chromosome 13 Structural Variant (StV) and HORhap analysis, related to Figure 1
Ward HORhap clustering for chromosome 13 in HG002. Sequences of the two most frequent HOR structural variants (StVs) (the full-length S2C13/21H1L.11-1 and S2C13/21H1L.7-1 with a deletion of four monomers) from maternal and paternal arrays were aligned together. Only positions that differ from the consensus are colored (center). HORs in the alignment are ordered according to the Ward dendrogram (left), which is split into six colored clades (HORhaps). On the right, paternal and maternal centromeres (shown one above the other) are displayed as HORhap tracks with the corresponding colors (left) and tracks showing the positions of 7-mer StVs (right). On the far right, the positions of the centromeres within the entire chromosomes are indicated. The specificity of HORhaps to homologs is evident: the paternal homolog consists only of pink, brown, purple, and red HORhaps, corresponding to the upper major branch of the dendrogram, whereas the maternal homolog consists almost entirely of green and orange HORhaps, corresponding to the lower major branch, with only the very tip of the p-arm (upper end) represented by HORhaps from the upper major branch, and deletions are visible in grey.

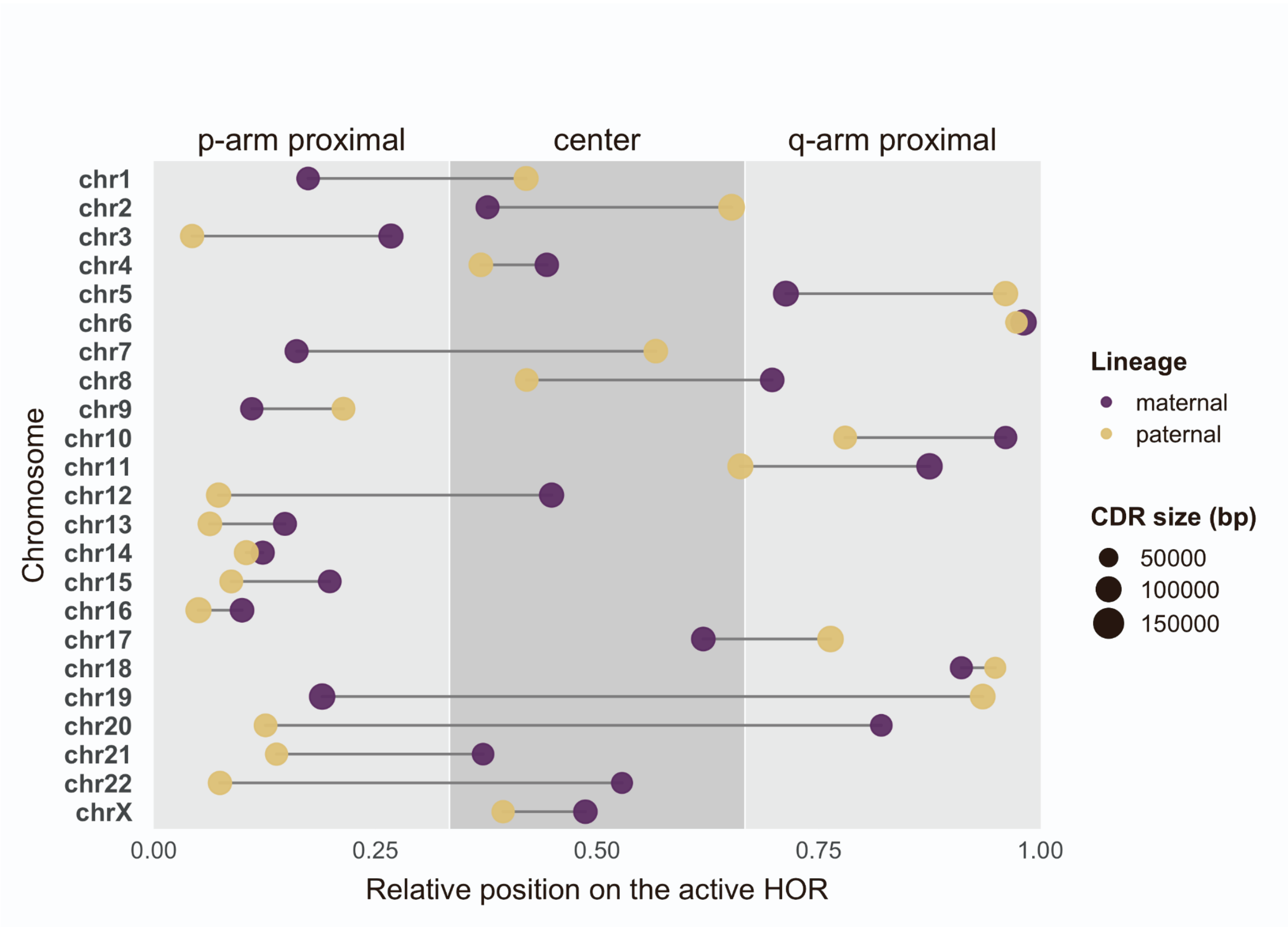

Figure S8: CDR p-arm proximal regions, related to Figure 1

The relative position and size of CDRs within the active higher-order repeat array for each chromosome. The relative location of the CDR midpoint within the active array (x-axis; split equally between p-arm proximal, center, and q-arm proximal location) is noted for each chromosome (y-axis). The size of the dot is relative to the size of the CDR, here defined as the sum of all the subCDRs. Finally, the maternal and paternal CDRs for each homologous chromosome (including X and Y) are connected by a horizontal line. The p-arm proximal location is the most frequent out of the three possibilities.

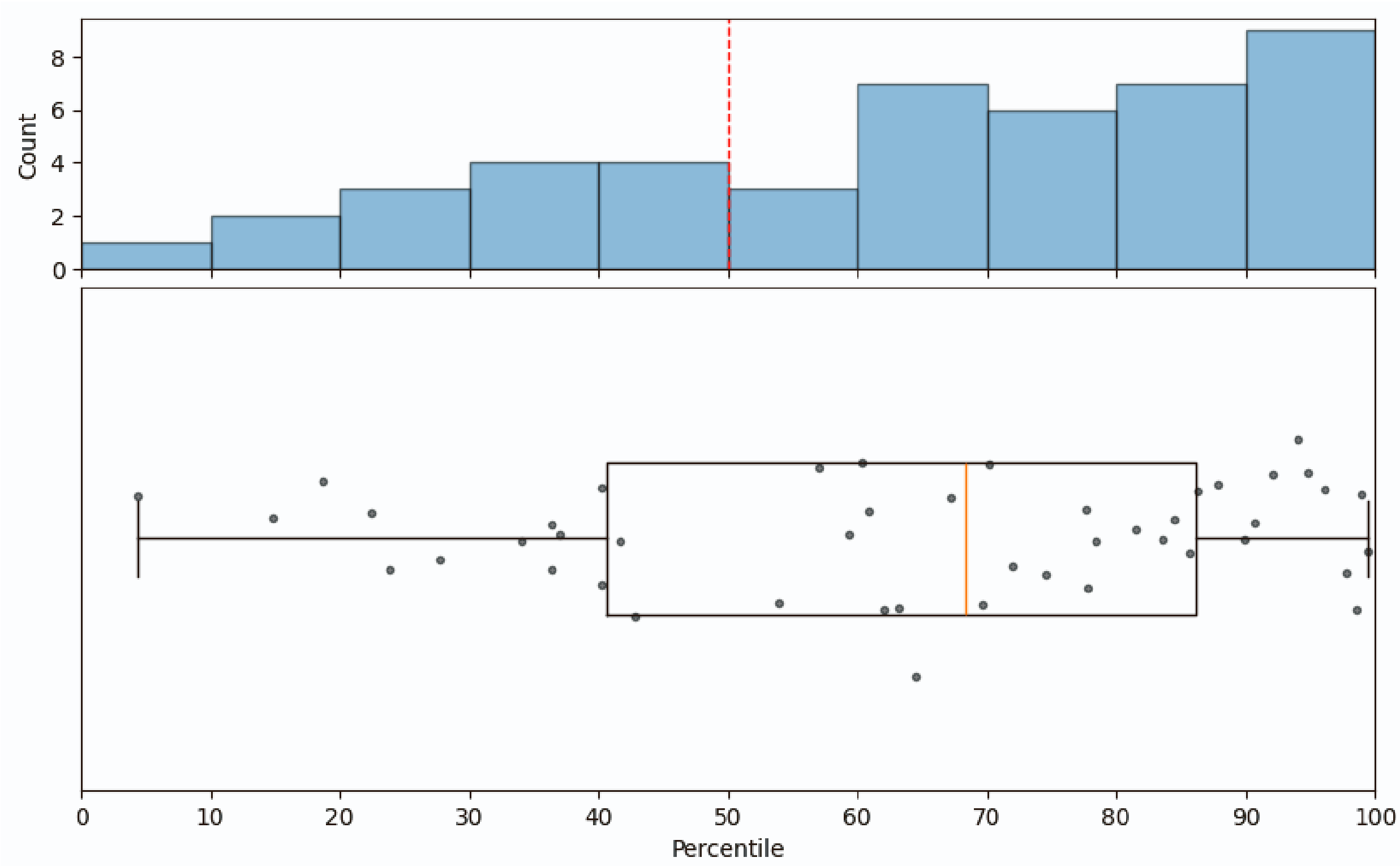

Figure S9: CDR association with homogenized regions, related to Figure 1

CDR local identity as a percentile of the active alpha array distribution. For each chromosomal CDR, defined as the span between the two outermost subCDRs on a given haplotype, mean local identity was calculated across overlapping bins. To assess relative self-similarity, each CDR's mean local identity was compared to the background distribution of local identities across the whole active array. Each chromosome was assigned a percentile score reflecting where its CDR mean local identity falls within the active array distribution. The bottom panel shows per-chromosome CDR percentiles as a scatter-boxplot, with the top panel displaying the corresponding distribution as a histogram.

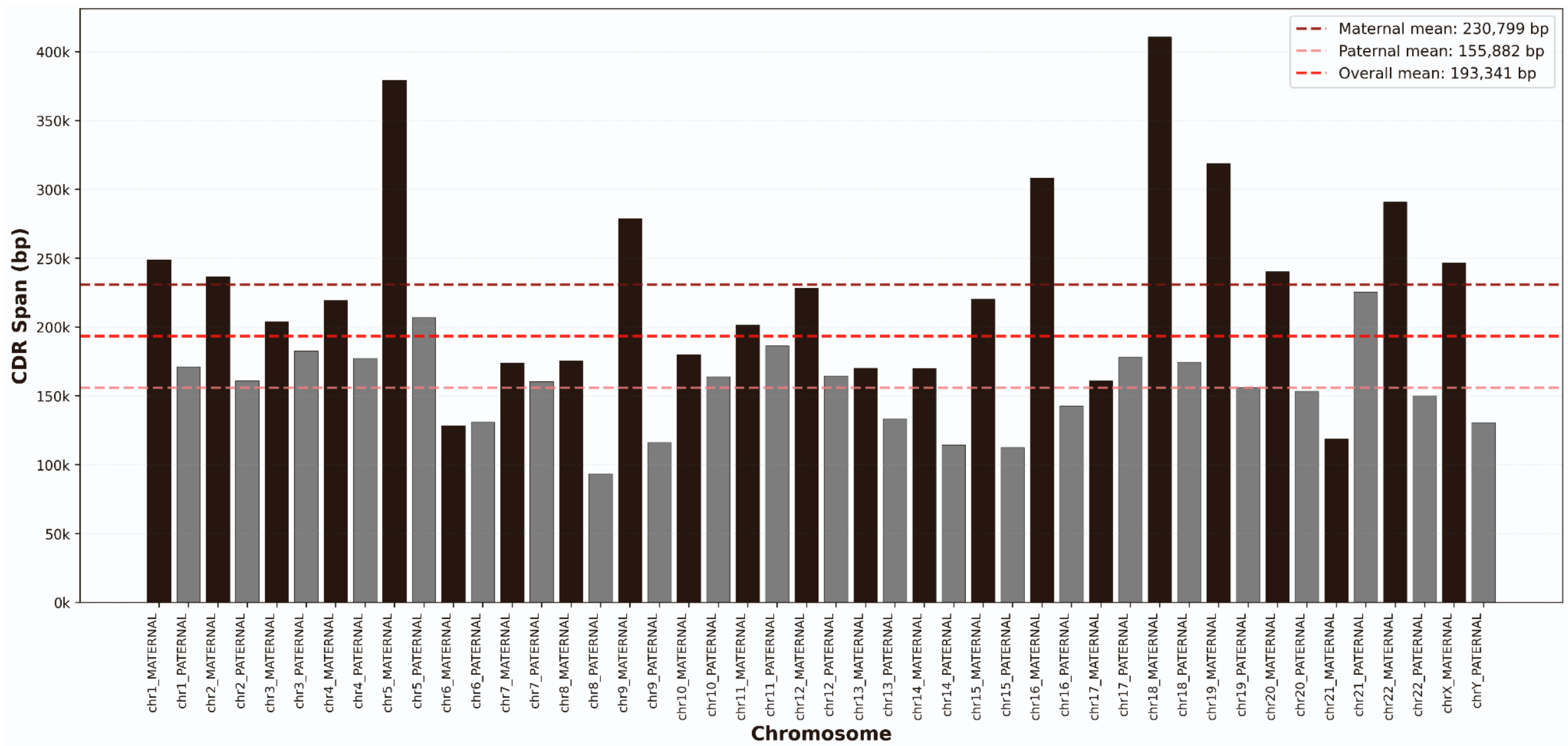

Figure S10: Total CDR span per haplotype in early passage LCL (HG002), related to Figure 1
Stacked bar plots showing the aggregate CDR span (kb) per haplotype across all 46 phased assemblies (chr1–chrY), as called by hmmCDR from DiMeLo-seq CENP-A data in the young passage LCL. Each bar represents a single haplotype (M, maternal; P, paternal), with individual stacked segments corresponding to discrete CDR subdomains; segment shading cycles from light to dark grey to distinguish adjacent subdomains. Horizontal reference lines indicate the mean aggregate CDR span across all maternal haplotypes (dark red dotted), all paternal haplotypes (light red dotted), and all haplotypes combined (red dashed). Mean aggregate CDR span was 87.4 kb (maternal), 89.4 kb (paternal), and 88.4 kb genome-wide across 254 total CDR domains.

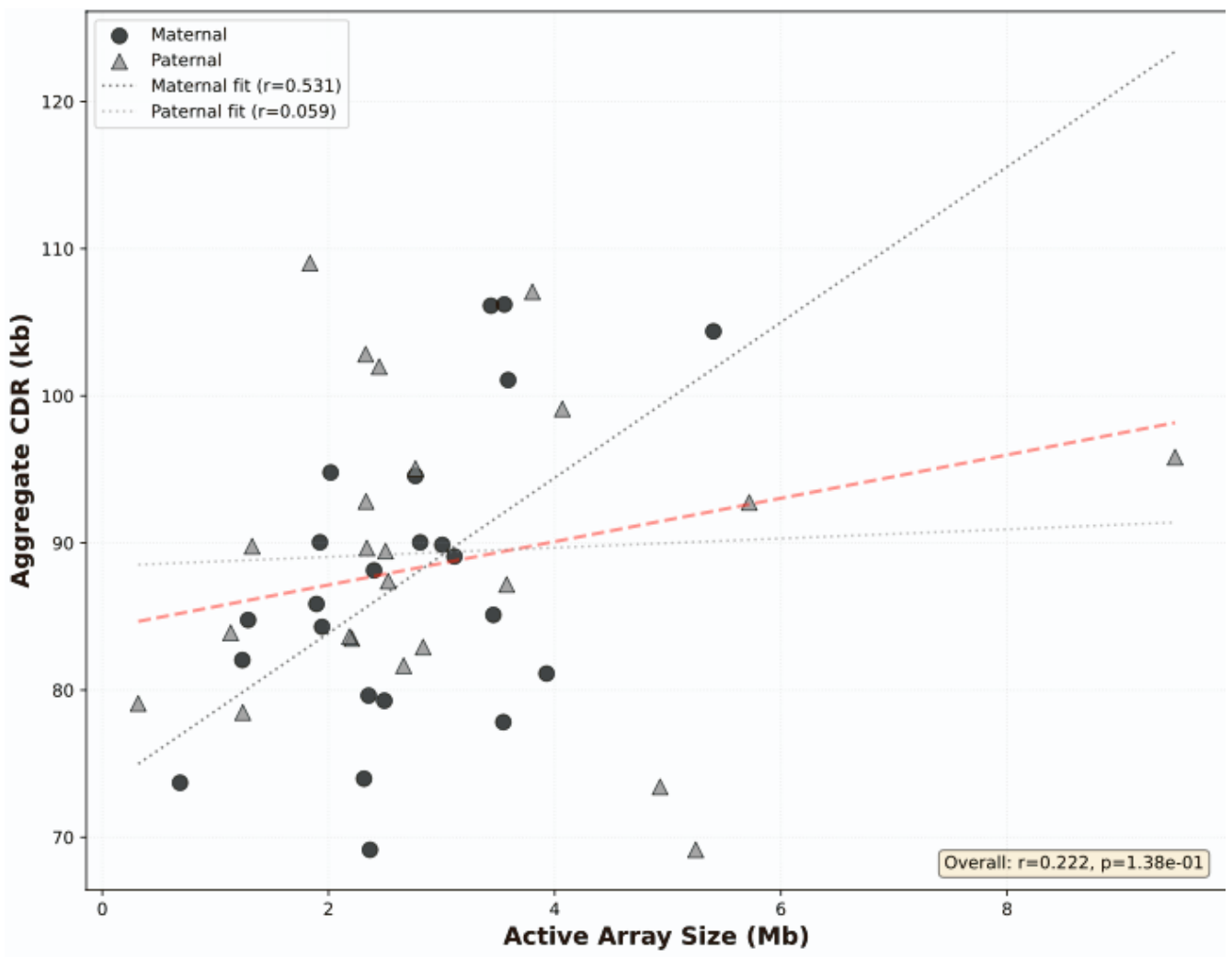

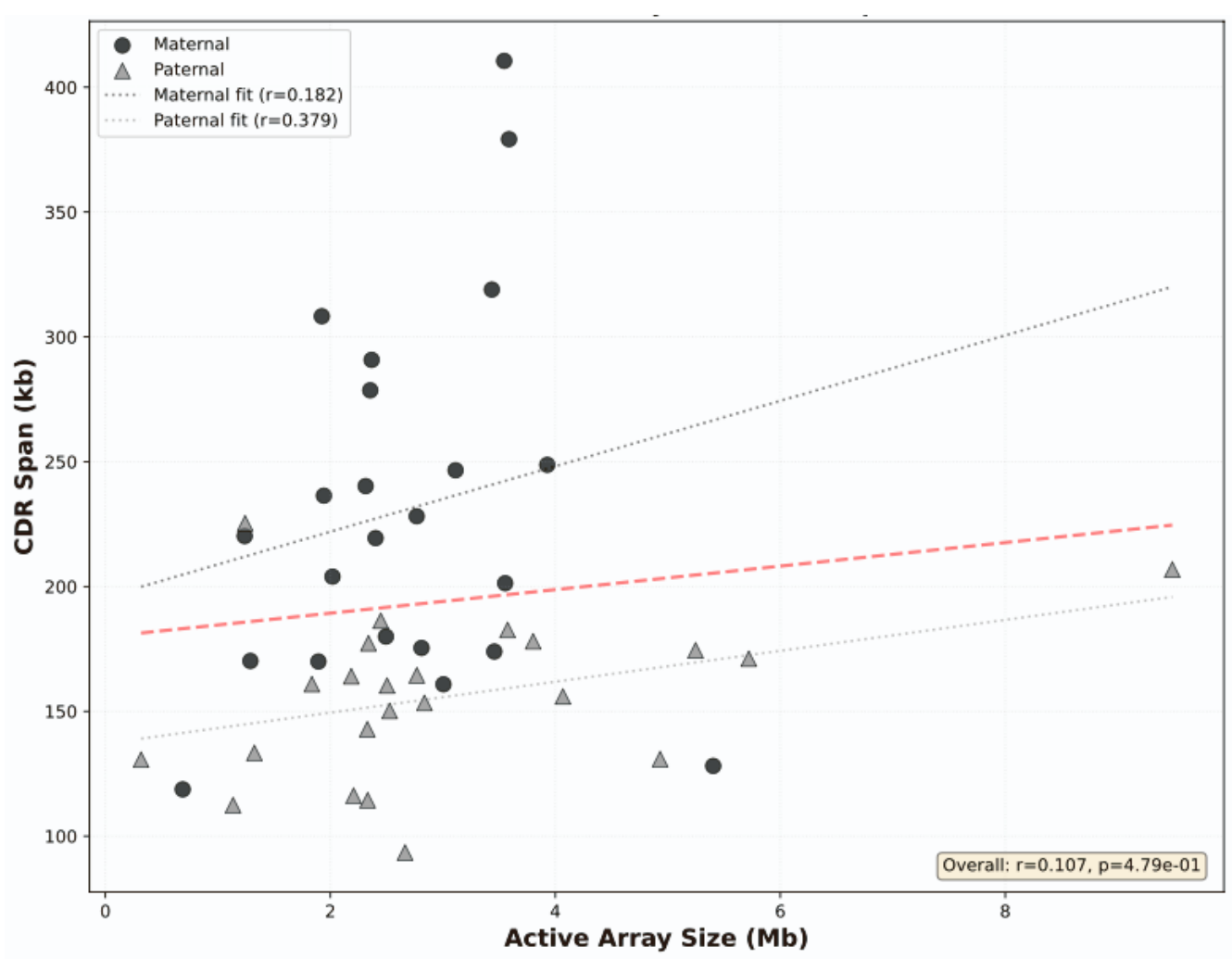

Figure S11: CDR (subCDR and span) vs active array size, related to Figure 1

Scatter plots showing the relationship between CDR length (x-axis) and total active αSat HOR array length (y-axis, Mb) for all chromosomes. Maternal haplotypes are shown as dark grey circles and paternal haplotypes as light grey squares; dashed lines indicate linear fits with associated Pearson r-values. (Top) Aggregated sub-CDR length, calculated as the sum of individual hypomethylated sub-CDR segments, excluding intervening regions between domains. The aggregated sub-CDR length is highly consistent genome-wide (mean ~90 kb; range: 69–109 kb) and shows no significant correlation with array size (maternal r = 0.059; paternal r = 0.059). (Bottom) Total CDR span, measured from the start of the first sub-CDR to the end of the last sub-CDR, including intervening non-hypomethylated sequence. Total span is more variable and shows a modest positive correlation with array size (maternal r = 0.182; paternal r = 0.379). Together, these plots demonstrate that while CDR span varies across haplotypes and chromosomes, the aggregate length of the hypomethylated sub-CDR core is remarkably stable and decoupled from the size of the underlying active HOR array.

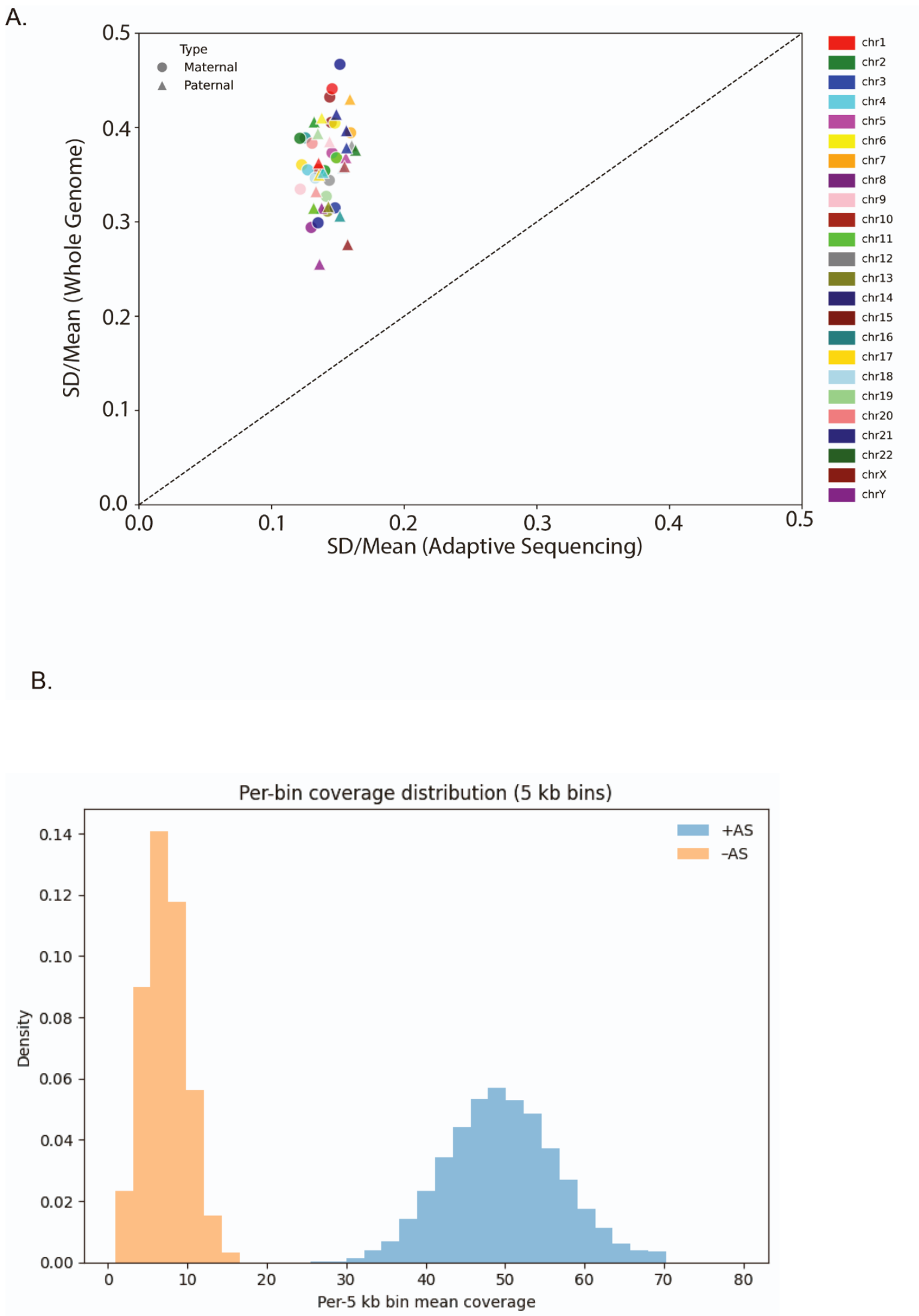

Figure S12: Coverage estimates of adaptive sampling, related to Figure 2

Adaptive sampling achieves uniform coverage across centromeric arrays. (A**)** Scatter plot comparing coverage uniformity between whole genome sequencing and adaptive sampling across centromeric regions. The coefficient of variation (SD/mean) for whole genome sequencing (y-axis) is broadly distributed, whereas the coefficient of variation for adaptive sampling (x-axis) is consistently low, demonstrating that adaptive sampling yields substantially more uniform coverage across centromeric arrays relative to whole genome sequencing. (B**)** Overlaid density histograms of mean sequencing coverage across 5 kb genomic bins for adaptive sampling (+AS) and whole genome sequencing (−AS). Coverage values were calculated by aggregating per-base depth into non-overlapping 5 kb windows. The +AS distribution is narrow and concentrated around the mean, reflecting highly uniform coverage across centromeric regions, whereas the −AS distribution is broadly dispersed, indicating greater bin-to-bin coverage variability in whole genome sequencing.

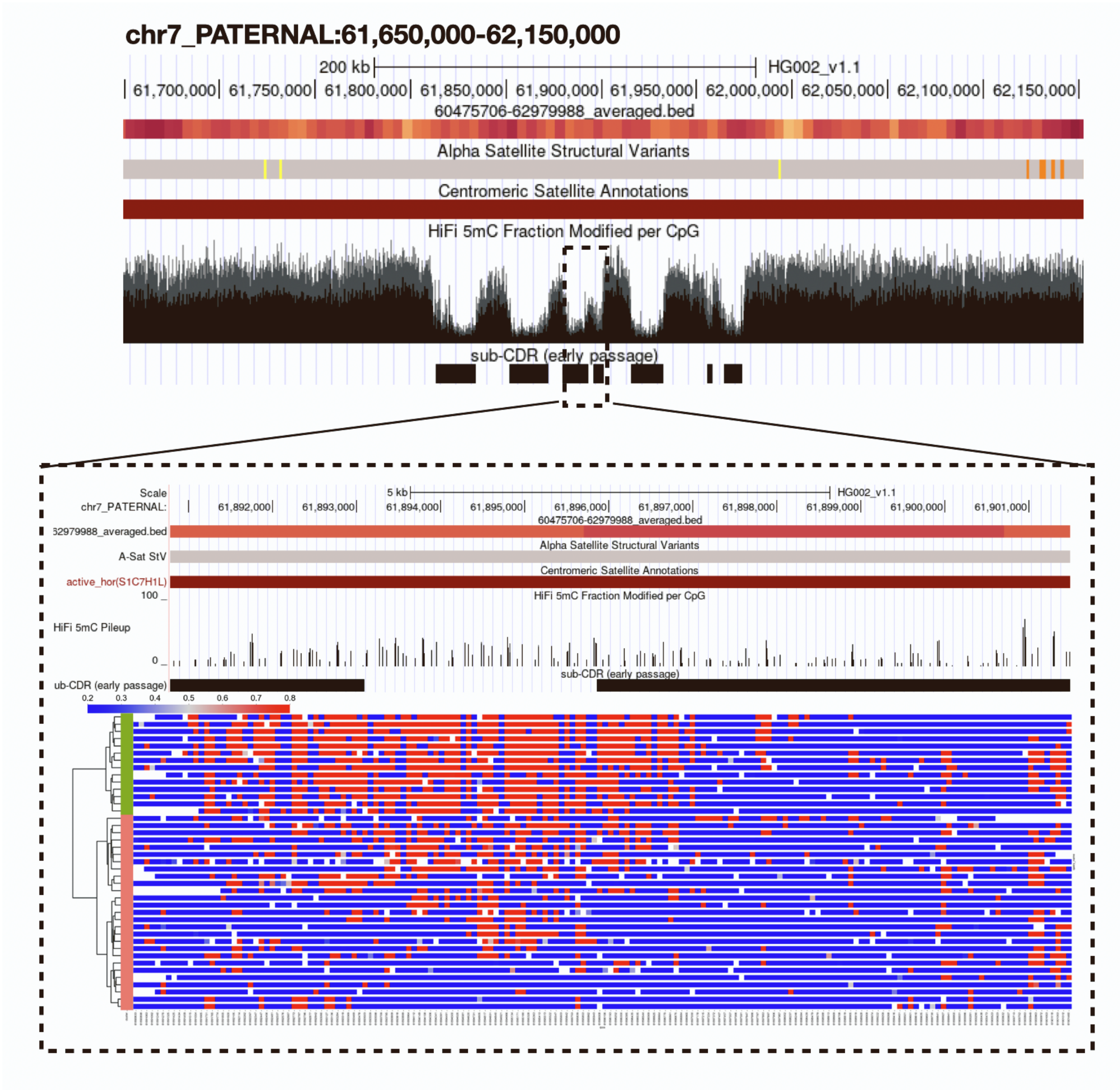

Figure S13: Clustering methylation data into groups, related to Figure 2

Clustered read matrices showing CpG methylation (5mC) signal across individual reads at the CDR edge in chromosome 7 paternal. Each row represents a single sequencing read, and each column a CpG position within the region. Reads were clustered using HDBSCAN based on their per-CpG 5mC probability profiles, with only reads covering at least 95% of the locus retained (windowFrac = 0.95). Distinct clusters reflect discrete molecule-level methylation states, supporting the interpretation that epigenetic heterogeneity at these regions is a genuine biological signal.

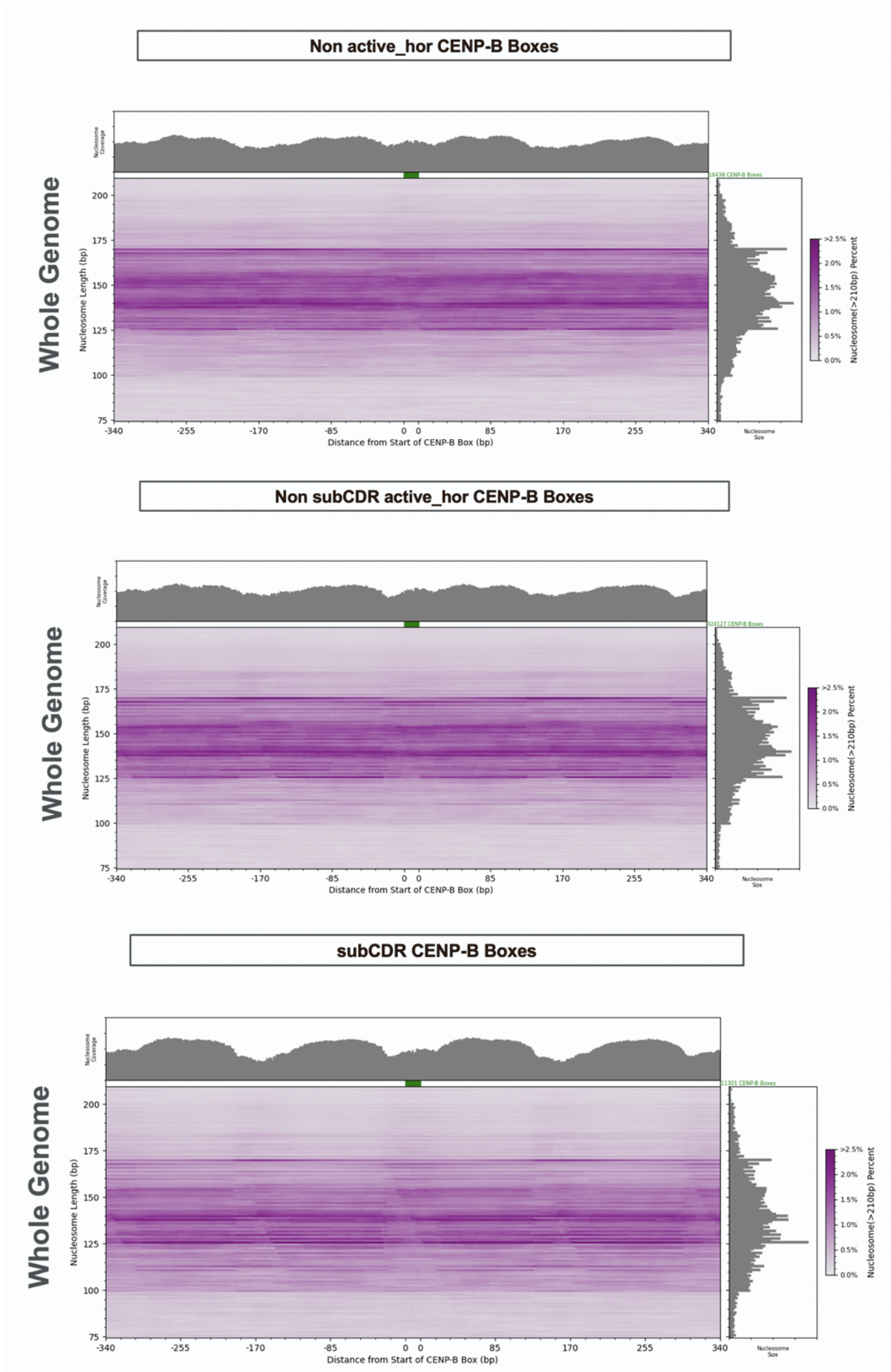

Figure S14: Fiber-seq nucleosome organization relative to CENP-B motifs, related to Figure 2
Fiber-seq[3] comparative nucleosome organization relative to centromere protein B (CENP-B) 17-bp motifs[4] in CDR, Active, and Inactive Alpha Arrays. Nucleosome positions with respect to reference coordinates were extracted using fibertools [5] extract. For each of the three regions (within subCDRs, non-CDR active alpha array, and inactive alpha array), a matrix was filled with the position of the nearest CENP-B motif and nucleosome-size. The matrix was then column-normalized to estimate the percentage of nucleosomes at specific sizes per distance from a CENP-B motif. Darker heatmap regions indicate a higher density of nucleosomes at a given size and position. Count totals are projected onto each axis as marginal histograms, summarizing the row and column distributions of the matrix. The top histogram summarizes nucleosome positional distribution relative to the CENP-B box motif, while the right histogram shows the overall nucleosome size distribution within each of the three regions observed.

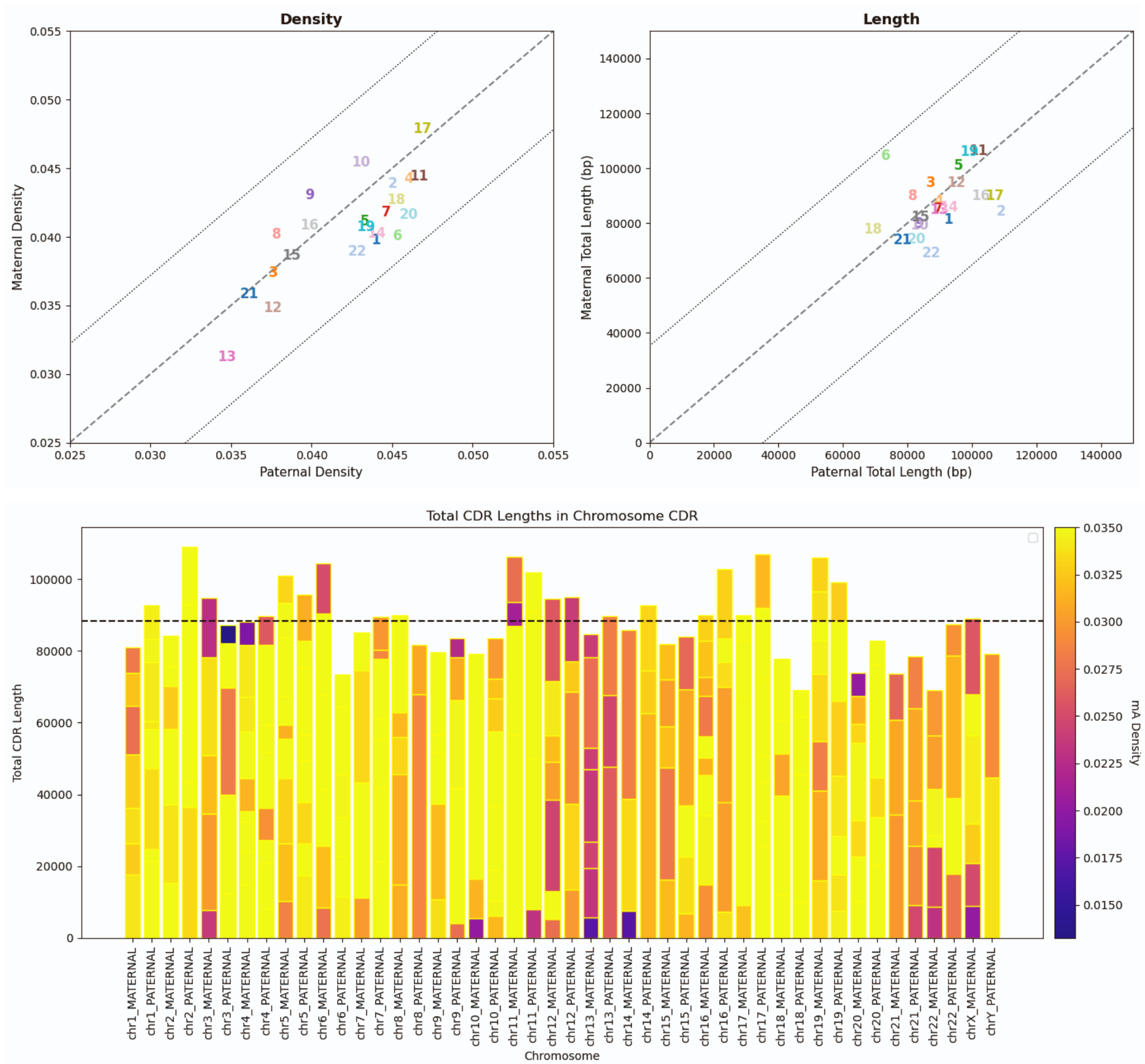

Figure S15: CENP-C DiMeLo-seq 6mA density (early passage), related to Figure 2
Centromere protein C (CENP-C) chromatin dosage is balanced between homologous centromeres in early-passage LCLs. (A) Scatter plots comparing paternal (x-axis) and maternal (y-axis) CENP-C DiMeLo-seq[6] 6mA signal density (left) and total sub-CDR length (right) across all chromosomes in early-passage HG002 LCLs. Each chromosome is labeled and color-coded. The dashed diagonal line represents the identity line; dotted lines indicate ±3 SD outlier thresholds. Chromosomes deviating beyond these thresholds represent haplotype-specific departures from dosage balance. (B) Stacked bar plots showing total CDR length per chromosome for maternal and paternal haplotypes for CENP-C DiMeLo-seq. Each bar is subdivided into individual sub-CDR segments colored by 6mA signal density using a plasma colormap. The dashed horizontal line indicates the mean total CDR length across all homologs. A color bar indicates the range of 6mA density values. Maternal and paternal haplotypes are displayed as adjacent pairs for each chromosome, ordered from chr1 to chrY.

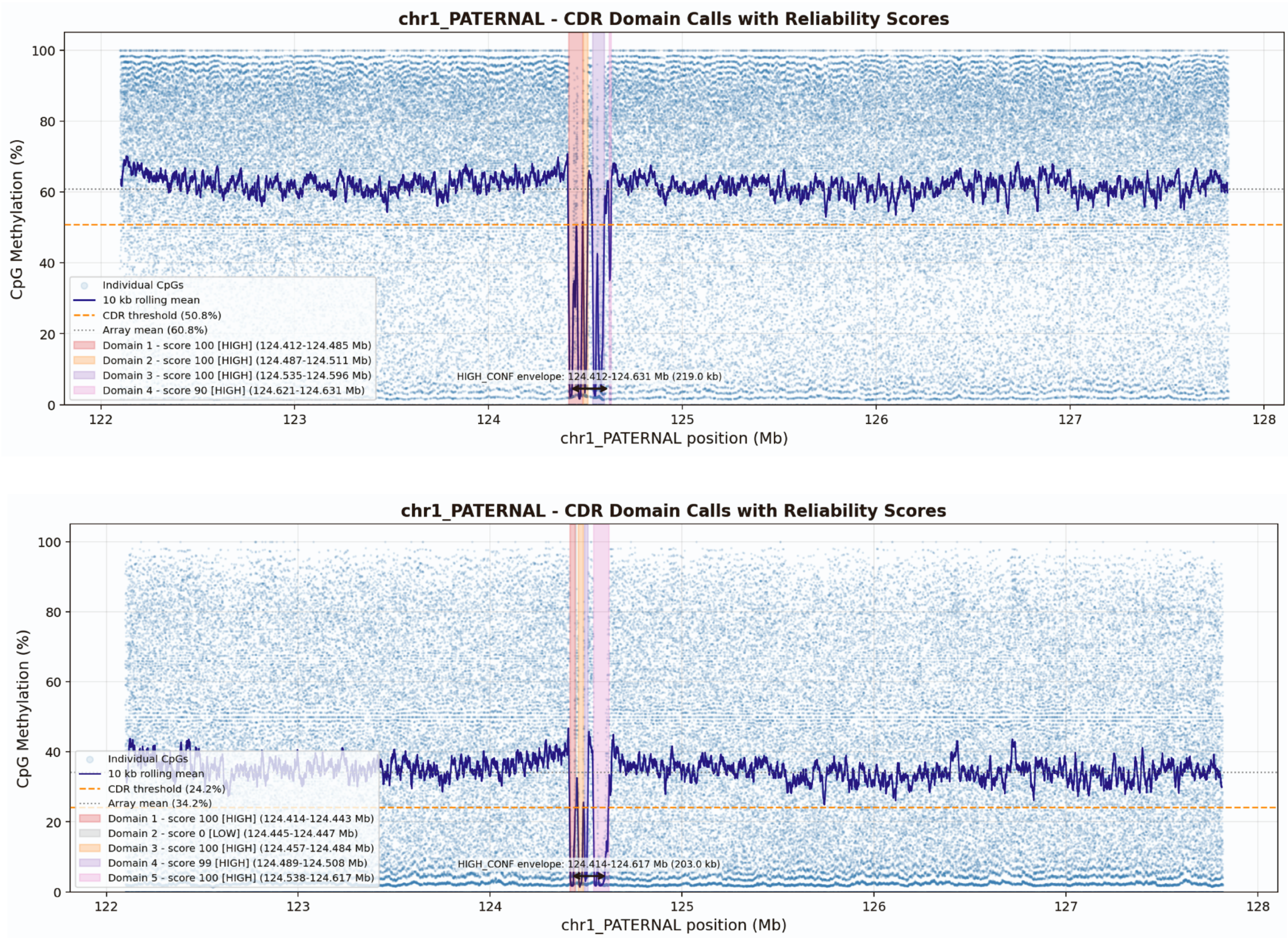

Figure S16: Methylation frequency across active arrays in early vs late passage, related to Figure 3

Methylation plot pileup (specifically BEDmethyl/bedGraph formats) summarizes per-base DNA methylation frequencies (e.g., 5mC) from CENP-A DiMeLo-seq data across the active array of chromosome 1_PATERNAL early passage (EP) (top) and late passage (LP) (bottom). Individual windows of CpG are provided in light blue, with a 10kb rolling mean in dark blue. The dotted line indicates the array mean (as shown for chromosome 1_PATERNAL, EP=60.8%, and LP=34.2%). CDRs are highlighted as individual domains that pass the threshold (10% difference from the array mean), and are shown to highlight that the CDRs remain in the same span, but have altered sub-CDR boundaries.

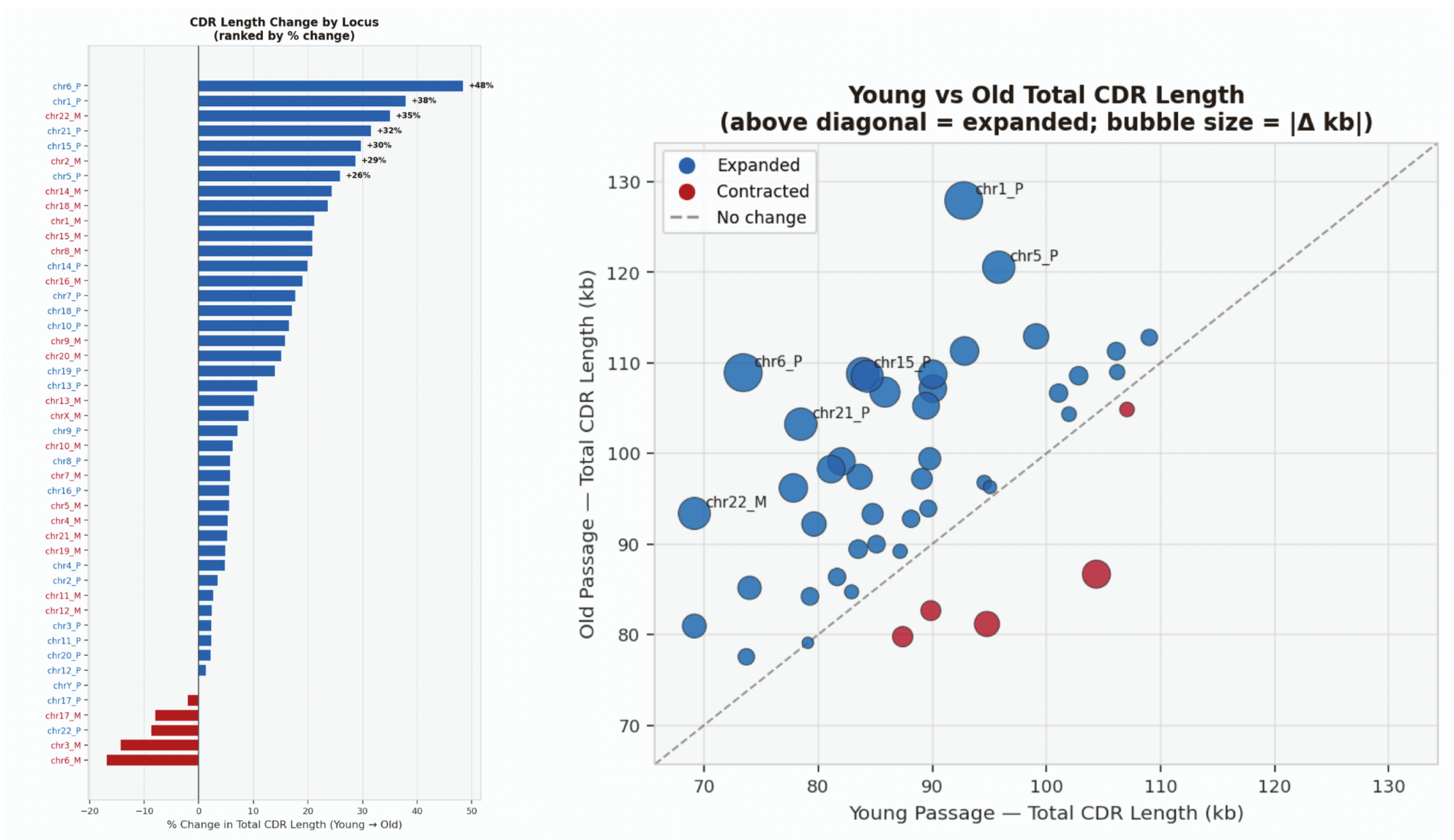

Figure S17: Difference in sub-CDR structure in early vs late passage, related to Figure 3

(Left) Ranked percent change in total sub-CDR aggregate length between early and late passage, shown for all active αSat arrays. Arrays are rank-ordered by magnitude of change, with increases shown in blue and decreases in red. Maternal and paternal haplotypes are labeled in red and blue, respectively. (Right) Scatter plot comparing total sub-CDR aggregate length (kb) between early (x-axis) and late (y-axis) passage. Each point represents a single array, with circle size proportional to the absolute magnitude of change between passages. The dashed line indicates the expectation under no change.

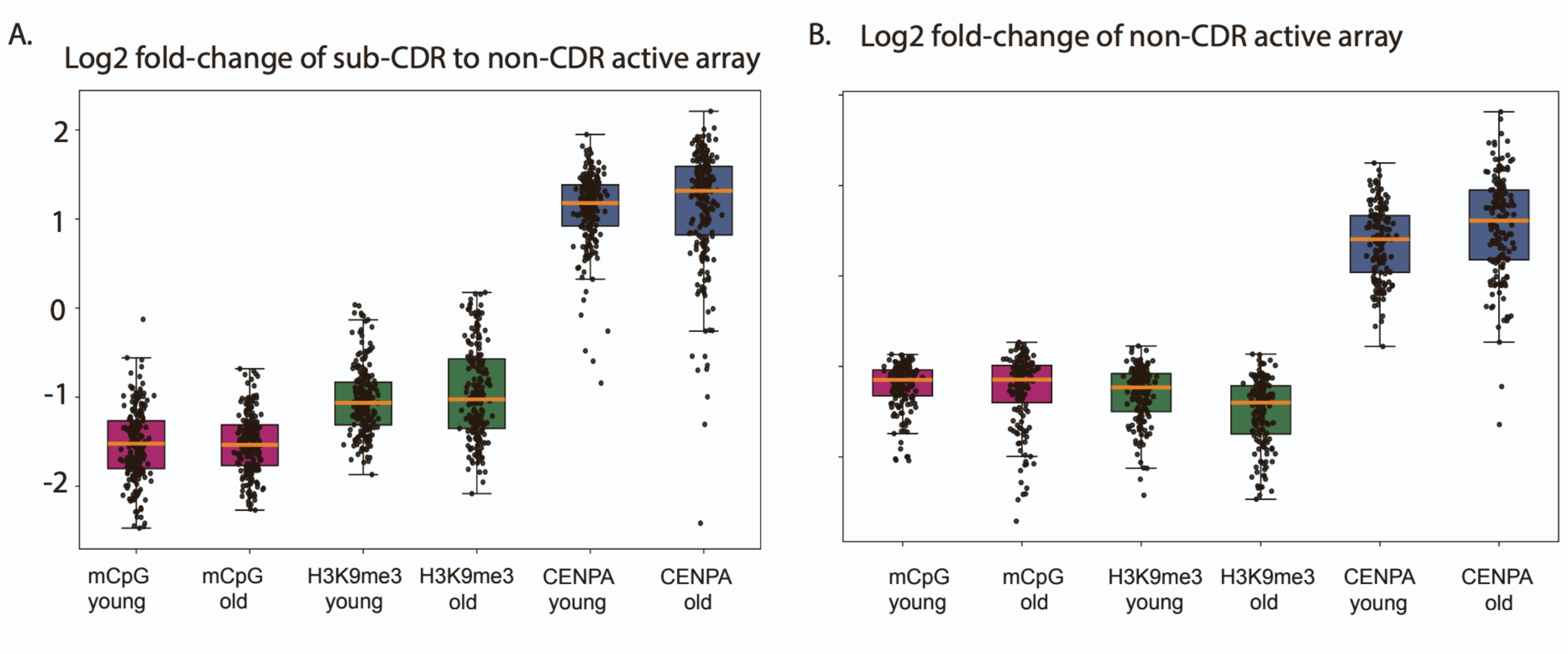

Figure S18: Enrichment within sub-CDRs across early- and late-passage, related to Figure 3
Epigenetic marks within general CDRs are stable between early- and late-passage LCLs. Box plots showing log2 fold-change of mCpG, H3K9me3, and CENP-A signal densities within sub-CDRs in early- and late-passage HG002 LCLs, calculated relative to chromosome-specific non-CDR active array baselines. Across all three marks, early- and late-passage cells show highly similar enrichment profiles within sub-CDRs: mCpG is comparably depleted in both conditions (median log2 fold-change ~−1.5), H3K9me3 is similarly reduced, and CENP-A is equivalently enriched, indicating that the relative epigenetic identity of the overall CDR domains is robustly maintained despite extended culture.

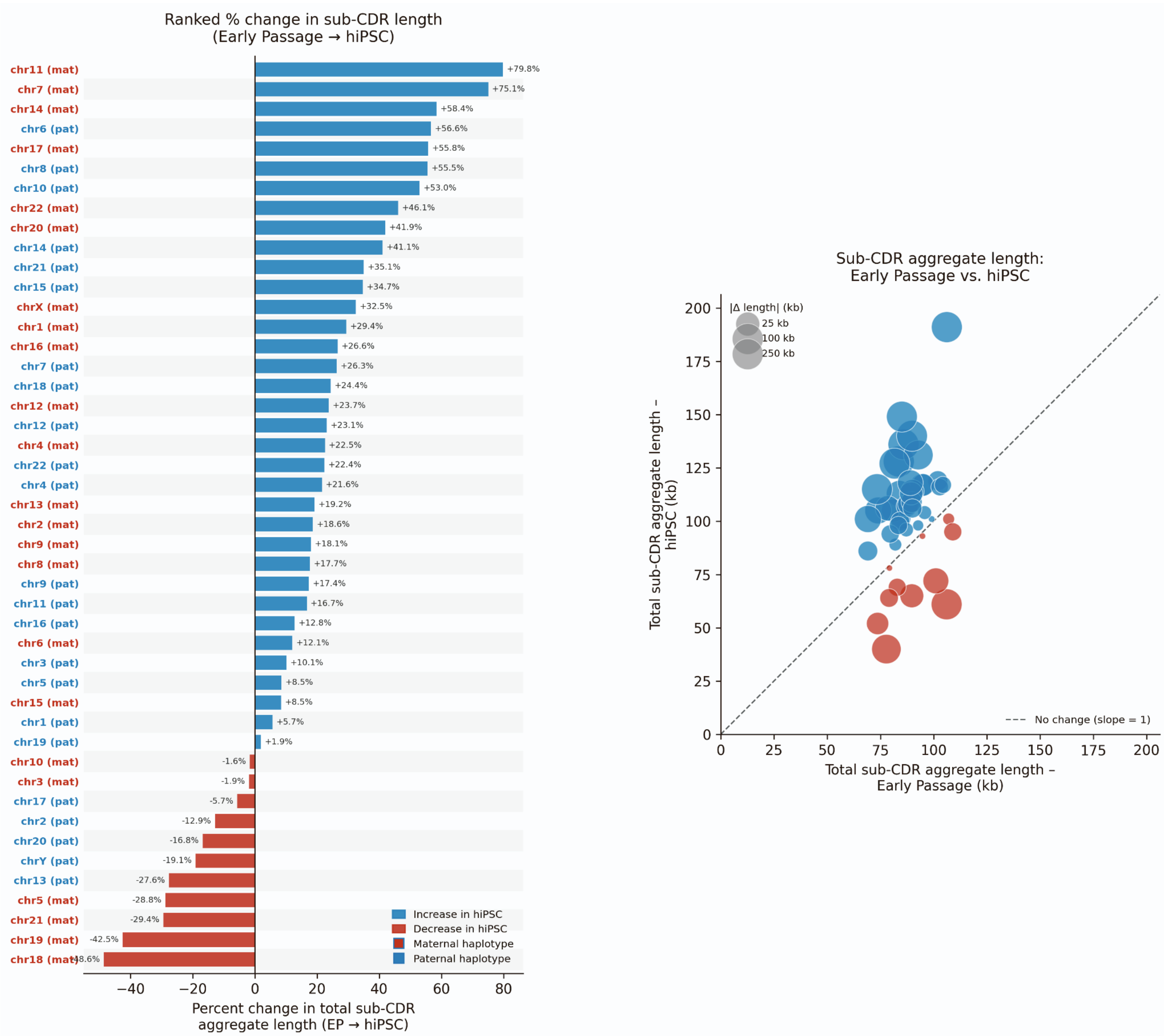

Figure S19: Comparison between HG002 early passage and hiPSC CDRs, related to Figure 3

(Left) Ranked percent change in total sub-CDR aggregate length between early passage (EP) and hiPSC, shown for all active αSat arrays across 46 haplotypes. Arrays are rank-ordered by magnitude of change; increases in hiPSC relative to EP are shown in blue and decreases in red. Maternal and paternal haplotypes are indicated by red and blue labels, respectively. (Right) Scatter plot comparing total sub-CDR aggregate length (kb) between EP (x-axis) and hiPSC (y-axis). Each point represents a single array; circle size is proportional to the absolute magnitude of change between conditions. The dashed line indicates the expectation under no change.

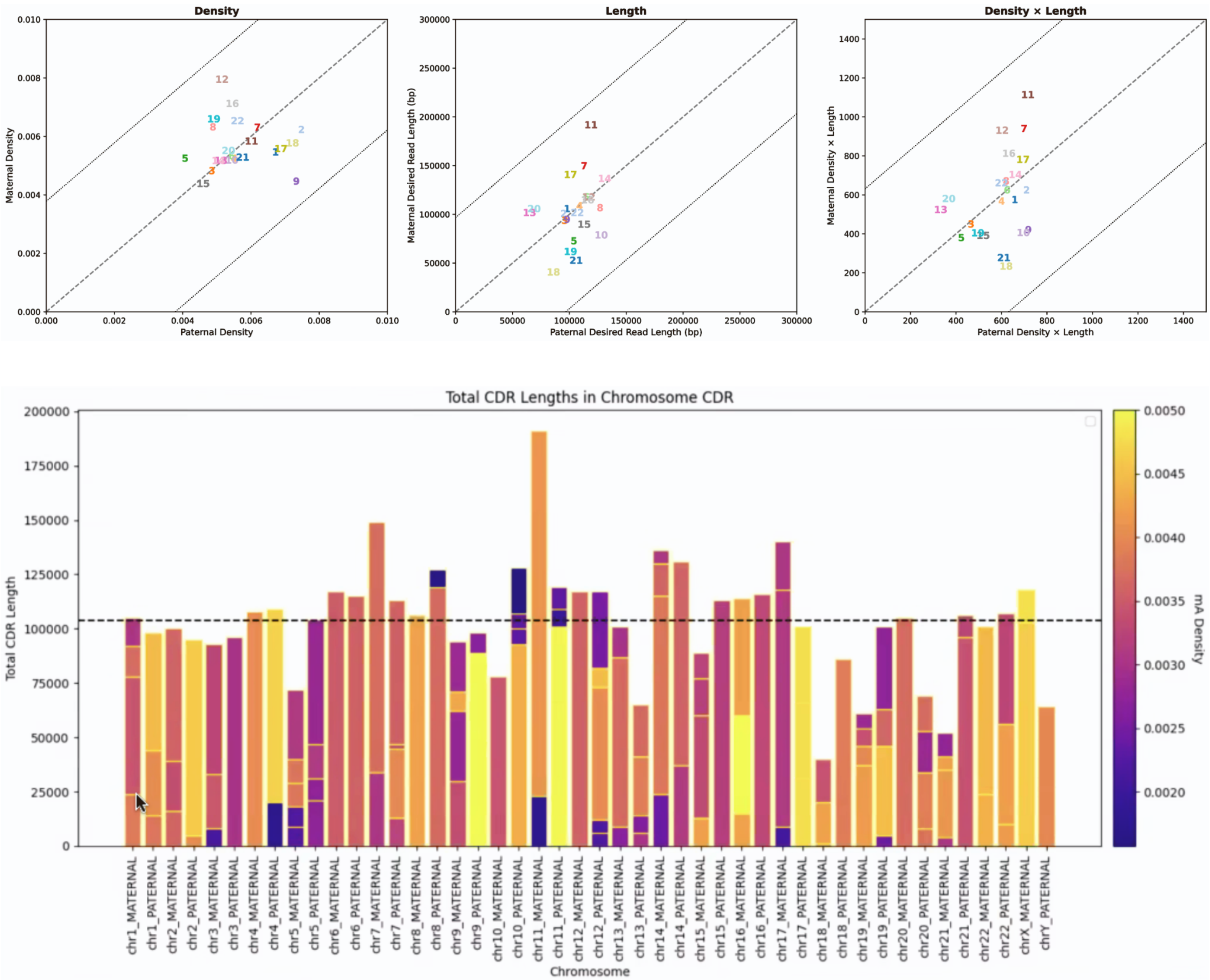

Figure S20: CENP-A DiMeLo-seq Density in hiPSCs, related to Figure 3

CENP-A chromatin dosage is balanced between homologous centromeres in PBMC-derived iPSCs. (A) Scatter plots comparing paternal (x-axis) and maternal (y-axis) CENP-A DiMeLo-seq 6mA signal density (left) and total sub-CDR length (right) across all chromosomes in PBMC-derived iPSCs. Each chromosome is labeled and color-coded. The dashed diagonal line represents the identity line; dotted lines indicate ±3 SD outlier thresholds. (B) Stacked bar plots showing total CDR length per chromosome for maternal and paternal haplotypes for late-passaged CENP-A DiMeLo-seq. Each bar is subdivided into individual sub-CDR segments colored by 6mA signal density using a plasma colormap. The dashed horizontal line indicates the mean total CDR length across all homologs. A color bar indicates the range of 6mA density values. Maternal and paternal haplotypes are displayed as adjacent pairs for each chromosome, ordered from chr1 to chrY.

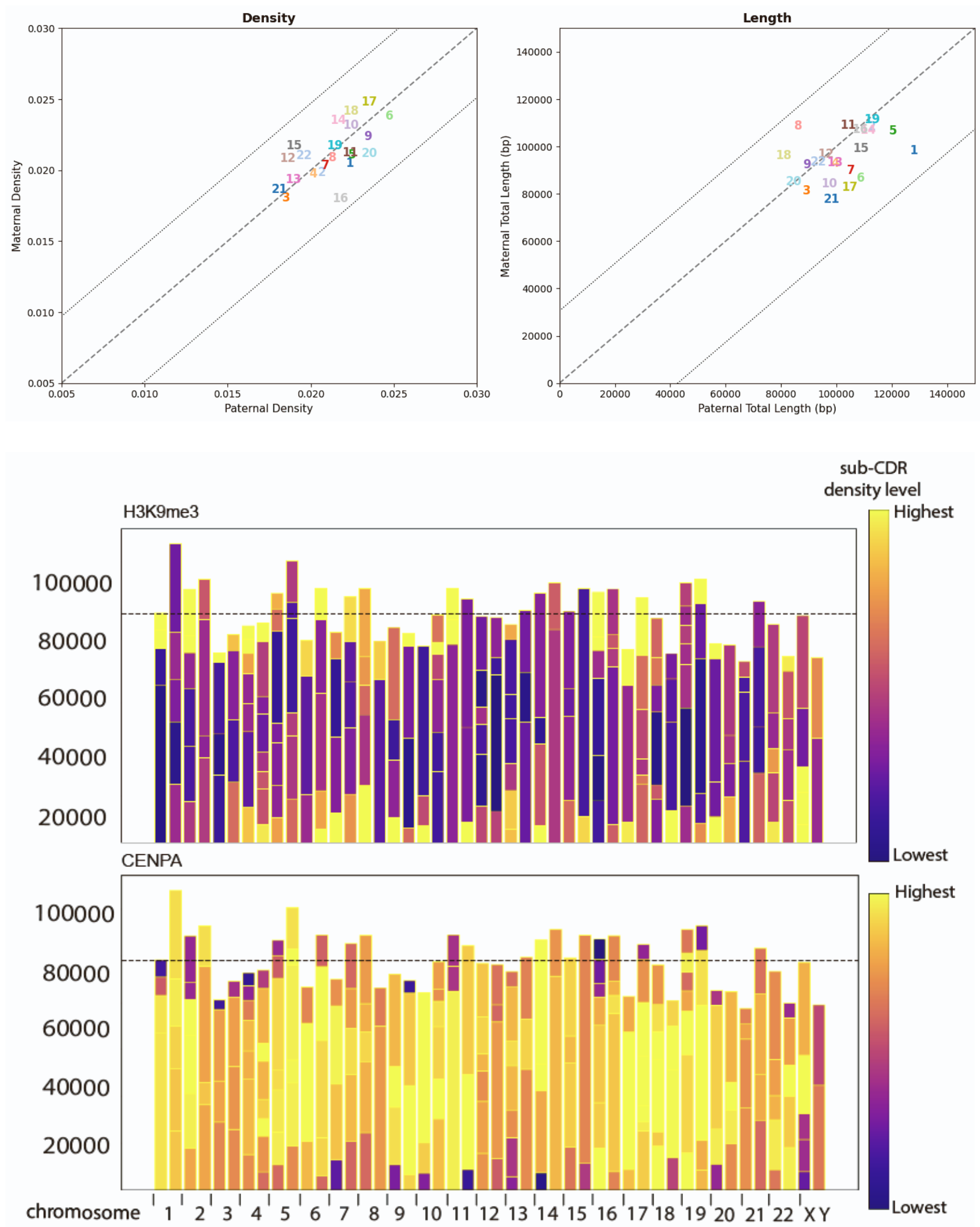

Figure S21: CENP-A DiMeLo-seq density in late passage lines, related to Figure 3

CENP-A chromatin dosage is balanced between homologous centromeres in late-passage LCLs. (A) Scatter plots comparing paternal (x-axis) and maternal (y-axis) CENP-A DiMeLo-seq 6mA signal density (left) and total sub-CDR length (right) across all chromosomes in late-passage HG002 LCLs. Each chromosome is labeled and color-coded. The dashed diagonal line represents the identity line; dotted lines indicate ±3 SD outlier thresholds. Chromosomes deviating beyond these thresholds represent haplotype-specific departures from dosage balance. (B) Stacked bar plots showing total CDR length per chromosome for maternal and paternal haplotypes for late-passaged CENP-A DiMeLo-seq. Each bar is subdivided into individual sub-CDR segments colored by 6mA signal density using a plasma colormap. The dashed horizontal line indicates the mean total CDR length across all homologs. A color bar indicates the range of 6mA density values. Maternal and paternal haplotypes are displayed as adjacent pairs for each chromosome, ordered from chr1 to chrY.

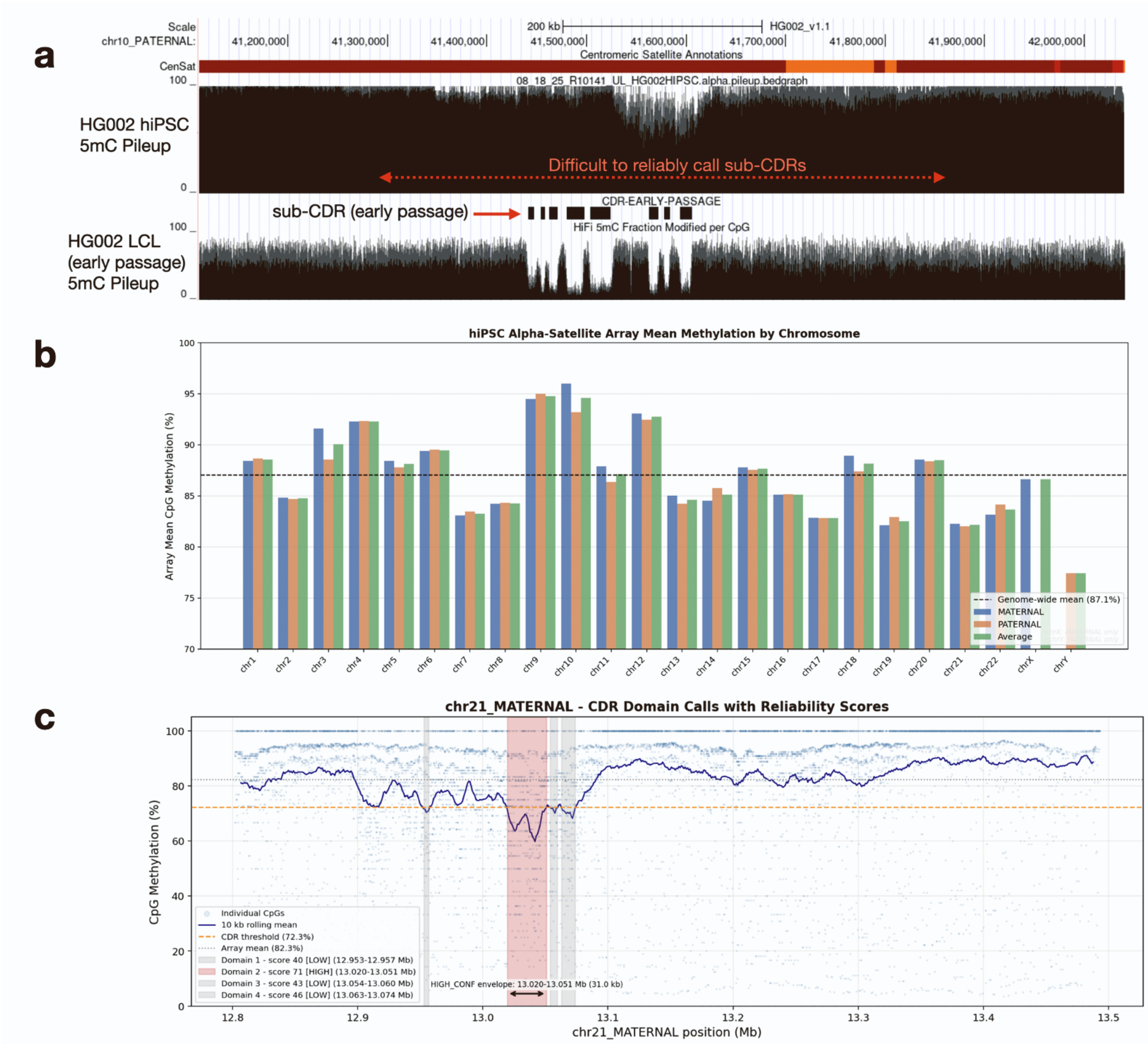

Figure S22: hiPSC array CpG density and CDR prediction, related to Figure 3.
Centromere dip regions (CDRs) are characterized by reduced CpG methylation relative to the surrounding active α-satellite higher-order repeat (HOR) array. HG002 hiPSC active arrays exhibit higher array-wide methylation than matched lymphoblastoid cell line (LCL) arrays (mean, 87% versus ~60%), complicating identification of sub-CDR boundaries. **(a)** UCSC Genome Browser visualization of the chromosome 10 paternal array illustrates the shallow methylation dip in hiPSCs (top) relative to early-passage cells (bottom), for which sub-CDR boundaries are difficult to define confidently. **(b)** For each haplotype-resolved α-satellite array, mean CpG methylation was calculated across all CpG sites and used to establish an array-specific baseline. The CDR detection threshold was defined as 10 percentage points below the array-wide mean methylation (CDR threshold = array mean methylation − 10%). To identify sub-CDRs in the hiPSC 5mC dataset, we developed a sliding-window CDR caller, illustrated for the chromosome 21 maternal array **(c)**. The caller identifies contiguous regions below the array-specific methylation threshold as distinct candidate domains and assigns each domain a composite reliability score to distinguish well-supported calls from marginal detections near the detection boundary. CDR calling was performed using phased, per-CpG methylation bedGraph files derived from Oxford Nanopore long-read sequencing and spanning the full assembled α-satellite array for each haplotype.

References

1. Raney, B.J., Dreszer, T.R., Barber, G.P., Clawson, H., Fujita, P.A., Wang, T., Nguyen, N., Paten, B., Zweig, A.S., Karolchik, D., et al. (2014). Track data hubs enable visualization of user-defined genome-wide annotations on the UCSC Genome Browser. Bioinformatics 30, 1003–1005. https://doi.org/10.1093/bioinformatics/btt637.

2. Sweeten, A.P., Schatz, M.C., and Phillippy, A.M. (2024). ModDotPlot—rapid and interactive visualization of tandem repeats. Bioinformatics 40, btae493. https://doi.org/10.1093/bioinformatics/btae493.

3. Stergachis, A.B., Debo, B.M., Haugen, E., Churchman, L.S., and Stamatoyannopoulos, J.A. (2020). Single-molecule regulatory architectures captured by chromatin fiber sequencing. Science 368, 1449–1454. https://doi.org/10.1126/science.aaz1646.

4. Muro, Y., Masumoto, H., Yoda, K., Nozaki, N., Ohashi, M., and Okazaki, T. (1992). Centromere protein B assembles human centromeric alpha-satellite DNA at the 17-bp sequence, CENP-B box. J. Cell Biol. 116, 585–596. https://doi.org/10.1083/jcb.116.3.585.

5. Jha, A., Bohaczuk, S.C., Mao, Y., Ranchalis, J., Mallory, B.J., Min, A.T., Hamm, M.O., Swanson, E., Dubocanin, D., Finkbeiner, C., et al. (2024). DNA-m6A calling and integrated long-read epigenetic and genetic analysis with fibertools. Genome Res. 34, 1976–1986. https://doi.org/10.1101/gr.279095.124.

6. Altemose, N., Maslan, A., Smith, O.K., Sundararajan, K., Brown, R.R., Mishra, R., Detweiler, A.M., Neff, N., Miga, K.H., Straight, A.F., et al. (2022). DiMeLo-seq: a long-read, single-molecule method for mapping protein-DNA interactions genome wide. Nat. Methods 19, 711–723. https://doi.org/10.1038/s41592-022-01475-6.
